# Safety and antitumor activity of metformin plus lanreotide in patients with advanced gastro-intestinal or lung neuroendocrine tumors: the phase Ib trial MetNET2

**DOI:** 10.1186/s13045-023-01510-9

**Published:** 2023-12-14

**Authors:** Sara Pusceddu, Francesca Corti, Natalie Prinzi, Federico Nichetti, Silva Ljevar, Adele Busico, Tommaso Cascella, Rita Leporati, Simone Oldani, Chiara Carlotta Pircher, Jorgelina Coppa, Veronica Resi, Massimo Milione, Marco Maccauro, Rosalba Miceli, Elena Tamborini, Federica Perrone, Carlo Spreafico, Monica Niger, Federica Morano, Filippo Pietrantonio, Ettore Seregni, Luigi Mariani, Vincenzo Mazzaferro, Giorgia Di Liberti, Giovanni Fucà, Filippo de Braud, Claudio Vernieri

**Affiliations:** 1 Department of Medical Oncology, Fondazione IRCCS Istituto Nazionale dei Tumori di Milano, ENETS Center of Excellence, Via Venezian 1, 20133 Milan, Italy; 2 Clinical Epidemiology and Trial Organization, Department of Applied Research and Technological Development, Fondazione IRCCS Istituto Nazionale Tumori Di Milano, Milan, Italy; 3 Department of Advanced Diagnostics, Fondazione IRCCS Istituto Nazionale dei Tumori di Milano, ENETS Center of Excellence, Milan, Italy; 4 Department of Radiology Foundation IRCCS Istituto Nazionale dei Tumori di Milano, ENETS Center of Excellence, Milan, Italy; 5 Hepato-Biliary-Pancreatic and Upper G.I. Surgery, Liver Transplantation and Hepato-Oncology Unit, Fondazione IRCCS Istituto Nazionale dei Tumori di Milano, ENETS Center of Excellence, Milan, Italy; 6 Endocrinology Unit, Fondazione IRCCS Ca’ Granda Ospedale Maggiore Policlinico, Milan, Italy; 7 Department of the Pathology and Laboratory Medicine, Fondazione IRCCS Istituto Nazionale dei Tumori di Milano, ENETS Center of Excellence, Milan, Italy; 8 Departement of Nuclear Medicine, Fondazione IRCCS Istituto Nazionale dei Tumori di Milano, ENETS Center of Excellence, Milan, Italy; 9 Department of Oncology and Hemato-Oncology, Università deli Studi di Milano, Milan, Italy; 10 IFOM ETS – The AIRC Institute of Molecular Oncology, Via Ademello 16, 20139 Milan, Italy; 11 SC Medical Oncology, Fondazione IRCCS San Gerardo Dei Tintori, Monza, Italy

**Keywords:** Well-differentiated neuroendocrine tumors (WDNETs), Metformin plus lanreotide, Safety, Antitumor activity, Phase Ib trial

## Abstract

In retrospective studies, metformin use has been associated with better clinical outcomes in diabetic patients with advanced, well-differentiated neuroendocrine tumors (WDNETs). However, prospective evidence of metformin safety and activity is lacking. Here, we conducted the first-in-human phase Ib MetNET2 trial to investigate the safety and antitumor activity of metformin in combination with the somatostatin analog lanreotide autogel (ATG) in both diabetic and non-diabetic patients with advanced WDNETs of the gastrointestinal (GI) or thoracic tract. Enrolled patients received lanreotide ATG 120 mg plus oral metformin, up to a maximum dosage of 2550 mg/day. We enrolled 20 patients, of whom 18 (90%) and 2 (10%) had WDNETs of the GI and thoracic tract, respectively. Fourteen patients (70%) were non-diabetic. With a 5% incidence of SAEs, the study met its primary objective of demonstrating treatment safety. With a median follow-up of 39 months (95% CI 28-NE), median PFS was 24 months (95% CI 16-NE), with 12-month and 24-month PFS probability of 75% (95% CI 58–97) and 49% (95% CI 31–77), respectively. We found no statistically significant PFS differences between diabetic and non-diabetic patients. Among exploratory analyses, the presence of tumor genomic alterations in DNA damage pathways was associated with trend towards worse PFS, whereas a precocious reduction of HOMA-IR index and plasma cholesterol concentration showed a trend towards an association with better PFS. In conclusion, metformin plus lanreotide ATG is a safe and well tolerated combination treatment that is associated with promising antitumor activity in both non-diabetic and diabetic patients with WDNETs, and that warrants further investigation in larger clinical trials.

**Supplementary Information:**

The online version contains supplementary material available at 10.1186/s13045-023-01510-9.

**To the Editor**

Somatostatin analogs (SSAs) are the mainstay of treatment for patients with advanced, well-differentiated NETs (WDNETs) expressing somatostatin receptors [1, 2]. The antitumor effects of SSAs in WDNETs are in part mediated through the inhibition of the PI3K/AKT/mTOR and MAPK pathways [3]. In retrospective studies, the use of the antidiabetic compound metformin in diabetic patients with advanced WDNETs was associated with better clinical outcomes when combined with standard SSAs plus/minus everolimus [4–7]. However, no prospective evidence exists to support metformin use in combination with SSAs in advanced WDNET patients with or without diabetes mellitus (DM). Based on these premises, we conducted MetNET-2, a first-in-human, phase Ib clinical trial that investigated the safety and antitumor activity of experimental metformin in combination with standard lanreotide autogel (ATG) in both diabetic and non-diabetic patients with advanced GI or thoracic WDNETs. The primary study objective was to assess the safety of the experimental treatment, as defined as the incidence of serious adverse events (SAEs). Study Methods are reported in Additional file 1.

Between April 2016 and April 2019 we enrolled a total number of 20 patients, whose characteristics are summarized in Additional file 2: Table S1. Of these, six patients (30%) had a prior diagnosis of DM. Median duration of exposure to the experimental treatment was 15.9 months (IQR range, 5.8–21.0). Study drug exposure is reported in Additional file 3: Fig. S1 and Additional file 4: Table S2. With only one treatment-related SAE (5%, acute renal failure), MetNET-2 met its primary endpoint demonstrating the safety of metformin plus SSAs. The renal SAE was likely to be multifactorial (G2 hypertension NDR, G1 urolithiasis NDR), with a possible contribution of metformin-related pre-renal kidney injury from G1 diarrhea. Of note, this toxicity was reversible after temporary interruption of metformin administration and the initiation of anti-hypertensive therapy. The most common any-grade treatment-related AEs (trAEs) were diarrhea (75%), hyperglycaemia (55%), asthenia (40%) and hypercholesterolemia (40%) (Table 1). Grade 3 trAEs occurred in 15% of patients, and consisted of acute renal failure (*n* = 1; 5%), diarrhea (*n* = 1; 5%) and abdominal pain (*n* = 1; 5%). No grade ≥ 4 trAEs were reported. trAE incidence was not significantly different in diabetic versus non-diabetic patients (Additional file 5: Table S3). In addition, no trAEs led to the discontinuation of Lanreotide ATG or metformin. Treatment-emergent AEs are reported in Additional file 6: Table S4.

**Table 1:** Most common treatment-related adverse events (trAEs)

Specific AEs	Any grade	Grade 1	Grade 2	Grade 3	Grade 4
Diarrhea	15 (75.0%)	5 (25.0%)	9 (45.0%)	1 (5.0%)	0 (0.0%)
Hyperglycemia	11 (55.0%)	11 (55.0%)	0 (0.0%)	0 (0.0%)	0 (0.0%)
Asthenia	8 (40.0%)	4 (20.0%)	4 (20.0%)	0 (0.0%)	0 (0.0%)
Hypercholesterolemia	8 (40.0%)	8 (40.0%)	0 (0.0%)	0 (0.0%)	0 (0.0%)
Hypomagnesaemia	7 (35.0%)	6 (30.0%)	1 (5.0%)	0 (0.0%)	0 (0.0%)
Abdominal pain	5 (25.0%)	2 (10.0%)	2 (10.0%)	1 (5.0%)	0 (0.0%)
Anorexia	5 (25.0%)	4 (20.0%)	1 (5.0%)	0 (0.0%)	0 (0.0%)
Creatinine increase	4 (20.0%)	4 (20.0%)	0 (0.0%)	0 (0.0%)	0 (0.0%)
Nausea	4 (20.0%)	3 (15.0%)	1 (5.0%)	0 (0.0%)	0 (0.0%)
Emesis	4 (20.0%)	3 (15.0%)	1 (5.0%)	0 (0.0%)	0 (0.0%)
Hypertriglyceridemia	4 (20.0%)	3 (15.0%)	1 (5.0%)	0 (0.0%)	0 (0.0%)
Hyperuricemia	3 (15.0%)	3 (15.0%)	0 (0.0%)	0 (0.0%)	0 (0.0%)
Hyponatremia	2 (10.0%)	2 (10.0%)	0 (0.0%)	0 (0.0%)	0 (0.0%)
Steatorrhea	2 (10.0%)	0 (0.0%)	2 (10.0%)	0 (0.0%)	0 (0.0%)
Intestinal bloating	2 (10.0%)	2 (10.0%)	0 (0.0%)	0 (0.0%)	0 (0.0%)
GGT elevation	2 (10.0%)	1 (5.0%)	1 (5.0%)	0 (0.0%)	0 (0.0%)
Dysgeusia	1 (5.0%)	1 (5.0%)	0 (0.0%)	0 (0.0%)	0 (0.0%)
Hypokalemia	1 (5.0%)	0 (0.0%)	1 (5.0%)	0 (0.0%)	0 (0.0%)
Hypophosphatemia	1 (5.0%)	1 (5.0%)	0 (0.0%)	0 (0.0%)	0 (0.0%)
Decreased appetite	1 (5.0%)	1 (5.0%)	0 (0.0%)	0 (0.0%)	0 (0.0%)
Acute renal failure	1 (5.0%)	0 (0.0%)	0 (0.0%)	1 (5.0%)	0 (0.0%)

The antitumor activity of the experimental treatment is summarized in Additional file 7: Table S5, Additional file 8: Fig. S2 and Additional file 9: Fig. S3. ORR was 10% (95% CI 1–32%) and DCR was 85% (95% CI 62–96%). With a median follow-up of 39 months (95% CI 28 months-NE), median PFS was 24 months (95% CI 16-NE months) (Fig. 1A), with 12-month and 24-month PFS probability of 75% (95% CI 58–97%) and 49% (95% CI 31–77%), respectively. Median TTP was 26 months (95% CI 17-NE months) (Fig. 1B). Diabetic status was not significantly associated with PFS (Additional file 10: Fig. S4). With the exception of non-functioning tumor status, which was associated with a lower risk of disease progression, none of the other clinico-pathological characteristics showed an association with PFS (Additional file 11: Table S6).

**Fig. 1:**
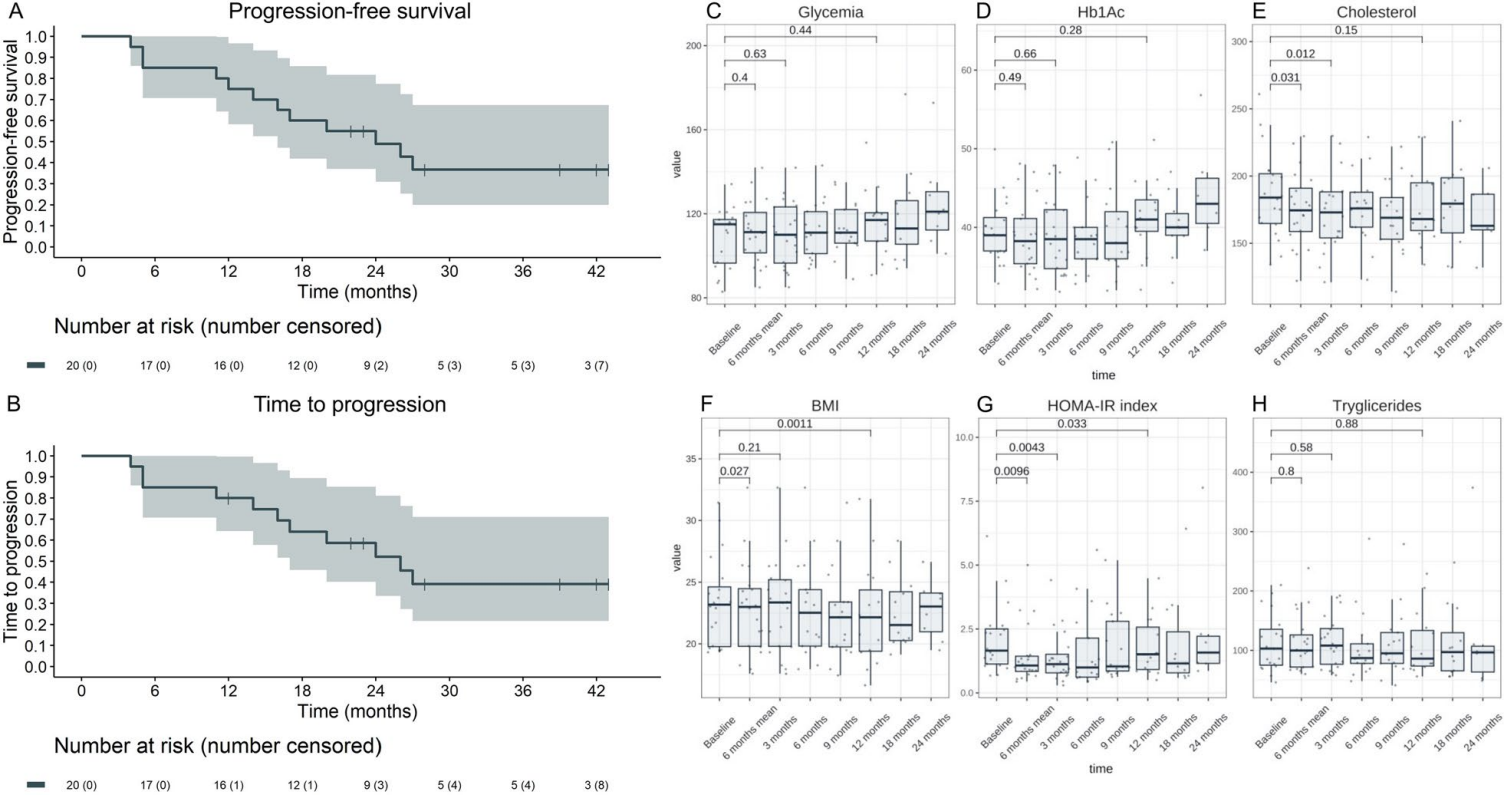
Kaplan–Meier curves for progression-free survival (PFS) (**A**) and time-to-progression (TTP) in the MetNET2 study cohort (**B**). Box plots depicting changes in the indicated metabolic parameters during the experimental treatment (**C**–**H**). The p value in panels **C**–**H** refers to the paired t test for the indicated comparisons

Then, we explored the potentially prognostic role of tumor genomic alterations, as evaluated through NGS analysis (Additional file 12: Fig. S5). Genomic alterations were not differently distributed between diabetic and non-diabetic patients (Additional file 13: Table S7). We found no statistically significant PFS differences between patients harboring any tumor genomic alteration and patients with wild-type genomic profiles (mPFS: 24 months [95% CI 14-NA] vs. 26 months; [95% CI 5-NA]; HR 0.61, 95% CI 0.19–1.96*, p* = 0.42). Interestingly, patients harboring tumor alterations in genes involved in DNA repair showed a trend towards worse PFS when compared to patients without alterations in DNA repair genes (median PFS 13 months [95% CI 11-NA] *vs.* 27 months [95% CI 20-NA]; HR 2.74; 95% CI 0.85–8.81; *p* = 0.09) (Additional file 14: Fig. S6A), whereas *FGFR4* gene polymorphisms or *ATM* gene alterations were not associated with patient PFS (Additional file 14: Fig. S6B–D).

In the whole 24 months follow-up period, we observed no significant changes in any of the metabolic parameters evaluated (Fig. 1C–H; Additional file 15: Table S8). The HOMA-IR index was reduced 3 months after treatment initiation, whereas patient BMI and plasma cholesterol levels were reduced within 6 months (Fig. 1C–H; Additional file 15: Table S8). Patients experiencing higher early reduction of HOMA-IR index and plasma cholesterol concentration showed a trend towards better PFS (*p* = 0.055 and *p* = 0.086, respectively, Additional file 16: Table S9).

In conclusion, metformin plus lanreotide ATG is safe, well tolerated and active in both non-diabetic and diabetic patients with WDNETs of the GI or thoracic tract. A precocious reduction of HOMA-IR index and plasma cholesterol may predict higher clinical benefit from this treatment. Larger, prospective clinical trials should be conducted to investigate if adding metformin to SSAs results in outcome improvement when compared to SSAs alone in this clinical setting.

### Supplementary Information

**Additional file 1.** Study Methods.

**Additional file 2. Table S1**: Demographic, clinic-pathological and metabolic characteristics of enrolled patients.

**Additional file 3. Fig. S1:** Spaghetti plots of dose intensity for metformin administration (A) and for dose intensity of Lanreotide ATG (B).

**Additional file 4. Table S2**: Metformin and Lanreotide ATG drug exposure and relative dose intensity according to diabetic status.

**Additional file 5. Table S3**: Differences in the incidence of trAEs between non-diabetic patients (N=14) and diabetic patients (N=6).

**Additional file 6. Table S4**: Treatment-Emergent Adverse Events (TE-AEs).

**Additional file 7. Table S5**: Antitumor activity of the experimental treatment.

**Additional file 8. Fig. S2**: Duration of treatment and response assessment by RECIST 1.1 criteria after Central imaging review.

**Additional file 9. Fig. S3**: Waterfall plot showing the relative modification of the estimated tumor volume (as per RECIST 1.1 criteria), achieved as the best tumor response.

**Additional file 10. Figure S4**: Kaplan-Meier curves for PFS among diabetic and non-diabetic patients (A). Kaplan Meier curves for PFS in normoglycemic, pre-diabetic and diabetic patients (B).

**Additional file 11. Table S6**: Univariate Cox models for PFS.

**Additional file 12. ** **Figure S5.** Oncoprint of tumor genomic alterations in patients enrolled in Met-NET2 trial. Mutations are classified as missense mutations (green), truncating mutations (dark grey), or no alterations (light grey).

**Additional file 13. **Table S7. Distribution of genomic biomarkers according to diabetic status.

**Additional file 14. Figure S6**: Figure S6. Kaplan-Meier curves for PFS according to the presence of alterations in genes involved in DNA repair (ARID1A, ATM, SETD2, PRKDC) (A), FGFR4 gene polymorphism rs351855 (B), ATM allelic variants: A/A vs. A/C vs. C/C (C), or A/A vs. A/C or C/C (D).

**Additional file 15. Table S8: **Longitudinal kinetics of the indicated metabolic blood parameters and body mass index (BMI).

**Additional file 16. Table S9:** Association between early reduction in metabolic parameters and the risk of disease progression (Hazard Ratio).

## Data Availability

The datasets used and/or analysed during the current study are available from the corresponding author on reasonable request.

## Abbreviations

DCR: Disease control rate
DM: Diabetes mellitus
GI: Gastro-intestinal
HR: Hazard ratio
IEC: Independent Ethics Committee
IQR: Interquartile range
IRB: Institutional Review Board
Lanreotide ATG: Lanreotide autogel
mTOR: Mammalian target of rapamycin
NGS: Next generation sequencing
OGTT: Oral Glucose Tolerance Test
ORR: Overall response rate
OS: Overall survival
PD: Progression of disease
PFS: Progression free survival
PR: Partial response
PRRT: Peptide receptor radiotherapy
RDI: Relative dose intensity
RECIST: Response evaluation criteria in solid tumors
SAEs: Serious adverse events
SD: Stable disease
SSAs: Somatostatin analogs
trAEs: Treatment-related adverse events
TTP: Time-to-progression
WDNETs: Well-differentiated neuroendocrine tumors
