# Supplementary material for: Safety and antitumor activity of metformin plus lanreotide in patients with advanced gastro-intestinal or lung neuroendocrine tumors: the phase Ib trial MetNET2
Source: J Hematol Oncol. 2023 Dec 14;16:119. doi: 10.1186/s13045-023-01510-9 (PMC10722662; doi:10.1186/s13045-023-01510-9)
Supplement: Supplementary file 1 — Additional file 1. Study Methods. [file 13045_2023_1510_MOESM1_ESM.docx]

**ADDITIONAL FILE 1**

**Study Methods**

**Study design**

The MetNET-2 trial (Clinicaltrials.gov identifier: NCT02823691) is an open-label, dose-finding, single-arm, monocentric, phase Ib clinical trial that was designed to investigate the safety, tolerability and antitumor activity of oral metformin in combination with lanreotide ATG in patients with advanced WDNETs of the GI tract or the lung.

**Enrollment criteria**

Main inclusion criteria were: age ≥ 18 years; histologically documented diagnosis of advanced WDNETs of the GI or thoracic (including lung, thymus typical and atypical carcinoids) tract, as defined according to the 2019-2021 World Health Organization Classification criteria ^1,2^; functioning or non-functioning tumors; measurable disease; evidence of disease progression (according to response evaluation criteria in solid tumors [RECIST] version 1.1) ^3^ within 6 months before the initiation of the study treatment; documented pathological uptake of Octreoscan/Ga68 Positron Emission Tomography (PET), or immunohistochemical intratumor expression of somatostatin receptor SSTR2, as assessed within 6 months before study entry; adequate bone marrow and organ function; treatment-naïve patients, or patients who had previously received local and/or systemic therapy, including surgery, chemotherapy, other SSAs (such as octreotide acetate LAR), mTORC1 inhibitors or peptide receptor radiotherapy (PRRT).

We enrolled patients who were either normoglycemic or had type-2 diabetes mellitus (DM), which was defined as: fasting plasma glucose level ≥ 126 mg/dL, hemoglobin (Hb)A1c ≥ 6.5% (48 mmol/mol), plasma glucose concentration > 200 mg/dL after Oral Glucose Tolerance Test (OGTT), or the finding of plasma glucose concentration ≥ 200 mg/dL (11.1 mmol/L) at random blood assessment in presence of symptoms of hyperglycemia or hyperglycemic crisis. Pre-diabetes was defined as fasting plasma glucose concentration of 100-125 mg/dL (impaired fasting glucose - IFG), or HbA1c of 5.7-6.4% (39-47 mmol/L) or plasma glucose concentration of 140-199 mg/dL after OGTT (impaired glucose tolerance – IGT).

Patients were ineligible if they had poorly differentiated G3 neuroendocrine carcinomas, if they had type 2 DM already treated with metformin (with the exception of patients who initiated metformin less than 6 months before study entry), or if they were taking other antidiabetic medications, including, but not limited to insulin, sulfonylureas, meglitinide, thiazolidinediones, dipeptidyl peptidase 4 (DPP-4) inhibitors, sodium glucose cotransporter inhibitors (SGLT2) or α-glucosidase inhibitors before initiation of the experimental treatment.

**Study objectives and endpoints**

The primary objective of MetNET2 was to assess the safety of the experimental treatment, which was measured by calculating the incidence of serious adverse events (SAEs).

Secondary study endpoints were: a) treatment tolerability, as defined as the incidence of severe (G3 or G4) treatment-related AEs (trAEs) or treatment-emergent AEs (TEAEs), which were graded according to the US National Cancer Institute (NCI) Common Terminology Criteria for Adverse Events (CTCAE; version 4.03); b) Progression Free Survival (PFS), as defined as the time between metformin plus lanreotide ATG treatment initiation and the detection of clinical/radiological disease progression (according to RECIST 1.1 criteria) or patient death from any cause, whichever occurred first; c) Time-To-Progression (TTP), as defined as the time interval between study treatment initiation and the detection of radiological or clinical disease progression (according to RECIST 1.1 criteria); d) Overall Response Rate (ORR), as defined as the ratio between the number of patients achieving complete response (CR) or partial response (PR) as their best response according to RECIST 1.1 criteria and the total number of patients; e) Disease Control Rate (DCR), as defined as the ratio between the number of patients achieving CR, PR or stable disease (SD) as their best response according to RECIST 1.1 criteria and the total number of enrolled patients; f) Overall Survival (OS), as defined as the time between treatment initiation and patient death from any cause. As exploratory analyses, we evaluated the association between the onset of early (i.e., first six months) on-treatment modifications of systemic metabolic or anthropometric parameters (e.g., plasma glucose, Hb1Ac, cholesterol, body mass index, HOMA-IR index, triglycerides) and PFS outcomes. We also investigated the association between specific tumor genomic alterations and patient PFS.

**Sample size and statistical plan**

A single-stage A’Hern design was used for sample size calculation. The null hypothesis that the SAE rate related to treatment is 25% was tested against a one-sided alternative. With twenty patients enrolled, the null hypothesis would have been rejected if no more than two patients (10%) experienced a trSAE. This design yields a type I error rate of 10% and a power of 85% with a maximum of toxicity rate of 5%.

**Ethical declarations**

Written informed consent was obtained from all participating subjects as a prerequisite for study enrollment. The trial was conducted in full conformance with the International Conference on Harmonisation (ICH) E6 guidelines for Good Clinical Practice (GCP), the principles of the Declaration of Helsinki and local laws and regulations ^4^. The study was approved by the Institutional Review Board/Independent Ethics Committee (IRB/IEC) of the Coordinating Center (Fondazione IRCCS Istituto Nazionale dei Tumori di Milano), and it was registered in EudraCT (registration number: 2015-004626-34) and *clinicaltrials.gov* (registration number: NCT02823691) databases.

**Statistical analysis**

Patient characteristics were analyzed by descriptive statistics. Continuous data were summarized as means and standard deviations, or as median and interquartile range (IQR) values. Categorical data were summarized as frequencies and percentages. Fisher's exact test was used for categorical variables, and Wilcoxon-Mann-Whitney test was used for continuous variables.

PFS, TTP and OS survival curves were estimated using the Kaplan-Meier method, and groups were compared using the log-rank test. Univariable Cox regression models were fitted to investigate the association between baseline clinical or pathological covariates and patient PFS; results of these models were summarized in terms of Hazard Ratios (HR), 95% confidence intervals (CI) and Wald test p-values.

Treatment exposure was defined as the number of days on study treatment over the whole study interval duration. Relative Dose Intensity (RDI) was defined as (dosing unit/unit of time)/planned dose. However, as the administered dose of metformin may repeatedly change throughout the study conduction and different metformin doses may be administered during different time intervals, the varying intensity and duration of each dose administration was considered. Assuming that k different doses were administered to a patient throughout the study, the RDI for each patient was calculated as a sum of terms, each of which accounts for the relative dose intensity for the period a certain dose i (i=1, …, k) of metformin was administered. In particular, it was calculated as:

$$RDI=\sum_{i=1}^{k} \frac{\#days dose i was administered}{total treatment duration}*\frac{the administered dose intensity}{planned dose}$$

As for exploratory analyses, one-way repeated measures ANOVA and paired *t* tests were used to compare baseline and on-treatment metabolic parameters after normality check and after adjusting p values for multiple comparisons through the Benjamini–Hochberg procedure. Metabolite measurements obtained during the first 3 and 6 months were summarized as early average level (i.e., the arithmetic mean of the two). The effect of early metabolic changes on PFS was assessed through Cox regression modeling. In detail, the impact of early changes in specific metabolic parameters was evaluated by testing baseline and early average measurements, along with their interaction, with nonlinear effects handled by means of restricted cubic splines, as previously described ^5^. Individual patients were categorized as having “high” vs “low” metabolic dysregulation by dichotomizing the difference between baseline and early average values, with the optimal cutoff identified by means of the maximally selected rank statistic.

All statistical tests were two-tailed. For primary endpoint analysis, a significance level of 5% was adopted; given the limited number of cases, secondary analyses were interpreted with less conservative thresholds of significance. The R software [R 4.2.0 (2022-04-22)] was used for statistical analyses.

**Study procedures and treatment**

**
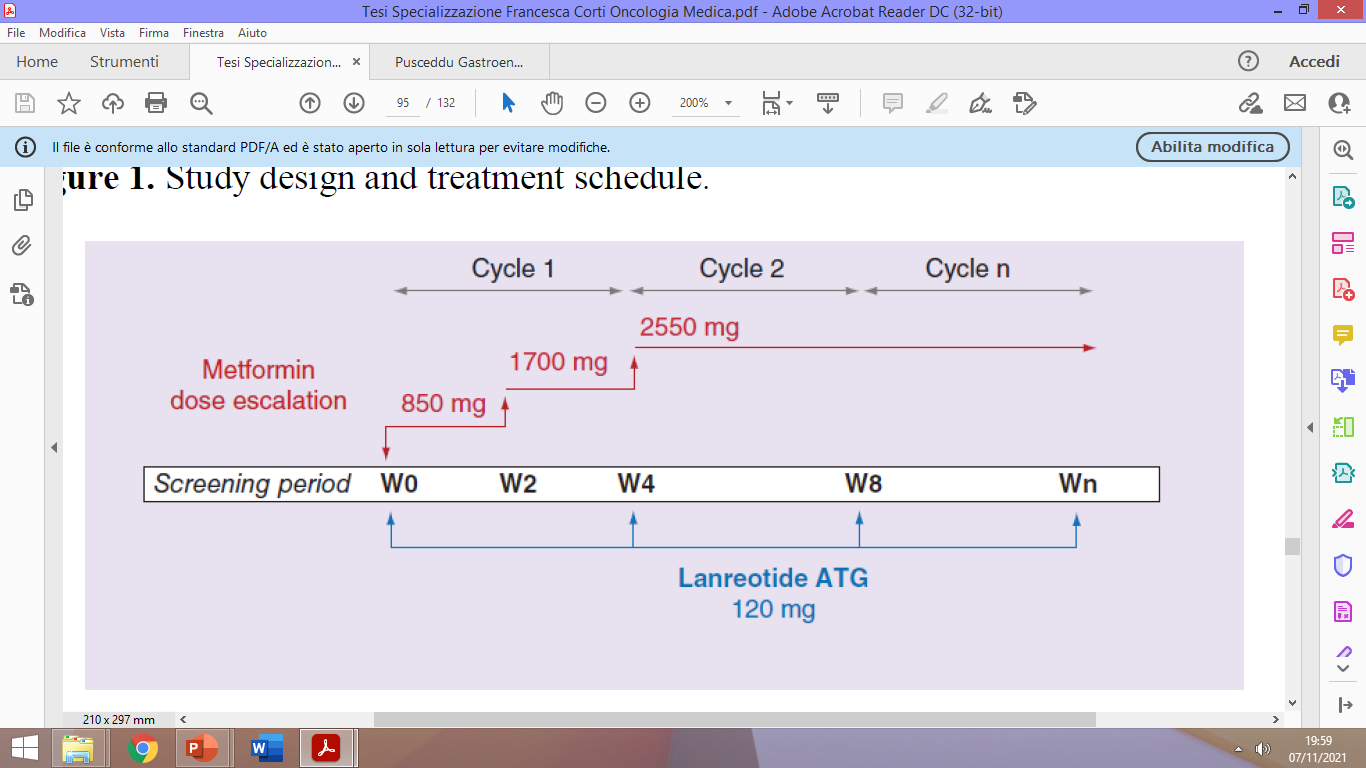
**Patients received lanreotide ATG 120 mg, as administered as deep subcutaneous injection every 28 days, plus oral metformin at the starting dose of 850 mg/day. If the treatment was well tolerated, metformin dosage was escalated up to 1700 mg/day at day 14, and, eventually, up to 2550 mg/day at day 28 (maximum dose). The trial design is illustrated below.

Legend: W: week

If the patient did not tolerate the escalating or maximum metformin dose, metformin dose was reduced to the previous dose level, and treatment tolerability was re-assessed. The experimental treatment was continued until the documentation of disease progression, intolerable toxicity, patient consent withdrawal or investigator decision. Metformin tablet counts at each visit were used to evaluate patient’s adherence to the experimental treatment.

Baseline assessments included demographics, clinical and physical examination, assessment of concomitant therapies, OGTT and blood tests (hematology analysis, renal function with estimated glomerular filtration rate, electrolyte levels, liver function, thyroid function, fasting plasma glucose, cholesterol and triglyceride levels, venous blood gas analysis, urinalysis, dosage of blood HbA1c, C-peptide or insulin concentration, quantification of blood vitamin B12, chromogranin A [CgA], neuron-specific enolase [NSE], 5-Hydroxyindoleacetic acid [5-HIAA] concentration, and assessment of hepatitis B and C serology and pregnancy test. The homoeostasis model assessment of insulin resistance (HOMA-IR; fasting plasma glucose [mmol/L]×fasting insulin [mU/L])/22.5)^6^ was performed at baseline and at pre-defined timepoints after study treatment initiation (i.e., at 3, 6, 9, 12, 18 and 24 months) to define the metabolic glycemic status of patient population.

A 12-lead electrocardiogram (ECG) and radiological assessment of the chest, abdomen and pelvis by contrast-enhanced computed tomography [CE-CT] or magnetic resonance imaging [MRI] were obtained within 28 days from the initiation of the study treatment. All additional suspected sites of disease (brain, bone) were imaged at baseline according to investigator’s judgment.

Enrolled patients were evaluated for treatment safety and tolerability by performing clinical and physical examination, blood test evaluation including blood cell counts, fasting plasma glucose, insulin and lipid profile quantification, renal and liver function assessment, venous blood gas and urinalysis, collection and examination of reported treatment-related adverse events (trAEs) and treatment-emergent adverse events (TE-AEs), or severe adverse events (SAEs) and concomitant medication at each visit; every 14 days for the first two cycles, and every 28 days thereafter.

Thyroid parameter analysis, dosages of blood vitamin B12, c-peptide, HbA1c, CgA, NSE and 5-HIAA levels were performed every 3 months.

Radiological assessment was performed every 4 months. A blinded Local Review of imaging according to RECIST criteria 1.1 was performed. All patients who discontinued the study drug(s) for any reason other than disease progression or death continued to be evaluated through clinical and radiological assessment as per study schedule until documented disease progression, death or until the initiation of a new line of anticancer therapy.

Tumor genomic profiling was performed through targeted Next Generation Sequencing (NGS) for all enrolled patients. In particular, we evaluated the prognostic role of genomic biomarkers potentially implicated in tumor sensitivity to metformin and lanreotide, and in particular to signaling cascades involved in MAPK and mTORC1 pathways. Formalin-fixed paraffin embedded (FFPE) tumor samples were analyzed using a custom NGS panel (IonTorrent Technology®) covering the coding sequences of 111 genes, including *LKB1, TP53, KRAS, BAX, IGF1R, VEGFR, PDGFR*, *AKT, PIK3CA, PTEN, mTOR, TSC2* and *NF1*. The complete gene panel and analytical details are provided in the **Appendix** at the end of this Additional File. The DNA extracted from the peripheral blood lymphocytes of each patient was subjected to NGS analysis and considered as control DNA in order to distinguish somatic mutations in potentially pathogenic

genes from gene polymorphisms. Peripheral blood samples for biomarker analysis were collected on the first day of the first therapy cycle.

**Complete gene panel for targeted next generation sequencing (NGS) analyses.**

5 µm cut FFPE tumor sections were manually microdissected to isolate the highest percentage of neoplastic cells. Genomic DNA was extracted by using the GeneRead DNA FFPE kit (Qiagen, Hilden, Germany, http://www.qiagen.com Cat. n. 180134). DNA amount was quantified with Qubit dsDNA Broad Range kit and the QuBit 3.0 instrument (both Thermo Fisher) following the manufacturer’s instructions.

gDNA (10ng) was profiled using a customized panel (*Ampliseq* Designer, ThermoFisher) that amplify 111 genes including 57 complete coding genes (*ARID1A, ATRX, BAP1, BAX, BLM, BRACHYURY (T), CDK4, DAXX, FANCM, FGFR4, FHIT, FOXA1, GATA3, GRM3, IGF1R, IGF2R, KDM5C, MED12, MEN1, MLL3, MSH2, MSH6, mTOR, NF1, PBRM1, PDGFRB, PIK3R1, PMS2, POLK, PRKDC, PTCH1, RAC1, RAD50, RIT1, RUNX1, RUNX3, SDHA, SDHB, SDHC, SDHD, SETD2, SMAD2, SMAD3, SNX31, SPOP, TBX3, TERT, TGFBR2, TNF ALFA, TR2/ TXNRD3, TSC1, TSC2, VEGFR1/ FLT1, VEGFR3/ FLT4, WT1), and mutation hotspots in 54 genes (ABL1, AKT1, ALK, APC, ARID2, ATM, BRAF, CDH1, CDKN2A, CSF1R, CTNNB1, DDR2, EGFR ,EZH2, FBXW7, FGFR1, FGFR2, FGFR3, FLT3, GNA11, GNAQ, GNAS, HER2, HER4, HNF1A, HRAS, IDH1, IDH2, JAK2, JAK3, KIT, KRAS, MEK1 (MAP2K1), MEK4 (MAP2K4), MET, MLH1, MPL, NOTCH1, NPM1, NRAS, PDGFRA, PIK3CA ,PPP6C, PTEN, PTPN11, RB1, RET, SMARCB1, SMO, SRC, STK11/LKB1, TACC1, TP53, VEGFR2/KDR*), covering genomic regions of approximately 246 kb.  Sequencing was performed on an Ion S5XL system (Thermo Fisher Scientific).

Briefly, we used 10 ng of gDNA to amplify 111 genes using the Ion AmpliSeq Library Kit2.0 (Thermofisher) according to the manufacturer’s manual (MAN0006735 rev 5.0). Emulsion PCR and chip loading were performed on The IonChef System (Thermo Fisher), according to the manufacturer’s instructions. gDNA sequencing was carried out on the ION S5XL System (Thermo Fisher) using Ion 540 Chip and ION 540 Chef Kit according to the manufacturer's instructions.

Data from the sequencing were initially processed using the Ion Torrent platform-specific software Torrent Suite Software™ (version 5.8) to generate sequence reads, alignment of the reads on the reference genome Hg19, trim adapter sequences, filter and remove poor signal-profile reads. The variant calling from the sequencing data was generated using the Variant Caller plugin. We applied some filters to that plugin to eliminate erroneous base calling: we set an average coverage depth >100, each variant coverage >20, a variant frequency on each sample >5, and a quality value >30. Filtered variants were visually examined using the Integrative Genomic Viewer (IGV) tool to test their level of quality and to confirm the variant presence on both the “+” and “-“ strands. The resulting variants were annotated using Ensemble Variant Effect Predictor pipeline, Ion Reporter™ analysis software, ClinVar database, COSMIC database and dbSNP database. The filtered variants were examined using the Integrative Genomic Viewer IGV tool. The coverage depth was always more than 500x and the reported mutations had a frequency of at least 5%. Matched normal DNA was used and where normal is not available, with MAF value greater than 0.01 in 1000 genomes combined population were considered as SNP and thus excluded we excluding variant with MAF >10^-6^ by using publically‐available or proprietary database of known polymorphisms (e.g., dbSNP, ExAC, 1000Genomes).

**APPENDIX**

**NGS gene panel used for the analysis**

| **Request_ID** | **Gene name** | **Chr** | **Chr_Start** | **Chr_End** | **Covered Bases** | **Target Bases** | **Missed Bases** |
| --- | --- | --- | --- | --- | --- | --- | --- |
| WG_IAD68410 | ***ARID1A*** | chr1 | 27022890 | 27024036 | 1092 | 1147 | 55 |
| WG_IAD68410 | ***ARID1A*** | chr1 | 27056137 | 27056359 | 223 | 223 | 0 |
| WG_IAD68410 | ***ARID1A*** | chr1 | 27057638 | 27058100 | 463 | 463 | 0 |
| WG_IAD68410 | ***ARID1A*** | chr1 | 27059162 | 27059288 | 127 | 127 | 0 |
| WG_IAD68410 | ***ARID1A*** | chr1 | 27087342 | 27087592 | 251 | 251 | 0 |
| WG_IAD68410 | ***ARID1A*** | chr1 | 27087870 | 27087969 | 100 | 100 | 0 |
| WG_IAD68410 | ***ARID1A*** | chr1 | 27088638 | 27088815 | 178 | 178 | 0 |
| WG_IAD68410 | ***ARID1A*** | chr1 | 27089459 | 27089781 | 323 | 323 | 0 |
| WG_IAD68410 | ***ARID1A*** | chr1 | 27092707 | 27092862 | 156 | 156 | 0 |
| WG_IAD68410 | ***ARID1A*** | chr1 | 27092943 | 27093062 | 120 | 120 | 0 |
| WG_IAD68410 | ***ARID1A*** | chr1 | 27094276 | 27094495 | 220 | 220 | 0 |
| WG_IAD68410 | ***ARID1A*** | chr1 | 27097605 | 27097822 | 218 | 218 | 0 |
| WG_IAD68410 | ***ARID1A*** | chr1 | 27098986 | 27099128 | 143 | 143 | 0 |
| WG_IAD68410 | ***ARID1A*** | chr1 | 27099298 | 27099483 | 186 | 186 | 0 |
| WG_IAD68410 | ***ARID1A*** | chr1 | 27099832 | 27099992 | 161 | 161 | 0 |
| WG_IAD68410 | ***ARID1A*** | chr1 | 27100066 | 27100213 | 148 | 148 | 0 |
| WG_IAD68410 | ***ARID1A*** | chr1 | 27100288 | 27100394 | 105 | 107 | 2 |
| WG_IAD68410 | ***ARID1A*** | chr1 | 27100815 | 27101716 | 902 | 902 | 0 |
| WG_IAD68410 | ***ARID1A*** | chr1 | 27102063 | 27102203 | 141 | 141 | 0 |
| WG_IAD68410 | ***ARID1A*** | chr1 | 27105509 | 27107252 | 1744 | 1744 | 0 |
| WG_IAD68410 | ***MTOR*** | chr1 | 11167537 | 11167562 | 26 | 26 | 0 |
| WG_IAD68410 | ***MTOR*** | chr1 | 11168233 | 11168348 | 116 | 116 | 0 |
| WG_IAD68410 | ***MTOR*** | chr1 | 11169342 | 11169432 | 91 | 91 | 0 |
| WG_IAD68410 | ***MTOR*** | chr1 | 11169701 | 11169791 | 91 | 91 | 0 |
| WG_IAD68410 | ***MTOR*** | chr1 | 11172904 | 11172979 | 76 | 76 | 0 |
| WG_IAD68410 | ***MTOR*** | chr1 | 11174370 | 11174515 | 146 | 146 | 0 |
| WG_IAD68410 | ***MTOR*** | chr1 | 11174865 | 11174949 | 85 | 85 | 0 |
| WG_IAD68410 | ***MTOR*** | chr1 | 11175448 | 11175530 | 83 | 83 | 0 |
| WG_IAD68410 | ***MTOR*** | chr1 | 11177056 | 11177148 | 93 | 93 | 0 |
| WG_IAD68410 | ***MTOR*** | chr1 | 11181298 | 11181430 | 133 | 133 | 0 |
| WG_IAD68410 | ***MTOR*** | chr1 | 11182031 | 11182188 | 158 | 158 | 0 |
| WG_IAD68410 | ***MTOR*** | chr1 | 11184550 | 11184695 | 146 | 146 | 0 |
| WG_IAD68410 | ***MTOR*** | chr1 | 11186674 | 11186858 | 185 | 185 | 0 |
| WG_IAD68410 | ***MTOR*** | chr1 | 11187062 | 11187206 | 145 | 145 | 0 |
| WG_IAD68410 | ***MTOR*** | chr1 | 11187676 | 11187868 | 193 | 193 | 0 |
| WG_IAD68410 | ***MTOR*** | chr1 | 11188056 | 11188188 | 133 | 133 | 0 |
| WG_IAD68410 | ***MTOR*** | chr1 | 11188506 | 11188614 | 109 | 109 | 0 |
| WG_IAD68410 | ***MTOR*** | chr1 | 11188907 | 11189013 | 107 | 107 | 0 |
| WG_IAD68410 | ***MTOR*** | chr1 | 11189790 | 11189900 | 111 | 111 | 0 |
| WG_IAD68410 | ***MTOR*** | chr1 | 11190581 | 11190839 | 259 | 259 | 0 |
| WG_IAD68410 | ***MTOR*** | chr1 | 11193132 | 11193259 | 128 | 128 | 0 |
| WG_IAD68410 | ***MTOR*** | chr1 | 11194403 | 11194528 | 126 | 126 | 0 |
| WG_IAD68410 | ***MTOR*** | chr1 | 11199356 | 11199497 | 142 | 142 | 0 |
| WG_IAD68410 | ***MTOR*** | chr1 | 11199585 | 11199720 | 136 | 136 | 0 |
| WG_IAD68410 | ***MTOR*** | chr1 | 11204700 | 11204817 | 118 | 118 | 0 |
| WG_IAD68410 | ***MTOR*** | chr1 | 11205020 | 11205107 | 88 | 88 | 0 |
| WG_IAD68410 | ***MTOR*** | chr1 | 11206728 | 11206853 | 126 | 126 | 0 |
| WG_IAD68410 | ***MTOR*** | chr1 | 11210178 | 11210288 | 111 | 111 | 0 |
| WG_IAD68410 | ***MTOR*** | chr1 | 11217204 | 11217353 | 150 | 150 | 0 |
| WG_IAD68410 | ***MTOR*** | chr1 | 11227494 | 11227579 | 86 | 86 | 0 |
| WG_IAD68410 | ***MTOR*** | chr1 | 11259310 | 11259465 | 156 | 156 | 0 |
| WG_IAD68410 | ***MTOR*** | chr1 | 11259593 | 11259765 | 173 | 173 | 0 |
| WG_IAD68410 | ***MTOR*** | chr1 | 11264613 | 11264765 | 153 | 153 | 0 |
| WG_IAD68410 | ***MTOR*** | chr1 | 11269364 | 11269520 | 157 | 157 | 0 |
| WG_IAD68410 | ***MTOR*** | chr1 | 11270866 | 11270968 | 103 | 103 | 0 |
| WG_IAD68410 | ***MTOR*** | chr1 | 11272364 | 11272536 | 173 | 173 | 0 |
| WG_IAD68410 | ***MTOR*** | chr1 | 11272848 | 11272970 | 123 | 123 | 0 |
| WG_IAD68410 | ***MTOR*** | chr1 | 11273451 | 11273628 | 178 | 178 | 0 |
| WG_IAD68410 | ***MTOR*** | chr1 | 11276200 | 11276296 | 97 | 97 | 0 |
| WG_IAD68410 | ***MTOR*** | chr1 | 11288720 | 11288980 | 261 | 261 | 0 |
| WG_IAD68410 | ***MTOR*** | chr1 | 11290977 | 11291116 | 140 | 140 | 0 |
| WG_IAD68410 | ***MTOR*** | chr1 | 11291352 | 11291496 | 145 | 145 | 0 |
| WG_IAD68410 | ***MTOR*** | chr1 | 11292488 | 11292590 | 103 | 103 | 0 |
| WG_IAD68410 | ***MTOR*** | chr1 | 11293450 | 11293549 | 100 | 100 | 0 |
| WG_IAD68410 | ***MTOR*** | chr1 | 11294195 | 11294327 | 133 | 133 | 0 |
| WG_IAD68410 | ***MTOR*** | chr1 | 11297895 | 11298110 | 216 | 216 | 0 |
| WG_IAD68410 | ***MTOR*** | chr1 | 11298454 | 11298679 | 226 | 226 | 0 |
| WG_IAD68410 | ***MTOR*** | chr1 | 11300355 | 11300609 | 255 | 255 | 0 |
| WG_IAD68410 | ***MTOR*** | chr1 | 11301605 | 11301743 | 139 | 139 | 0 |
| WG_IAD68410 | ***MTOR*** | chr1 | 11303166 | 11303362 | 197 | 197 | 0 |
| WG_IAD68410 | ***MTOR*** | chr1 | 11307677 | 11307795 | 119 | 119 | 0 |
| WG_IAD68410 | ***MTOR*** | chr1 | 11307871 | 11308156 | 286 | 286 | 0 |
| WG_IAD68410 | ***MTOR*** | chr1 | 11313891 | 11314035 | 145 | 145 | 0 |
| WG_IAD68410 | ***MTOR*** | chr1 | 11316044 | 11316254 | 211 | 211 | 0 |
| WG_IAD68410 | ***MTOR*** | chr1 | 11316985 | 11317227 | 243 | 243 | 0 |
| WG_IAD68410 | ***MTOR*** | chr1 | 11318537 | 11318655 | 119 | 119 | 0 |
| WG_IAD68410 | ***MTOR*** | chr1 | 11319300 | 11319471 | 172 | 172 | 0 |
| WG_IAD68410 | ***RIT1*** | chr1 | 155870174 | 155870414 | 241 | 241 | 0 |
| WG_IAD68410 | ***RIT1*** | chr1 | 155874097 | 155874298 | 202 | 202 | 0 |
| WG_IAD68410 | ***RIT1*** | chr1 | 155874517 | 155874600 | 84 | 84 | 0 |
| WG_IAD68410 | ***RIT1*** | chr1 | 155880236 | 155880302 | 67 | 67 | 0 |
| WG_IAD68410 | ***RIT1*** | chr1 | 155880442 | 155880600 | 159 | 159 | 0 |
| WG_IAD68410 | ***RIT1*** | chr1 | 155880664 | 155880681 | 18 | 18 | 0 |
| WG_IAD68410 | ***RUNX3*** | chr1 | 25228608 | 25229162 | 555 | 555 | 0 |
| WG_IAD68410 | ***RUNX3*** | chr1 | 25233745 | 25233913 | 169 | 169 | 0 |
| WG_IAD68410 | ***RUNX3*** | chr1 | 25245726 | 25245840 | 115 | 115 | 0 |
| WG_IAD68410 | ***RUNX3*** | chr1 | 25254060 | 25254226 | 167 | 167 | 0 |
| WG_IAD68410 | ***RUNX3*** | chr1 | 25256073 | 25256364 | 292 | 292 | 0 |
| WG_IAD68410 | ***RUNX3*** | chr1 | 25291000 | 25291067 | 68 | 68 | 0 |
| WG_IAD68410 | ***SDHB*** | chr1 | 17345371 | 17345458 | 88 | 88 | 0 |
| WG_IAD68410 | ***SDHB*** | chr1 | 17349098 | 17349230 | 133 | 133 | 0 |
| WG_IAD68410 | ***SDHB*** | chr1 | 17350463 | 17350574 | 112 | 112 | 0 |
| WG_IAD68410 | ***SDHB*** | chr1 | 17354239 | 17354365 | 127 | 127 | 0 |
| WG_IAD68410 | ***SDHB*** | chr1 | 17355090 | 17355236 | 147 | 147 | 0 |
| WG_IAD68410 | ***SDHB*** | chr1 | 17359550 | 17359645 | 96 | 96 | 0 |
| WG_IAD68410 | ***SDHB*** | chr1 | 17371251 | 17371388 | 138 | 138 | 0 |
| WG_IAD68410 | ***SDHB*** | chr1 | 17380438 | 17380519 | 82 | 82 | 0 |
| WG_IAD68410 | ***SDHC*** | chr1 | 161284191 | 161284220 | 30 | 30 | 0 |
| WG_IAD68410 | ***SDHC*** | chr1 | 161293399 | 161293465 | 51 | 67 | 16 |
| WG_IAD68410 | ***SDHC*** | chr1 | 161298181 | 161298292 | 112 | 112 | 0 |
| WG_IAD68410 | ***SDHC*** | chr1 | 161310379 | 161310450 | 72 | 72 | 0 |
| WG_IAD68410 | ***SDHC*** | chr1 | 161326462 | 161326635 | 174 | 174 | 0 |
| WG_IAD68410 | ***SDHC*** | chr1 | 161332114 | 161332335 | 154 | 222 | 68 |
| WG_IAD68410 | ***MPL_10*** | chr1 | 43814934 | 43815030 | 97 | 97 | 0 |
| WG_IAD68410 | ***NRAS_4*** | chr1 | 115252190 | 115252349 | 160 | 160 | 0 |
| WG_IAD68410 | ***NRAS_3*** | chr1 | 115256421 | 115256599 | 179 | 179 | 0 |
| WG_IAD68410 | ***NRAS_2*** | chr1 | 115258671 | 115258798 | 128 | 128 | 0 |
| WG_IAD68410 | ***DDR2_10*** | chr1 | 162740092 | 162740302 | 211 | 211 | 0 |
| WG_IAD68410 | ***DDR2_11*** | chr1 | 162741814 | 162742037 | 224 | 224 | 0 |
| WG_IAD68410 | ***DDR2_12*** | chr1 | 162743259 | 162743386 | 128 | 128 | 0 |
| WG_IAD68410 | ***DDR2_13*** | chr1 | 162745442 | 162745633 | 192 | 192 | 0 |
| WG_IAD68410 | ***DDR2_14*** | chr1 | 162745926 | 162746160 | 235 | 235 | 0 |
| WG_IAD68410 | ***DDR2_15*** | chr1 | 162748370 | 162748519 | 150 | 150 | 0 |
| WG_IAD68410 | ***DDR2_16*** | chr1 | 162749902 | 162750022 | 121 | 121 | 0 |
| WG_IAD68410 | ***MSH2*** | chr2 | 47630326 | 47630546 | 221 | 221 | 0 |
| WG_IAD68410 | ***MSH2*** | chr2 | 47635535 | 47635699 | 165 | 165 | 0 |
| WG_IAD68410 | ***MSH2*** | chr2 | 47637228 | 47637516 | 289 | 289 | 0 |
| WG_IAD68410 | ***MSH2*** | chr2 | 47639548 | 47639704 | 157 | 157 | 0 |
| WG_IAD68410 | ***MSH2*** | chr2 | 47641403 | 47641562 | 136 | 160 | 24 |
| WG_IAD68410 | ***MSH2*** | chr2 | 47643430 | 47643573 | 144 | 144 | 0 |
| WG_IAD68410 | ***MSH2*** | chr2 | 47656876 | 47657085 | 210 | 210 | 0 |
| WG_IAD68410 | ***MSH2*** | chr2 | 47672682 | 47672801 | 120 | 120 | 0 |
| WG_IAD68410 | ***MSH2*** | chr2 | 47690165 | 47690298 | 134 | 134 | 0 |
| WG_IAD68410 | ***MSH2*** | chr2 | 47693792 | 47693952 | 161 | 161 | 0 |
| WG_IAD68410 | ***MSH2*** | chr2 | 47698099 | 47698206 | 108 | 108 | 0 |
| WG_IAD68410 | ***MSH2*** | chr2 | 47702159 | 47702414 | 256 | 256 | 0 |
| WG_IAD68410 | ***MSH2*** | chr2 | 47703501 | 47703715 | 215 | 215 | 0 |
| WG_IAD68410 | ***MSH2*** | chr2 | 47705406 | 47705663 | 258 | 258 | 0 |
| WG_IAD68410 | ***MSH2*** | chr2 | 47707830 | 47708015 | 186 | 186 | 0 |
| WG_IAD68410 | ***MSH2*** | chr2 | 47709913 | 47710093 | 181 | 181 | 0 |
| WG_IAD68410 | ***MSH6*** | chr2 | 48010368 | 48010637 | 270 | 270 | 0 |
| WG_IAD68410 | ***MSH6*** | chr2 | 48018061 | 48018267 | 207 | 207 | 0 |
| WG_IAD68410 | ***MSH6*** | chr2 | 48023028 | 48023207 | 180 | 180 | 0 |
| WG_IAD68410 | ***MSH6*** | chr2 | 48025745 | 48028299 | 2555 | 2555 | 0 |
| WG_IAD68410 | ***MSH6*** | chr2 | 48030554 | 48030829 | 276 | 276 | 0 |
| WG_IAD68410 | ***MSH6*** | chr2 | 48032044 | 48032171 | 128 | 128 | 0 |
| WG_IAD68410 | ***MSH6*** | chr2 | 48032752 | 48032851 | 100 | 100 | 0 |
| WG_IAD68410 | ***MSH6*** | chr2 | 48033338 | 48033502 | 165 | 165 | 0 |
| WG_IAD68410 | ***MSH6*** | chr2 | 48033586 | 48033795 | 210 | 210 | 0 |
| WG_IAD68410 | ***MSH6*** | chr2 | 48033913 | 48034004 | 92 | 92 | 0 |
| WG_IAD68410 | ***ALK_25*** | chr2 | 29432652 | 29432744 | 93 | 93 | 0 |
| WG_IAD68410 | ***ALK_24*** | chr2 | 29436850 | 29436947 | 98 | 98 | 0 |
| WG_IAD68410 | ***ALK_23*** | chr2 | 29443572 | 29443701 | 130 | 130 | 0 |
| WG_IAD68410 | ***ALK_22*** | chr2 | 29445210 | 29445274 | 65 | 65 | 0 |
| WG_IAD68410 | ***ALK_21*** | chr2 | 29445383 | 29445473 | 91 | 91 | 0 |
| WG_IAD68410 | ***IDH1_4*** | chr2 | 209113093 | 209113384 | 292 | 292 | 0 |
| WG_IAD68410 | ***HER4_23*** | chr2 | 212288880 | 212289026 | 147 | 147 | 0 |
| WG_IAD68410 | ***HER4_15*** | chr2 | 212530048 | 212530202 | 155 | 155 | 0 |
| WG_IAD68410 | ***HER4_9*** | chr2 | 212576775 | 212576901 | 127 | 127 | 0 |
| WG_IAD68410 | ***HER4_8*** | chr2 | 212578260 | 212578373 | 114 | 114 | 0 |
| WG_IAD68410 | ***HER4_7*** | chr2 | 212587118 | 212587259 | 142 | 142 | 0 |
| WG_IAD68410 | ***HER4_6*** | chr2 | 212589801 | 212589919 | 119 | 119 | 0 |
| WG_IAD68410 | ***HER4_4*** | chr2 | 212652750 | 212652884 | 135 | 135 | 0 |
| WG_IAD68410 | ***HER4_3*** | chr2 | 212812155 | 212812341 | 187 | 187 | 0 |
| WG_IAD68410 | ***BAP1*** | chr3 | 52436299 | 52436442 | 144 | 144 | 0 |
| WG_IAD68410 | ***BAP1*** | chr3 | 52436613 | 52436695 | 83 | 83 | 0 |
| WG_IAD68410 | ***BAP1*** | chr3 | 52436790 | 52436892 | 103 | 103 | 0 |
| WG_IAD68410 | ***BAP1*** | chr3 | 52437149 | 52437319 | 171 | 171 | 0 |
| WG_IAD68410 | ***BAP1*** | chr3 | 52437427 | 52437915 | 489 | 489 | 0 |
| WG_IAD68410 | ***BAP1*** | chr3 | 52438464 | 52438607 | 144 | 144 | 0 |
| WG_IAD68410 | ***BAP1*** | chr3 | 52439121 | 52439315 | 195 | 195 | 0 |
| WG_IAD68410 | ***BAP1*** | chr3 | 52439776 | 52439933 | 158 | 158 | 0 |
| WG_IAD68410 | ***BAP1*** | chr3 | 52440264 | 52440397 | 134 | 134 | 0 |
| WG_IAD68410 | ***BAP1*** | chr3 | 52440840 | 52440928 | 89 | 89 | 0 |
| WG_IAD68410 | ***BAP1*** | chr3 | 52441185 | 52441337 | 153 | 153 | 0 |
| WG_IAD68410 | ***BAP1*** | chr3 | 52441410 | 52441481 | 72 | 72 | 0 |
| WG_IAD68410 | ***BAP1*** | chr3 | 52441969 | 52442098 | 130 | 130 | 0 |
| WG_IAD68410 | ***BAP1*** | chr3 | 52442485 | 52442627 | 143 | 143 | 0 |
| WG_IAD68410 | ***BAP1*** | chr3 | 52443565 | 52443629 | 65 | 65 | 0 |
| WG_IAD68410 | ***BAP1*** | chr3 | 52443725 | 52443764 | 40 | 40 | 0 |
| WG_IAD68410 | ***BAP1*** | chr3 | 52443853 | 52443899 | 47 | 47 | 0 |
| WG_IAD68410 | ***FHIT*** | chr3 | 59737947 | 59738052 | 106 | 106 | 0 |
| WG_IAD68410 | ***FHIT*** | chr3 | 59908067 | 59908145 | 79 | 79 | 0 |
| WG_IAD68410 | ***FHIT*** | chr3 | 59997092 | 59997131 | 40 | 40 | 0 |
| WG_IAD68410 | ***FHIT*** | chr3 | 59999728 | 59999883 | 156 | 156 | 0 |
| WG_IAD68410 | ***FHIT*** | chr3 | 60522588 | 60522700 | 113 | 113 | 0 |
| WG_IAD68410 | ***PBRM1*** | chr3 | 52582074 | 52582256 | 183 | 183 | 0 |
| WG_IAD68410 | ***PBRM1*** | chr3 | 52584432 | 52584658 | 227 | 227 | 0 |
| WG_IAD68410 | ***PBRM1*** | chr3 | 52584758 | 52584838 | 81 | 81 | 0 |
| WG_IAD68410 | ***PBRM1*** | chr3 | 52588735 | 52588900 | 166 | 166 | 0 |
| WG_IAD68410 | ***PBRM1*** | chr3 | 52595778 | 52595989 | 212 | 212 | 0 |
| WG_IAD68410 | ***PBRM1*** | chr3 | 52597375 | 52597514 | 140 | 140 | 0 |
| WG_IAD68410 | ***PBRM1*** | chr3 | 52598061 | 52598254 | 194 | 194 | 0 |
| WG_IAD68410 | ***PBRM1*** | chr3 | 52610552 | 52610719 | 168 | 168 | 0 |
| WG_IAD68410 | ***PBRM1*** | chr3 | 52613065 | 52613220 | 156 | 156 | 0 |
| WG_IAD68410 | ***PBRM1*** | chr3 | 52620436 | 52620709 | 274 | 274 | 0 |
| WG_IAD68410 | ***PBRM1*** | chr3 | 52621364 | 52621456 | 93 | 93 | 0 |
| WG_IAD68410 | ***PBRM1*** | chr3 | 52623081 | 52623276 | 196 | 196 | 0 |
| WG_IAD68410 | ***PBRM1*** | chr3 | 52637532 | 52637753 | 222 | 222 | 0 |
| WG_IAD68410 | ***PBRM1*** | chr3 | 52643324 | 52643976 | 653 | 653 | 0 |
| WG_IAD68410 | ***PBRM1*** | chr3 | 52649362 | 52649477 | 116 | 116 | 0 |
| WG_IAD68410 | ***PBRM1*** | chr3 | 52651273 | 52651559 | 287 | 287 | 0 |
| WG_IAD68410 | ***PBRM1*** | chr3 | 52661284 | 52661391 | 108 | 108 | 0 |
| WG_IAD68410 | ***PBRM1*** | chr3 | 52662905 | 52663056 | 152 | 152 | 0 |
| WG_IAD68410 | ***PBRM1*** | chr3 | 52668613 | 52668836 | 224 | 224 | 0 |
| WG_IAD68410 | ***PBRM1*** | chr3 | 52675965 | 52676066 | 102 | 102 | 0 |
| WG_IAD68410 | ***PBRM1*** | chr3 | 52677259 | 52677364 | 106 | 106 | 0 |
| WG_IAD68410 | ***PBRM1*** | chr3 | 52678715 | 52678810 | 96 | 96 | 0 |
| G_IAD68410 | ***PBRM1*** | chr3 | 52682355 | 52682463 | 109 | 109 | 0 |
| WG_IAD68410 | ***PBRM1*** | chr3 | 52685753 | 52685831 | 79 | 79 | 0 |
| WG_IAD68410 | ***PBRM1*** | chr3 | 52692210 | 52692336 | 127 | 127 | 0 |
| WG_IAD68410 | ***PBRM1*** | chr3 | 52696144 | 52696297 | 154 | 154 | 0 |
| WG_IAD68410 | ***PBRM1*** | chr3 | 52702509 | 52702666 | 158 | 158 | 0 |
| WG_IAD68410 | ***PBRM1*** | chr3 | 52712511 | 52712618 | 108 | 108 | 0 |
| WG_IAD68410 | ***PBRM1*** | chr3 | 52713585 | 52713732 | 148 | 148 | 0 |
| WG_IAD68410 | ***SETD2*** | chr3 | 47058578 | 47058749 | 172 | 172 | 0 |
| WG_IAD68410 | ***SETD2*** | chr3 | 47059123 | 47059234 | 112 | 112 | 0 |
| WG_IAD68410 | ***SETD2*** | chr3 | 47061245 | 47061335 | 91 | 91 | 0 |
| WG_IAD68410 | ***SETD2*** | chr3 | 47079151 | 47079272 | 122 | 122 | 0 |
| WG_IAD68410 | ***SETD2*** | chr3 | 47084046 | 47084195 | 150 | 150 | 0 |
| WG_IAD68410 | ***SETD2*** | chr3 | 47087972 | 47088116 | 145 | 145 | 0 |
| WG_IAD68410 | ***SETD2*** | chr3 | 47098306 | 47098985 | 680 | 680 | 0 |
| WG_IAD68410 | ***SETD2*** | chr3 | 47103648 | 47103841 | 194 | 194 | 0 |
| WG_IAD68410 | ***SETD2*** | chr3 | 47108555 | 47108613 | 59 | 59 | 0 |
| WG_IAD68410 | ***SETD2*** | chr3 | 47125205 | 47125877 | 673 | 673 | 0 |
| WG_IAD68410 | ***SETD2*** | chr3 | 47127680 | 47127809 | 130 | 130 | 0 |
| WG_IAD68410 | ***SETD2*** | chr3 | 47129598 | 47129742 | 145 | 145 | 0 |
| WG_IAD68410 | ***SETD2*** | chr3 | 47139440 | 47139576 | 137 | 137 | 0 |
| WG_IAD68410 | ***SETD2*** | chr3 | 47142943 | 47143050 | 108 | 108 | 0 |
| WG_IAD68410 | ***SETD2*** | chr3 | 47144831 | 47144918 | 88 | 88 | 0 |
| WG_IAD68410 | ***SETD2*** | chr3 | 47147482 | 47147615 | 134 | 134 | 0 |
| WG_IAD68410 | ***SETD2*** | chr3 | 47155361 | 47155499 | 119 | 139 | 20 |
| WG_IAD68410 | ***SETD2*** | chr3 | 47158108 | 47158249 | 142 | 142 | 0 |
| WG_IAD68410 | ***SETD2*** | chr3 | 47161667 | 47166043 | 4377 | 4377 | 0 |
| WG_IAD68410 | ***SETD2*** | chr3 | 47168133 | 47168158 | 26 | 26 | 0 |
| WG_IAD68410 | ***SETD2*** | chr3 | 47205339 | 47205419 | 81 | 81 | 0 |
| WG_IAD68410 | ***TGFBR2*** | chr3 | 30648371 | 30648474 | 104 | 104 | 0 |
| WG_IAD68410 | ***TGFBR2*** | chr3 | 30664686 | 30664770 | 85 | 85 | 0 |
| WG_IAD68410 | ***TGFBR2*** | chr3 | 30686234 | 30686412 | 179 | 179 | 0 |
| WG_IAD68410 | ***TGFBR2*** | chr3 | 30691757 | 30691957 | 201 | 201 | 0 |
| WG_IAD68410 | ***TGFBR2*** | chr3 | 30713125 | 30713934 | 810 | 810 | 0 |
| WG_IAD68410 | ***TGFBR2*** | chr3 | 30715592 | 30715743 | 152 | 152 | 0 |
| WG_IAD68410 | ***TGFBR2*** | chr3 | 30729871 | 30730008 | 138 | 138 | 0 |
| WG_IAD68410 | ***TGFBR2*** | chr3 | 30732907 | 30733096 | 190 | 190 | 0 |
| WG_IAD68410 | ***TXNRD3*** | chr3 | 126326743 | 126326821 | 79 | 79 | 0 |
| WG_IAD68410 | ***TXNRD3*** | chr3 | 126327337 | 126327481 | 145 | 145 | 0 |
| WG_IAD68410 | ***TXNRD3*** | chr3 | 126329875 | 126329980 | 106 | 106 | 0 |
| WG_IAD68410 | ***TXNRD3*** | chr3 | 126334193 | 126334310 | 118 | 118 | 0 |
| WG_IAD68410 | ***TXNRD3*** | chr3 | 126340580 | 126340746 | 167 | 167 | 0 |
| WG_IAD68410 | ***TXNRD3*** | chr3 | 126341302 | 126341388 | 87 | 87 | 0 |
| WG_IAD68410 | ***TXNRD3*** | chr3 | 126348217 | 126348319 | 103 | 103 | 0 |
| WG_IAD68410 | ***TXNRD3*** | chr3 | 126349550 | 126349785 | 236 | 236 | 0 |
| WG_IAD68410 | ***TXNRD3*** | chr3 | 126350602 | 126350727 | 126 | 126 | 0 |
| WG_IAD68410 | ***TXNRD3*** | chr3 | 126352747 | 126352899 | 153 | 153 | 0 |
| WG_IAD68410 | ***TXNRD3*** | chr3 | 126360870 | 126360999 | 130 | 130 | 0 |
| WG_IAD68410 | ***TXNRD3*** | chr3 | 126362819 | 126362901 | 83 | 83 | 0 |
| WG_IAD68410 | ***TXNRD3*** | chr3 | 126363135 | 126363249 | 115 | 115 | 0 |
| WG_IAD68410 | ***TXNRD3*** | chr3 | 126364949 | 126365068 | 120 | 120 | 0 |
| WG_IAD68410 | ***TXNRD3*** | chr3 | 126366074 | 126366144 | 71 | 71 | 0 |
| WG_IAD68410 | ***TXNRD3*** | chr3 | 126373586 | 126373838 | 253 | 253 | 0 |
| WG_IAD68410 | ***VHL*** | chr3 | 10183527 | 10183876 | 350 | 350 | 0 |
| WG_IAD68410 | ***VHL*** | chr3 | 10188193 | 10188325 | 133 | 133 | 0 |
| WG_IAD68410 | ***VHL*** | chr3 | 10191466 | 10191654 | 189 | 189 | 0 |
| WG_IAD68410 | ***MLH1_12*** | chr3 | 37067128 | 37067498 | 354 | 371 | 17 |
| WG_IAD68410 | ***CTNNB1_3*** | chr3 | 41266017 | 41266244 | 228 | 228 | 0 |
| WG_IAD68410 | ***PIK3CA_1*** | chr3 | 178916612 | 178916965 | 354 | 354 | 0 |
| WG_IAD68410 | ***PIK3CA_2*** | chr3 | 178917478 | 178917687 | 210 | 210 | 0 |
| WG_IAD68410 | ***PIK3CA_4*** | chr3 | 178921332 | 178921577 | 246 | 246 | 0 |
| WG_IAD68410 | ***PIK3CA_6*** | chr3 | 178927383 | 178927488 | 106 | 106 | 0 |
| WG_IAD68410 | ***PIK3CA_7*** | chr3 | 178927974 | 178928126 | 153 | 153 | 0 |
| WG_IAD68410 | ***PIK3CA_9*** | chr3 | 178935998 | 178936122 | 106 | 125 | 19 |
| WG_IAD68410 | ***PIK3CA_13*** | chr3 | 178938774 | 178938945 | 172 | 172 | 0 |
| WG_IAD68410 | ***PIK3CA_18*** | chr3 | 178947792 | 178947909 | 118 | 118 | 0 |
| WG_IAD68410 | ***PIK3CA_20*** | chr3 | 178951882 | 178952161 | 280 | 280 | 0 |
| WG_IAD68410 | ***FGFR3_7*** | chr4 | 1803562 | 1803752 | 191 | 191 | 0 |
| WG_IAD68410 | ***FGFR3_9*** | chr4 | 1806057 | 1806247 | 191 | 191 | 0 |
| WG_IAD68410 | ***FGFR3_14*** | chr4 | 1807778 | 1807900 | 123 | 123 | 0 |
| WG_IAD68410 | ***FGFR3_16*** | chr4 | 1808273 | 1808410 | 138 | 138 | 0 |
| WG_IAD68410 | ***FGFR3_18*** | chr4 | 1808843 | 1809022 | 180 | 180 | 0 |
| WG_IAD68410 | ***PDGFRA_10*** | chr4 | 55139704 | 55139897 | 194 | 194 | 0 |
| WG_IAD68410 | ***PDGFRA_12*** | chr4 | 55141008 | 55141140 | 133 | 133 | 0 |
| WG_IAD68410 | ***PDGFRA_13*** | chr4 | 55143555 | 55143659 | 105 | 105 | 0 |
| WG_IAD68410 | ***PDGFRA_14*** | chr4 | 55144063 | 55144173 | 111 | 111 | 0 |
| WG_IAD68410 | ***PDGFRA_15*** | chr4 | 55144529 | 55144682 | 154 | 154 | 0 |
| WG_IAD68410 | ***PDGFRA_18*** | chr4 | 55152038 | 55152100 | 63 | 63 | 0 |
| WG_IAD68410 | ***KIT_2*** | chr4 | 55561678 | 55561947 | 270 | 270 | 0 |
| WG_IAD68410 | ***KIT_9*** | chr4 | 55592023 | 55592216 | 194 | 194 | 0 |
| WG_IAD68410 | ***KIT_10*** | chr4 | 55593384 | 55593490 | 107 | 107 | 0 |
| WG_IAD68410 | ***KIT_11*** | chr4 | 55593585 | 55593710 | 111 | 126 | 15 |
| WG_IAD68410 | ***KIT_13*** | chr4 | 55594177 | 55594287 | 111 | 111 | 0 |
| WG_IAD68410 | ***KIT_14*** | chr4 | 55595501 | 55595651 | 151 | 151 | 0 |
| WG_IAD68410 | ***KIT_15*** | chr4 | 55597494 | 55597585 | 92 | 92 | 0 |
| WG_IAD68410 | ***KIT_17*** | chr4 | 55599236 | 55599358 | 123 | 123 | 0 |
| WG_IAD68410 | ***KIT_18*** | chr4 | 55602664 | 55602775 | 112 | 112 | 0 |
| WG_IAD68410 | ***KDR_30*** | chr4 | 55946091 | 55946330 | 240 | 240 | 0 |
| WG_IAD68410 | ***KDR_27*** | chr4 | 55953774 | 55953925 | 152 | 152 | 0 |
| WG_IAD68410 | ***KDR_26*** | chr4 | 55955035 | 55955140 | 106 | 106 | 0 |
| WG_IAD68410 | ***KDR_21*** | chr4 | 55960969 | 55961122 | 154 | 154 | 0 |
| WG_IAD68410 | ***KDR_19*** | chr4 | 55962396 | 55962509 | 114 | 114 | 0 |
| WG_IAD68410 | ***KDR_11*** | chr4 | 55972854 | 55972977 | 124 | 124 | 0 |
| WG_IAD68410 | ***KDR_7*** | chr4 | 55979471 | 55979648 | 178 | 178 | 0 |
| WG_IAD68410 | ***KDR_6*** | chr4 | 55980293 | 55980432 | 140 | 140 | 0 |
| WG_IAD68410 | ***FBXW7_11*** | chr4 | 153245336 | 153245546 | 211 | 211 | 0 |
| WG_IAD68410 | ***FBXW7_10*** | chr4 | 153247158 | 153247383 | 226 | 226 | 0 |
| WG_IAD68410 | ***FBXW7_9*** | chr4 | 153249360 | 153249541 | 182 | 182 | 0 |
| WG_IAD68410 | ***FBXW7_8*** | chr4 | 153250824 | 153250937 | 114 | 114 | 0 |
| WG_IAD68410 | ***FBXW7_5*** | chr4 | 153258954 | 153259088 | 135 | 135 | 0 |
| WG_IAD68410 | ***FGFR4*** | chr5 | 176516599 | 176516699 | 101 | 101 | 0 |
| WG_IAD68410 | ***FGFR4*** | chr5 | 176517386 | 176517659 | 274 | 274 | 0 |
| WG_IAD68410 | ***FGFR4*** | chr5 | 176517741 | 176517831 | 91 | 91 | 0 |
| WG_IAD68410 | ***FGFR4*** | chr5 | 176517934 | 176518110 | 177 | 177 | 0 |
| WG_IAD68410 | ***FGFR4*** | chr5 | 176518681 | 176518814 | 134 | 134 | 0 |
| WG_IAD68410 | ***FGFR4*** | chr5 | 176519317 | 176519517 | 201 | 201 | 0 |
| WG_IAD68410 | ***FGFR4*** | chr5 | 176519642 | 176519790 | 149 | 149 | 0 |
| WG_IAD68410 | ***FGFR4*** | chr5 | 176520134 | 176520337 | 204 | 204 | 0 |
| WG_IAD68410 | ***FGFR4*** | chr5 | 176520328 | 176520557 | 230 | 230 | 0 |
| WG_IAD68410 | ***FGFR4*** | chr5 | 176520650 | 176520781 | 132 | 132 | 0 |
| WG_IAD68410 | ***FGFR4*** | chr5 | 176522326 | 176522446 | 121 | 121 | 0 |
| WG_IAD68410 | ***FGFR4*** | chr5 | 176522529 | 176522729 | 201 | 201 | 0 |
| WG_IAD68410 | ***FGFR4*** | chr5 | 176523053 | 176523185 | 133 | 133 | 0 |
| WG_IAD68410 | ***FGFR4*** | chr5 | 176523283 | 176523363 | 81 | 81 | 0 |
| WG_IAD68410 | ***FGFR4*** | chr5 | 176523600 | 176523747 | 148 | 148 | 0 |
| WG_IAD68410 | ***FGFR4*** | chr5 | 176524288 | 176524403 | 116 | 116 | 0 |
| WG_IAD68410 | ***FGFR4*** | chr5 | 176524523 | 176524682 | 160 | 160 | 0 |
| WG_IAD68410 | ***FLT4*** | chr5 | 180030187 | 180030395 | 209 | 209 | 0 |
| WG_IAD68410 | ***FLT4*** | chr5 | 180035276 | 180035289 | 12 | 14 | 2 |
| WG_IAD68410 | ***FLT4*** | chr5 | 180035963 | 180036058 | 96 | 96 | 0 |
| WG_IAD68410 | ***FLT4*** | chr5 | 180036900 | 180037030 | 131 | 131 | 0 |
| WG_IAD68410 | ***FLT4*** | chr5 | 180038326 | 180038484 | 159 | 159 | 0 |
| WG_IAD68410 | ***FLT4*** | chr5 | 180039501 | 180039616 | 116 | 116 | 0 |
| WG_IAD68410 | ***FLT4*** | chr5 | 180040006 | 180040115 | 110 | 110 | 0 |
| WG_IAD68410 | ***FLT4*** | chr5 | 180041063 | 180041184 | 122 | 122 | 0 |
| WG_IAD68410 | ***FLT4*** | chr5 | 180043362 | 180043494 | 133 | 133 | 0 |
| WG_IAD68410 | ***FLT4*** | chr5 | 180043895 | 180043999 | 105 | 105 | 0 |
| WG_IAD68410 | ***FLT4*** | chr5 | 180045765 | 180045925 | 161 | 161 | 0 |
| WG_IAD68410 | ***FLT4*** | chr5 | 180046016 | 180046114 | 83 | 99 | 16 |
| WG_IAD68410 | ***FLT4*** | chr5 | 180046248 | 180046371 | 124 | 124 | 0 |
| WG_IAD68410 | ***FLT4*** | chr5 | 180046660 | 180046774 | 115 | 115 | 0 |
| WG_IAD68410 | ***FLT4*** | chr5 | 180047168 | 180047313 | 146 | 146 | 0 |
| WG_IAD68410 | ***FLT4*** | chr5 | 180047604 | 180047720 | 117 | 117 | 0 |
| WG_IAD68410 | ***FLT4*** | chr5 | 180047871 | 180048012 | 142 | 142 | 0 |
| WG_IAD68410 | ***FLT4*** | chr5 | 180048101 | 180048257 | 157 | 157 | 0 |
| WG_IAD68410 | ***FLT4*** | chr5 | 180048537 | 180048909 | 373 | 373 | 0 |
| WG_IAD68410 | ***FLT4*** | chr5 | 180049726 | 180049844 | 119 | 119 | 0 |
| WG_IAD68410 | ***FLT4*** | chr5 | 180050930 | 180051066 | 137 | 137 | 0 |
| WG_IAD68410 | ***FLT4*** | chr5 | 180052864 | 180053036 | 173 | 173 | 0 |
| WG_IAD68410 | ***FLT4*** | chr5 | 180053106 | 180053270 | 165 | 165 | 0 |
| WG_IAD68410 | ***FLT4*** | chr5 | 180055877 | 180056004 | 128 | 128 | 0 |
| WG_IAD68410 | ***FLT4*** | chr5 | 180056254 | 180056432 | 179 | 179 | 0 |
| WG_IAD68410 | ***FLT4*** | chr5 | 180056691 | 180056840 | 150 | 150 | 0 |
| WG_IAD68410 | ***FLT4*** | chr5 | 180056938 | 180057110 | 173 | 173 | 0 |
| WG_IAD68410 | ***FLT4*** | chr5 | 180057220 | 180057342 | 123 | 123 | 0 |
| WG_IAD68410 | ***FLT4*** | chr5 | 180057550 | 180057804 | 255 | 255 | 0 |
| WG_IAD68410 | ***FLT4*** | chr5 | 180058677 | 180058783 | 107 | 107 | 0 |
| WG_IAD68410 | ***FLT4*** | chr5 | 180076483 | 180076550 | 68 | 68 | 0 |
| WG_IAD68410 | ***PDGFRB*** | chr5 | 149495321 | 149495514 | 194 | 194 | 0 |
| WG_IAD68410 | ***PDGFRB*** | chr5 | 149497176 | 149497418 | 243 | 243 | 0 |
| WG_IAD68410 | ***PDGFRB*** | chr5 | 149498305 | 149498420 | 116 | 116 | 0 |
| WG_IAD68410 | ***PDGFRB*** | chr5 | 149499025 | 149499134 | 110 | 110 | 0 |
| WG_IAD68410 | ***PDGFRB*** | chr5 | 149499570 | 149499691 | 122 | 122 | 0 |
| WG_IAD68410 | ***PDGFRB*** | chr5 | 149500446 | 149500578 | 133 | 133 | 0 |
| WG_IAD68410 | ***PDGFRB*** | chr5 | 149500762 | 149500890 | 129 | 129 | 0 |
| WG_IAD68410 | ***PDGFRB*** | chr5 | 149501438 | 149501608 | 171 | 171 | 0 |
| WG_IAD68410 | ***PDGFRB*** | chr5 | 149502600 | 149502769 | 170 | 170 | 0 |
| WG_IAD68410 | ***PDGFRB*** | chr5 | 149503808 | 149503928 | 121 | 121 | 0 |
| WG_IAD68410 | ***PDGFRB*** | chr5 | 149504285 | 149504399 | 115 | 115 | 0 |
| WG_IAD68410 | ***PDGFRB*** | chr5 | 149505003 | 149505145 | 143 | 143 | 0 |
| WG_IAD68410 | ***PDGFRB*** | chr5 | 149506078 | 149506182 | 105 | 105 | 0 |
| WG_IAD68410 | ***PDGFRB*** | chr5 | 149509315 | 149509536 | 222 | 222 | 0 |
| WG_IAD68410 | ***PDGFRB*** | chr5 | 149510097 | 149510230 | 134 | 134 | 0 |
| WG_IAD68410 | ***PDGFRB*** | chr5 | 149511537 | 149511662 | 126 | 126 | 0 |
| WG_IAD68410 | ***PDGFRB*** | chr5 | 149512308 | 149512510 | 203 | 203 | 0 |
| WG_IAD68410 | ***PDGFRB*** | chr5 | 149513144 | 149513328 | 185 | 185 | 0 |
| WG_IAD68410 | ***PDGFRB*** | chr5 | 149513439 | 149513576 | 138 | 138 | 0 |
| WG_IAD68410 | ***PDGFRB*** | chr5 | 149514308 | 149514584 | 277 | 277 | 0 |
| WG_IAD68410 | ***PDGFRB*** | chr5 | 149515113 | 149515446 | 334 | 334 | 0 |
| WG_IAD68410 | ***PDGFRB*** | chr5 | 149516566 | 149516615 | 50 | 50 | 0 |
| WG_IAD68410 | ***PIK3R1*** | chr5 | 67522499 | 67522842 | 344 | 344 | 0 |
| WG_IAD68410 | ***PIK3R1*** | chr5 | 67569213 | 67569315 | 103 | 103 | 0 |
| WG_IAD68410 | ***PIK3R1*** | chr5 | 67569762 | 67569846 | 85 | 85 | 0 |
| WG_IAD68410 | ***PIK3R1*** | chr5 | 67575425 | 67575566 | 142 | 142 | 0 |
| WG_IAD68410 | ***PIK3R1*** | chr5 | 67576351 | 67576562 | 212 | 212 | 0 |
| WG_IAD68410 | ***PIK3R1*** | chr5 | 67576750 | 67576839 | 90 | 90 | 0 |
| WG_IAD68410 | ***PIK3R1*** | chr5 | 67584559 | 67584584 | 26 | 26 | 0 |
| WG_IAD68410 | ***PIK3R1*** | chr5 | 67586552 | 67586667 | 116 | 116 | 0 |
| WG_IAD68410 | ***PIK3R1*** | chr5 | 67588082 | 67588194 | 113 | 113 | 0 |
| WG_IAD68410 | ***PIK3R1*** | chr5 | 67588924 | 67589032 | 109 | 109 | 0 |
| WG_IAD68410 | ***PIK3R1*** | chr5 | 67589126 | 67589316 | 191 | 191 | 0 |
| WG_IAD68410 | ***PIK3R1*** | chr5 | 67589532 | 67589667 | 133 | 136 | 3 |
| WG_IAD68410 | ***PIK3R1*** | chr5 | 67590359 | 67590511 | 153 | 153 | 0 |
| WG_IAD68410 | ***PIK3R1*** | chr5 | 67590971 | 67591157 | 187 | 187 | 0 |
| WG_IAD68410 | ***PIK3R1*** | chr5 | 67591243 | 67591321 | 79 | 79 | 0 |
| WG_IAD68410 | ***PIK3R1*** | chr5 | 67591994 | 67592174 | 181 | 181 | 0 |
| WG_IAD68410 | ***PIK3R1*** | chr5 | 67593235 | 67593434 | 200 | 200 | 0 |
| WG_IAD68410 | ***POLK*** | chr5 | 74842843 | 74842987 | 145 | 145 | 0 |
| WG_IAD68410 | ***POLK*** | chr5 | 74848292 | 74848421 | 130 | 130 | 0 |
| WG_IAD68410 | ***POLK*** | chr5 | 74865160 | 74865322 | 163 | 163 | 0 |
| WG_IAD68410 | ***POLK*** | chr5 | 74869558 | 74869699 | 142 | 142 | 0 |
| WG_IAD68410 | ***POLK*** | chr5 | 74872600 | 74872763 | 164 | 164 | 0 |
| WG_IAD68410 | ***POLK*** | chr5 | 74877029 | 74877278 | 250 | 250 | 0 |
| WG_IAD68410 | ***POLK*** | chr5 | 74879113 | 74879247 | 135 | 135 | 0 |
| WG_IAD68410 | ***POLK*** | chr5 | 74880580 | 74880756 | 177 | 177 | 0 |
| WG_IAD68410 | ***POLK*** | chr5 | 74882846 | 74882888 | 43 | 43 | 0 |
| WG_IAD68410 | ***POLK*** | chr5 | 74886164 | 74886270 | 107 | 107 | 0 |
| WG_IAD68410 | ***POLK*** | chr5 | 74889698 | 74889879 | 182 | 182 | 0 |
| WG_IAD68410 | ***POLK*** | chr5 | 74892042 | 74893008 | 967 | 967 | 0 |
| WG_IAD68410 | ***POLK*** | chr5 | 74893567 | 74893619 | 53 | 53 | 0 |
| WG_IAD68410 | ***POLK*** | chr5 | 74893754 | 74893848 | 95 | 95 | 0 |
| WG_IAD68410 | ***RAD50*** | chr5 | 131893012 | 131893150 | 139 | 139 | 0 |
| WG_IAD68410 | ***RAD50*** | chr5 | 131894971 | 131895064 | 94 | 94 | 0 |
| WG_IAD68410 | ***RAD50*** | chr5 | 131911464 | 131911625 | 162 | 162 | 0 |
| WG_IAD68410 | ***RAD50*** | chr5 | 131915004 | 131915199 | 196 | 196 | 0 |
| WG_IAD68410 | ***RAD50*** | chr5 | 131915549 | 131915763 | 215 | 215 | 0 |
| WG_IAD68410 | ***RAD50*** | chr5 | 131923249 | 131923387 | 139 | 139 | 0 |
| WG_IAD68410 | ***RAD50*** | chr5 | 131923611 | 131923786 | 176 | 176 | 0 |
| WG_IAD68410 | ***RAD50*** | chr5 | 131924374 | 131924577 | 204 | 204 | 0 |
| WG_IAD68410 | ***RAD50*** | chr5 | 131925318 | 131925534 | 217 | 217 | 0 |
| WG_IAD68410 | ***RAD50*** | chr5 | 131926911 | 131927103 | 193 | 193 | 0 |
| WG_IAD68410 | ***RAD50*** | chr5 | 131927564 | 131927731 | 168 | 168 | 0 |
| WG_IAD68410 | ***RAD50*** | chr5 | 131930556 | 131930741 | 186 | 186 | 0 |
| WG_IAD68410 | ***RAD50*** | chr5 | 131931260 | 131931507 | 248 | 248 | 0 |
| WG_IAD68410 | ***RAD50*** | chr5 | 131938987 | 131939186 | 200 | 200 | 0 |
| WG_IAD68410 | ***RAD50*** | chr5 | 131939607 | 131939743 | 137 | 137 | 0 |
| WG_IAD68410 | ***RAD50*** | chr5 | 131940493 | 131940696 | 188 | 204 | 16 |
| WG_IAD68410 | ***RAD50*** | chr5 | 131944302 | 131944422 | 121 | 121 | 0 |
| WG_IAD68410 | ***RAD50*** | chr5 | 131944804 | 131944906 | 103 | 103 | 0 |
| WG_IAD68410 | ***RAD50*** | chr5 | 131944970 | 131945093 | 81 | 124 | 43 |
| WG_IAD68410 | ***RAD50*** | chr5 | 131951690 | 131951827 | 138 | 138 | 0 |
| WG_IAD68410 | ***RAD50*** | chr5 | 131953757 | 131953991 | 235 | 235 | 0 |
| WG_IAD68410 | ***RAD50*** | chr5 | 131972802 | 131972897 | 96 | 96 | 0 |
| WG_IAD68410 | ***RAD50*** | chr5 | 131973768 | 131973920 | 153 | 153 | 0 |
| WG_IAD68410 | ***RAD50*** | chr5 | 131976359 | 131976502 | 144 | 144 | 0 |
| WG_IAD68410 | ***RAD50*** | chr5 | 131977865 | 131978061 | 197 | 197 | 0 |
| WG_IAD68410 | ***SDHA*** | chr5 | 218466 | 218538 | 73 | 73 | 0 |
| WG_IAD68410 | ***SDHA*** | chr5 | 223592 | 223688 | 97 | 97 | 0 |
| WG_IAD68410 | ***SDHA*** | chr5 | 224470 | 224641 | 172 | 172 | 0 |
| WG_IAD68410 | ***SDHA*** | chr5 | 225529 | 225682 | 154 | 154 | 0 |
| WG_IAD68410 | ***SDHA*** | chr5 | 225993 | 226167 | 175 | 175 | 0 |
| WG_IAD68410 | ***SDHA*** | chr5 | 228295 | 228453 | 159 | 159 | 0 |
| WG_IAD68410 | ***SDHA*** | chr5 | 230986 | 231120 | 135 | 135 | 0 |
| WG_IAD68410 | ***SDHA*** | chr5 | 233587 | 233765 | 179 | 179 | 0 |
| WG_IAD68410 | ***SDHA*** | chr5 | 235254 | 235459 | 206 | 206 | 0 |
| WG_IAD68410 | ***SDHA*** | chr5 | 236538 | 236719 | 182 | 182 | 0 |
| WG_IAD68410 | ***SDHA*** | chr5 | 240468 | 240596 | 129 | 129 | 0 |
| WG_IAD68410 | ***SDHA*** | chr5 | 251102 | 251223 | 122 | 122 | 0 |
| WG_IAD68410 | ***SDHA*** | chr5 | 251448 | 251588 | 141 | 141 | 0 |
| WG_IAD68410 | ***SDHA*** | chr5 | 254503 | 254626 | 42 | 124 | 82 |
| WG_IAD68410 | ***SDHA*** | chr5 | 256444 | 256540 | 97 | 97 | 0 |
| WG_IAD68410 | ***TERT*** | chr5 | 1253838 | 1253951 | 114 | 114 | 0 |
| WG_IAD68410 | ***TERT*** | chr5 | 1254478 | 1254625 | 148 | 148 | 0 |
| WG_IAD68410 | ***TERT*** | chr5 | 1255397 | 1255531 | 135 | 135 | 0 |
| WG_IAD68410 | ***TERT*** | chr5 | 1258708 | 1258779 | 72 | 72 | 0 |
| WG_IAD68410 | ***TERT*** | chr5 | 1260584 | 1260720 | 137 | 137 | 0 |
| WG_IAD68410 | ***TERT*** | chr5 | 1264514 | 1264712 | 199 | 199 | 0 |
| WG_IAD68410 | ***TERT*** | chr5 | 1266574 | 1266655 | 82 | 82 | 0 |
| WG_IAD68410 | ***TERT*** | chr5 | 1268630 | 1268753 | 124 | 124 | 0 |
| WG_IAD68410 | ***TERT*** | chr5 | 1271229 | 1271324 | 96 | 96 | 0 |
| WG_IAD68410 | ***TERT*** | chr5 | 1272295 | 1272400 | 106 | 106 | 0 |
| WG_IAD68410 | ***TERT*** | chr5 | 1278751 | 1278916 | 166 | 166 | 0 |
| WG_IAD68410 | ***TERT*** | chr5 | 1279401 | 1279590 | 190 | 190 | 0 |
| WG_IAD68410 | ***TERT*** | chr5 | 1280268 | 1280458 | 191 | 191 | 0 |
| WG_IAD68410 | ***TERT*** | chr5 | 1282539 | 1282744 | 206 | 206 | 0 |
| WG_IAD68410 | ***TERT*** | chr5 | 1293423 | 1294786 | 1364 | 1364 | 0 |
| WG_IAD68410 | ***TERT*** | chr5 | 1294881 | 1295109 | 229 | 229 | 0 |
| WG_IAD68410 | ***APC_15_HSPT*** | chr5 | 112173872 | 112176035 | 2164 | 2164 | 0 |
| WG_IAD68410 | ***CSF1R_22*** | chr5 | 149433608 | 149433787 | 180 | 180 | 0 |
| WG_IAD68410 | ***CSF1R_7*** | chr5 | 149452864 | 149453056 | 193 | 193 | 0 |
| WG_IAD68410 | ***NPM1_11*** | chr5 | 170837531 | 170837590 | 60 | 60 | 0 |
| WG_IAD68410 | ***DAXX*** | chr6 | 33286515 | 33286584 | 70 | 70 | 0 |
| WG_IAD68410 | ***DAXX*** | chr6 | 33286769 | 33287001 | 233 | 233 | 0 |
| WG_IAD68410 | ***DAXX*** | chr6 | 33287152 | 33287636 | 485 | 485 | 0 |
| WG_IAD68410 | ***DAXX*** | chr6 | 33287783 | 33288006 | 224 | 224 | 0 |
| WG_IAD68410 | ***DAXX*** | chr6 | 33288152 | 33288373 | 222 | 222 | 0 |
| WG_IAD68410 | ***DAXX*** | chr6 | 33288508 | 33289349 | 842 | 842 | 0 |
| WG_IAD68410 | ***DAXX*** | chr6 | 33289491 | 33289707 | 217 | 217 | 0 |
| WG_IAD68410 | ***DAXX*** | chr6 | 33290634 | 33290696 | 63 | 63 | 0 |
| WG_IAD68410 | ***IGF2R*** | chr6 | 160390274 | 160390432 | 83 | 159 | 76 |
| WG_IAD68410 | ***IGF2R*** | chr6 | 160412211 | 160412360 | 150 | 150 | 0 |
| WG_IAD68410 | ***IGF2R*** | chr6 | 160430037 | 160430171 | 135 | 135 | 0 |
| WG_IAD68410 | ***IGF2R*** | chr6 | 160431714 | 160431822 | 109 | 109 | 0 |
| WG_IAD68410 | ***IGF2R*** | chr6 | 160445599 | 160445741 | 143 | 143 | 0 |
| WG_IAD68410 | ***IGF2R*** | chr6 | 160448212 | 160448351 | 140 | 140 | 0 |
| WG_IAD68410 | ***IGF2R*** | chr6 | 160450577 | 160450692 | 116 | 116 | 0 |
| WG_IAD68410 | ***IGF2R*** | chr6 | 160453578 | 160453750 | 173 | 173 | 0 |
| WG_IAD68410 | ***IGF2R*** | chr6 | 160453969 | 160454144 | 176 | 176 | 0 |
| WG_IAD68410 | ***IGF2R*** | chr6 | 160455446 | 160455559 | 114 | 114 | 0 |
| WG_IAD68410 | ***IGF2R*** | chr6 | 160461587 | 160461761 | 175 | 175 | 0 |
| WG_IAD68410 | ***IGF2R*** | chr6 | 160464175 | 160464325 | 151 | 151 | 0 |
| WG_IAD68410 | ***IGF2R*** | chr6 | 160465541 | 160465694 | 154 | 154 | 0 |
| WG_IAD68410 | ***IGF2R*** | chr6 | 160466772 | 160466919 | 148 | 148 | 0 |
| WG_IAD68410 | ***IGF2R*** | chr6 | 160467525 | 160467682 | 158 | 158 | 0 |
| WG_IAD68410 | ***IGF2R*** | chr6 | 160468186 | 160468373 | 188 | 188 | 0 |
| WG_IAD68410 | ***IGF2R*** | chr6 | 160468819 | 160468944 | 126 | 126 | 0 |
| WG_IAD68410 | ***IGF2R*** | chr6 | 160469402 | 160469580 | 179 | 179 | 0 |
| WG_IAD68410 | ***IGF2R*** | chr6 | 160471500 | 160471689 | 190 | 190 | 0 |
| WG_IAD68410 | ***IGF2R*** | chr6 | 160477451 | 160477562 | 112 | 112 | 0 |
| WG_IAD68410 | ***IGF2R*** | chr6 | 160479050 | 160479161 | 112 | 112 | 0 |
| WG_IAD68410 | ***IGF2R*** | chr6 | 160479933 | 160480135 | 203 | 203 | 0 |
| WG_IAD68410 | ***IGF2R*** | chr6 | 160481574 | 160481754 | 181 | 181 | 0 |
| WG_IAD68410 | ***IGF2R*** | chr6 | 160482530 | 160482683 | 154 | 154 | 0 |
| WG_IAD68410 | ***IGF2R*** | chr6 | 160482780 | 160482965 | 186 | 186 | 0 |
| WG_IAD68410 | ***IGF2R*** | chr6 | 160483559 | 160483656 | 98 | 98 | 0 |
| WG_IAD68410 | ***IGF2R*** | chr6 | 160484442 | 160484667 | 226 | 226 | 0 |
| WG_IAD68410 | ***IGF2R*** | chr6 | 160485428 | 160485568 | 141 | 141 | 0 |
| WG_IAD68410 | ***IGF2R*** | chr6 | 160485831 | 160485938 | 108 | 108 | 0 |
| WG_IAD68410 | ***IGF2R*** | chr6 | 160489276 | 160489422 | 147 | 147 | 0 |
| WG_IAD68410 | ***IGF2R*** | chr6 | 160490895 | 160491095 | 201 | 201 | 0 |
| WG_IAD68410 | ***IGF2R*** | chr6 | 160492937 | 160493073 | 137 | 137 | 0 |
| WG_IAD68410 | ***IGF2R*** | chr6 | 160493792 | 160493921 | 130 | 130 | 0 |
| WG_IAD68410 | ***IGF2R*** | chr6 | 160494240 | 160494506 | 267 | 267 | 0 |
| WG_IAD68410 | ***IGF2R*** | chr6 | 160494784 | 160495012 | 229 | 229 | 0 |
| WG_IAD68410 | ***IGF2R*** | chr6 | 160496874 | 160497033 | 160 | 160 | 0 |
| WG_IAD68410 | ***IGF2R*** | chr6 | 160499228 | 160499399 | 172 | 172 | 0 |
| WG_IAD68410 | ***IGF2R*** | chr6 | 160500607 | 160500824 | 218 | 218 | 0 |
| WG_IAD68410 | ***IGF2R*** | chr6 | 160501156 | 160501312 | 157 | 157 | 0 |
| WG_IAD68410 | ***IGF2R*** | chr6 | 160504977 | 160505221 | 245 | 245 | 0 |
| WG_IAD68410 | ***IGF2R*** | chr6 | 160506022 | 160506168 | 147 | 147 | 0 |
| WG_IAD68410 | ***IGF2R*** | chr6 | 160509060 | 160509184 | 125 | 125 | 0 |
| WG_IAD68410 | ***IGF2R*** | chr6 | 160510134 | 160510290 | 157 | 157 | 0 |
| WG_IAD68410 | ***IGF2R*** | chr6 | 160510943 | 160511140 | 198 | 198 | 0 |
| WG_IAD68410 | ***IGF2R*** | chr6 | 160517466 | 160517662 | 197 | 197 | 0 |
| WG_IAD68410 | ***IGF2R*** | chr6 | 160523546 | 160523708 | 163 | 163 | 0 |
| WG_IAD68410 | ***IGF2R*** | chr6 | 160524773 | 160524852 | 80 | 80 | 0 |
| WG_IAD68410 | ***IGF2R*** | chr6 | 160525701 | 160526121 | 421 | 421 | 0 |
| WG_IAD68410 | ***T*** | chr6 | 166571798 | 166572081 | 284 | 284 | 0 |
| WG_IAD68410 | ***T*** | chr6 | 166574320 | 166574459 | 140 | 140 | 0 |
| WG_IAD68410 | ***T*** | chr6 | 166575930 | 166576113 | 184 | 184 | 0 |
| WG_IAD68410 | ***T*** | chr6 | 166578088 | 166578159 | 72 | 72 | 0 |
| WG_IAD68410 | ***T*** | chr6 | 166578283 | 166578354 | 72 | 72 | 0 |
| WG_IAD68410 | ***T*** | chr6 | 166579189 | 166579333 | 145 | 145 | 0 |
| WG_IAD68410 | ***T*** | chr6 | 166580075 | 166580349 | 275 | 275 | 0 |
| WG_IAD68410 | ***T*** | chr6 | 166580869 | 166581084 | 216 | 216 | 0 |
| WG_IAD68410 | ***TNF*** | chr6 | 31543514 | 31543709 | 196 | 196 | 0 |
| WG_IAD68410 | ***TNF*** | chr6 | 31544306 | 31544361 | 56 | 56 | 0 |
| WG_IAD68410 | ***TNF*** | chr6 | 31544539 | 31544596 | 58 | 58 | 0 |
| WG_IAD68410 | ***TNF*** | chr6 | 31544888 | 31545319 | 432 | 432 | 0 |
| WG_IAD68410 | ***GRM3*** | chr7 | 86394457 | 86394934 | 478 | 478 | 0 |
| WG_IAD68410 | ***GRM3*** | chr7 | 86415572 | 86416437 | 866 | 866 | 0 |
| WG_IAD68410 | ***GRM3*** | chr7 | 86468150 | 86469226 | 1077 | 1077 | 0 |
| WG_IAD68410 | ***GRM3*** | chr7 | 86479681 | 86479865 | 185 | 185 | 0 |
| WG_IAD68410 | ***GRM3*** | chr7 | 86493593 | 86493676 | 84 | 84 | 0 |
| WG_IAD68410 | ***KMT2C*** | chr7 | 151833912 | 151834014 | 103 | 103 | 0 |
| WG_IAD68410 | ***KMT2C*** | chr7 | 151835876 | 151835994 | 119 | 119 | 0 |
| WG_IAD68410 | ***KMT2C*** | chr7 | 151836266 | 151836349 | 84 | 84 | 0 |
| WG_IAD68410 | ***KMT2C*** | chr7 | 151836755 | 151836881 | 127 | 127 | 0 |
| WG_IAD68410 | ***KMT2C*** | chr7 | 151841793 | 151841971 | 179 | 179 | 0 |
| WG_IAD68410 | ***KMT2C*** | chr7 | 151842233 | 151842385 | 153 | 153 | 0 |
| WG_IAD68410 | ***KMT2C*** | chr7 | 151843679 | 151843825 | 147 | 147 | 0 |
| WG_IAD68410 | ***KMT2C*** | chr7 | 151845113 | 151846242 | 1130 | 1130 | 0 |
| WG_IAD68410 | ***KMT2C*** | chr7 | 151847980 | 151848097 | 118 | 118 | 0 |
| WG_IAD68410 | ***KMT2C*** | chr7 | 151848522 | 151848671 | 150 | 150 | 0 |
| WG_IAD68410 | ***KMT2C*** | chr7 | 151849785 | 151850044 | 260 | 260 | 0 |
| WG_IAD68410 | ***KMT2C*** | chr7 | 151851090 | 151851236 | 147 | 147 | 0 |
| WG_IAD68410 | ***KMT2C*** | chr7 | 151851347 | 151851535 | 189 | 189 | 0 |
| WG_IAD68410 | ***KMT2C*** | chr7 | 151852990 | 151853147 | 158 | 158 | 0 |
| WG_IAD68410 | ***KMT2C*** | chr7 | 151853285 | 151853436 | 152 | 152 | 0 |
| WG_IAD68410 | ***KMT2C*** | chr7 | 151855943 | 151856162 | 220 | 220 | 0 |
| WG_IAD68410 | ***KMT2C*** | chr7 | 151859197 | 151860916 | 1720 | 1720 | 0 |
| WG_IAD68410 | ***KMT2C*** | chr7 | 151864226 | 151864468 | 243 | 243 | 0 |
| WG_IAD68410 | ***KMT2C*** | chr7 | 151866266 | 151866339 | 74 | 74 | 0 |
| WG_IAD68410 | ***KMT2C*** | chr7 | 151868344 | 151868432 | 89 | 89 | 0 |
| WG_IAD68410 | ***KMT2C*** | chr7 | 151871211 | 151871332 | 122 | 122 | 0 |
| WG_IAD68410 | ***KMT2C*** | chr7 | 151873271 | 151875100 | 1806 | 1830 | 24 |
| WG_IAD68410 | ***KMT2C*** | chr7 | 151876914 | 151877216 | 303 | 303 | 0 |
| WG_IAD68410 | ***KMT2C*** | chr7 | 151877791 | 151879684 | 1894 | 1894 | 0 |
| WG_IAD68410 | ***KMT2C*** | chr7 | 151880054 | 151880246 | 193 | 193 | 0 |
| WG_IAD68410 | ***KMT2C*** | chr7 | 151882638 | 151882721 | 84 | 84 | 0 |
| WG_IAD68410 | ***KMT2C*** | chr7 | 151884342 | 151884566 | 225 | 225 | 0 |
| WG_IAD68410 | ***KMT2C*** | chr7 | 151884795 | 151884937 | 143 | 143 | 0 |
| WG_IAD68410 | ***KMT2C*** | chr7 | 151891089 | 151891218 | 130 | 130 | 0 |
| WG_IAD68410 | ***KMT2C*** | chr7 | 151891309 | 151891351 | 3 | 43 | 40 |
| WG_IAD68410 | ***KMT2C*** | chr7 | 151891520 | 151891658 | 139 | 139 | 0 |
| WG_IAD68410 | ***KMT2C*** | chr7 | 151892987 | 151893101 | 115 | 115 | 0 |
| WG_IAD68410 | ***KMT2C*** | chr7 | 151896359 | 151896549 | 191 | 191 | 0 |
| WG_IAD68410 | ***KMT2C*** | chr7 | 151900014 | 151900154 | 141 | 141 | 0 |
| WG_IAD68410 | ***KMT2C*** | chr7 | 151902186 | 151902315 | 130 | 130 | 0 |
| WG_IAD68410 | ***KMT2C*** | chr7 | 151904380 | 151904518 | 0 | 139 | 139 |
| WG_IAD68410 | ***KMT2C*** | chr7 | 151917603 | 151917825 | 223 | 223 | 0 |
| WG_IAD68410 | ***KMT2C*** | chr7 | 151919081 | 151919156 | 76 | 76 | 0 |
| WG_IAD68410 | ***KMT2C*** | chr7 | 151919653 | 151919772 | 120 | 120 | 0 |
| WG_IAD68410 | ***KMT2C*** | chr7 | 151921095 | 151921269 | 129 | 175 | 46 |
| WG_IAD68410 | ***KMT2C*** | chr7 | 151921515 | 151921706 | 142 | 192 | 50 |
| WG_IAD68410 | ***KMT2C*** | chr7 | 151927003 | 151927117 | 10 | 115 | 105 |
| WG_IAD68410 | ***KMT2C*** | chr7 | 151927300 | 151927411 | 112 | 112 | 0 |
| WG_IAD68410 | ***KMT2C*** | chr7 | 151932897 | 151933023 | 127 | 127 | 0 |
| WG_IAD68410 | ***KMT2C*** | chr7 | 151935787 | 151935916 | 130 | 130 | 0 |
| WG_IAD68410 | ***KMT2C*** | chr7 | 151944982 | 151945710 | 700 | 729 | 29 |
| WG_IAD68410 | ***KMT2C*** | chr7 | 151946956 | 151947043 | 88 | 88 | 0 |
| WG_IAD68410 | ***KMT2C*** | chr7 | 151947933 | 151948056 | 124 | 124 | 0 |
| WG_IAD68410 | ***KMT2C*** | chr7 | 151949019 | 151949180 | 162 | 162 | 0 |
| WG_IAD68410 | ***KMT2C*** | chr7 | 151949626 | 151949805 | 180 | 180 | 0 |
| WG_IAD68410 | ***KMT2C*** | chr7 | 151960096 | 151960220 | 125 | 125 | 0 |
| WG_IAD68410 | ***KMT2C*** | chr7 | 151962118 | 151962299 | 99 | 182 | 83 |
| WG_IAD68410 | ***KMT2C*** | chr7 | 151970785 | 151970957 | 173 | 173 | 0 |
| WG_IAD68410 | ***KMT2C*** | chr7 | 152007046 | 152007165 | 120 | 120 | 0 |
| WG_IAD68410 | ***KMT2C*** | chr7 | 152008878 | 152009036 | 159 | 159 | 0 |
| WG_IAD68410 | ***KMT2C*** | chr7 | 152012218 | 152012428 | 211 | 211 | 0 |
| WG_IAD68410 | ***KMT2C*** | chr7 | 152027681 | 152027829 | 149 | 149 | 0 |
| WG_IAD68410 | ***KMT2C*** | chr7 | 152055667 | 152055765 | 99 | 99 | 0 |
| WG_IAD68410 | ***KMT2C*** | chr7 | 152132706 | 152132876 | 158 | 171 | 13 |
| WG_IAD68410 | ***PMS2*** | chr7 | 6013025 | 6013178 | 26 | 154 | 128 |
| WG_IAD68410 | ***PMS2*** | chr7 | 6017214 | 6017393 | 0 | 180 | 180 |
| WG_IAD68410 | ***PMS2*** | chr7 | 6018222 | 6018332 | 0 | 111 | 111 |
| WG_IAD68410 | ***PMS2*** | chr7 | 6022450 | 6022627 | 57 | 178 | 121 |
| WG_IAD68410 | ***PMS2*** | chr7 | 6026385 | 6027256 | 843 | 872 | 29 |
| WG_IAD68410 | ***PMS2*** | chr7 | 6029426 | 6029591 | 166 | 166 | 0 |
| WG_IAD68410 | ***PMS2*** | chr7 | 6031599 | 6031693 | 95 | 95 | 0 |
| WG_IAD68410 | ***PMS2*** | chr7 | 6035160 | 6035269 | 110 | 110 | 0 |
| WG_IAD68410 | ***PMS2*** | chr7 | 6036952 | 6037059 | 108 | 108 | 0 |
| WG_IAD68410 | ***PMS2*** | chr7 | 6038734 | 6038911 | 178 | 178 | 0 |
| WG_IAD68410 | ***PMS2*** | chr7 | 6042079 | 6042272 | 146 | 194 | 48 |
| WG_IAD68410 | ***PMS2*** | chr7 | 6043316 | 6043428 | 113 | 113 | 0 |
| WG_IAD68410 | ***PMS2*** | chr7 | 6043598 | 6043694 | 97 | 97 | 0 |
| WG_IAD68410 | ***PMS2*** | chr7 | 6045518 | 6045667 | 147 | 150 | 3 |
| WG_IAD68410 | ***PMS2*** | chr7 | 6048623 | 6048655 | 33 | 33 | 0 |
| WG_IAD68410 | ***RAC1*** | chr7 | 6414362 | 6414406 | 45 | 45 | 0 |
| WG_IAD68410 | ***RAC1*** | chr7 | 6426838 | 6426919 | 82 | 82 | 0 |
| WG_IAD68410 | ***RAC1*** | chr7 | 6431550 | 6431677 | 128 | 128 | 0 |
| WG_IAD68410 | ***RAC1*** | chr7 | 6438288 | 6438354 | 67 | 67 | 0 |
| WG_IAD68410 | ***RAC1*** | chr7 | 6439752 | 6439824 | 73 | 73 | 0 |
| WG_IAD68410 | ***RAC1*** | chr7 | 6441494 | 6441663 | 170 | 170 | 0 |
| WG_IAD68410 | ***RAC1*** | chr7 | 6441942 | 6442082 | 141 | 141 | 0 |
| WG_IAD68410 | ***EGFR_3*** | chr7 | 55210998 | 55211181 | 184 | 184 | 0 |
| WG_IAD68410 | ***EGFR_7*** | chr7 | 55221704 | 55221845 | 142 | 142 | 0 |
| WG_IAD68410 | ***EGFR_15*** | chr7 | 55232973 | 55233130 | 158 | 158 | 0 |
| WG_IAD68410 | ***EGFR_17*** | chr7 | 55240676 | 55240817 | 142 | 142 | 0 |
| WG_IAD68410 | ***EGFR_18*** | chr7 | 55241614 | 55241736 | 123 | 123 | 0 |
| WG_IAD68410 | ***EGFR_19*** | chr7 | 55242436 | 55242485 | 50 | 50 | 0 |
| WG_IAD68410 | ***EGFR_20*** | chr7 | 55248986 | 55249171 | 186 | 186 | 0 |
| WG_IAD68410 | ***EGFR_21*** | chr7 | 55259412 | 55259567 | 156 | 156 | 0 |
| WG_IAD68410 | ***EGFR_22*** | chr7 | 55260459 | 55260534 | 76 | 76 | 0 |
| WG_IAD68410 | ***MET_2*** | chr7 | 116339125 | 116340338 | 1214 | 1214 | 0 |
| WG_IAD68410 | ***MET_11*** | chr7 | 116403104 | 116403322 | 219 | 219 | 0 |
| WG_IAD68410 | ***MET_14*** | chr7 | 116411903 | 116412043 | 141 | 141 | 0 |
| WG_IAD68410 | ***MET_15*** | chr7 | 116414935 | 116415165 | 231 | 231 | 0 |
| WG_IAD68410 | ***MET_16*** | chr7 | 116417443 | 116417523 | 81 | 81 | 0 |
| WG_IAD68410 | ***MET_17*** | chr7 | 116418830 | 116419011 | 182 | 182 | 0 |
| WG_IAD68410 | ***MET_18*** | chr7 | 116422042 | 116422151 | 110 | 110 | 0 |
| WG_IAD68410 | ***MET_19*** | chr7 | 116423358 | 116423523 | 166 | 166 | 0 |
| WG_IAD68410 | ***SMO_3*** | chr7 | 128845044 | 128845253 | 210 | 210 | 0 |
| WG_IAD68410 | ***SMO_5*** | chr7 | 128845991 | 128846210 | 220 | 220 | 0 |
| WG_IAD68410 | ***SMO_6*** | chr7 | 128846305 | 128846428 | 124 | 124 | 0 |
| WG_IAD68410 | ***SMO_9*** | chr7 | 128850204 | 128850389 | 186 | 186 | 0 |
| WG_IAD68410 | ***SMO_11*** | chr7 | 128851477 | 128851611 | 135 | 135 | 0 |
| WG_IAD68410 | ***BRAF_15*** | chr7 | 140453075 | 140453193 | 119 | 119 | 0 |
| WG_IAD68410 | ***BRAF_11*** | chr7 | 140481376 | 140481493 | 118 | 118 | 0 |
| WG_IAD68410 | ***EZH2_15*** | chr7 | 148508717 | 148508812 | 96 | 96 | 0 |
| WG_IAD68410 | ***PRKDC*** | chr8 | 48686729 | 48686943 | 215 | 215 | 0 |
| WG_IAD68410 | ***PRKDC*** | chr8 | 48689400 | 48689549 | 150 | 150 | 0 |
| WG_IAD68410 | ***PRKDC*** | chr8 | 48690242 | 48690440 | 199 | 199 | 0 |
| WG_IAD68410 | ***PRKDC*** | chr8 | 48691015 | 48691226 | 212 | 212 | 0 |
| WG_IAD68410 | ***PRKDC*** | chr8 | 48691284 | 48691365 | 82 | 82 | 0 |
| WG_IAD68410 | ***PRKDC*** | chr8 | 48691560 | 48691659 | 100 | 100 | 0 |
| WG_IAD68410 | ***PRKDC*** | chr8 | 48694718 | 48694820 | 103 | 103 | 0 |
| WG_IAD68410 | ***PRKDC*** | chr8 | 48694934 | 48695164 | 231 | 231 | 0 |
| WG_IAD68410 | ***PRKDC*** | chr8 | 48696298 | 48696375 | 78 | 78 | 0 |
| WG_IAD68410 | ***PRKDC*** | chr8 | 48697669 | 48697883 | 215 | 215 | 0 |
| WG_IAD68410 | ***PRKDC*** | chr8 | 48701462 | 48701615 | 154 | 154 | 0 |
| WG_IAD68410 | ***PRKDC*** | chr8 | 48701707 | 48701804 | 98 | 98 | 0 |
| WG_IAD68410 | ***PRKDC*** | chr8 | 48706846 | 48707067 | 222 | 222 | 0 |
| WG_IAD68410 | ***PRKDC*** | chr8 | 48710793 | 48710963 | 171 | 171 | 0 |
| WG_IAD68410 | ***PRKDC*** | chr8 | 48711766 | 48711956 | 191 | 191 | 0 |
| WG_IAD68410 | ***PRKDC*** | chr8 | 48713349 | 48713552 | 204 | 204 | 0 |
| WG_IAD68410 | ***PRKDC*** | chr8 | 48715862 | 48716046 | 185 | 185 | 0 |
| WG_IAD68410 | ***PRKDC*** | chr8 | 48719693 | 48719892 | 200 | 200 | 0 |
| WG_IAD68410 | ***PRKDC*** | chr8 | 48730006 | 48730127 | 122 | 122 | 0 |
| WG_IAD68410 | ***PRKDC*** | chr8 | 48731958 | 48732076 | 84 | 119 | 35 |
| WG_IAD68410 | ***PRKDC*** | chr8 | 48733275 | 48733509 | 210 | 235 | 25 |
| WG_IAD68410 | ***PRKDC*** | chr8 | 48734160 | 48734358 | 199 | 199 | 0 |
| WG_IAD68410 | ***PRKDC*** | chr8 | 48736414 | 48736562 | 149 | 149 | 0 |
| WG_IAD68410 | ***PRKDC*** | chr8 | 48739212 | 48739427 | 216 | 216 | 0 |
| WG_IAD68410 | ***PRKDC*** | chr8 | 48740724 | 48740913 | 190 | 190 | 0 |
| WG_IAD68410 | ***PRKDC*** | chr8 | 48743161 | 48743302 | 142 | 142 | 0 |
| WG_IAD68410 | ***PRKDC*** | chr8 | 48744370 | 48744492 | 123 | 123 | 0 |
| WG_IAD68410 | ***PRKDC*** | chr8 | 48746752 | 48746962 | 211 | 211 | 0 |
| WG_IAD68410 | ***PRKDC*** | chr8 | 48748894 | 48749093 | 200 | 200 | 0 |
| WG_IAD68410 | ***PRKDC*** | chr8 | 48749768 | 48749985 | 218 | 218 | 0 |
| WG_IAD68410 | ***PRKDC*** | chr8 | 48751704 | 48751812 | 109 | 109 | 0 |
| WG_IAD68410 | ***PRKDC*** | chr8 | 48752572 | 48752755 | 184 | 184 | 0 |
| WG_IAD68410 | ***PRKDC*** | chr8 | 48761710 | 48761869 | 160 | 160 | 0 |
| WG_IAD68410 | ***PRKDC*** | chr8 | 48761935 | 48762069 | 135 | 135 | 0 |
| WG_IAD68410 | ***PRKDC*** | chr8 | 48765229 | 48765350 | 122 | 122 | 0 |
| WG_IAD68410 | ***PRKDC*** | chr8 | 48766639 | 48766780 | 142 | 142 | 0 |
| WG_IAD68410 | ***PRKDC*** | chr8 | 48767778 | 48767939 | 162 | 162 | 0 |
| WG_IAD68410 | ***PRKDC*** | chr8 | 48769712 | 48769865 | 154 | 154 | 0 |
| WG_IAD68410 | ***PRKDC*** | chr8 | 48771072 | 48771201 | 130 | 130 | 0 |
| WG_IAD68410 | ***PRKDC*** | chr8 | 48771405 | 48771552 | 148 | 148 | 0 |
| WG_IAD68410 | ***PRKDC*** | chr8 | 48772167 | 48772325 | 159 | 159 | 0 |
| WG_IAD68410 | ***PRKDC*** | chr8 | 48773455 | 48773537 | 83 | 83 | 0 |
| WG_IAD68410 | ***PRKDC*** | chr8 | 48774618 | 48774693 | 76 | 76 | 0 |
| WG_IAD68410 | ***PRKDC*** | chr8 | 48774929 | 48775107 | 179 | 179 | 0 |
| WG_IAD68410 | ***PRKDC*** | chr8 | 48775955 | 48776143 | 189 | 189 | 0 |
| WG_IAD68410 | ***PRKDC*** | chr8 | 48777112 | 48777329 | 218 | 218 | 0 |
| WG_IAD68410 | ***PRKDC*** | chr8 | 48790280 | 48790417 | 138 | 138 | 0 |
| WG_IAD68410 | ***PRKDC*** | chr8 | 48792047 | 48792224 | 178 | 178 | 0 |
| WG_IAD68410 | ***PRKDC*** | chr8 | 48793972 | 48794086 | 115 | 115 | 0 |
| WG_IAD68410 | ***PRKDC*** | chr8 | 48794468 | 48794663 | 196 | 196 | 0 |
| WG_IAD68410 | ***PRKDC*** | chr8 | 48798500 | 48798713 | 214 | 214 | 0 |
| WG_IAD68410 | ***PRKDC*** | chr8 | 48800103 | 48800271 | 169 | 169 | 0 |
| WG_IAD68410 | ***PRKDC*** | chr8 | 48801074 | 48801216 | 143 | 143 | 0 |
| WG_IAD68410 | ***PRKDC*** | chr8 | 48801570 | 48801788 | 219 | 219 | 0 |
| WG_IAD68410 | ***PRKDC*** | chr8 | 48802813 | 48803046 | 234 | 234 | 0 |
| WG_IAD68410 | ***PRKDC*** | chr8 | 48805695 | 48805952 | 258 | 258 | 0 |
| WG_IAD68410 | ***PRKDC*** | chr8 | 48809716 | 48809859 | 144 | 144 | 0 |
| WG_IAD68410 | ***PRKDC*** | chr8 | 48811025 | 48811134 | 110 | 110 | 0 |
| WG_IAD68410 | ***PRKDC*** | chr8 | 48812928 | 48813032 | 105 | 105 | 0 |
| WG_IAD68410 | ***PRKDC*** | chr8 | 48815124 | 48815360 | 237 | 237 | 0 |
| WG_IAD68410 | ***PRKDC*** | chr8 | 48817424 | 48817541 | 118 | 118 | 0 |
| WG_IAD68410 | ***PRKDC*** | chr8 | 48824965 | 48825127 | 163 | 163 | 0 |
| WG_IAD68410 | ***PRKDC*** | chr8 | 48826456 | 48826629 | 174 | 174 | 0 |
| WG_IAD68410 | ***PRKDC*** | chr8 | 48827883 | 48827983 | 101 | 101 | 0 |
| WG_IAD68410 | ***PRKDC*** | chr8 | 48830832 | 48830948 | 117 | 117 | 0 |
| WG_IAD68410 | ***PRKDC*** | chr8 | 48839749 | 48839918 | 91 | 170 | 79 |
| WG_IAD68410 | ***PRKDC*** | chr8 | 48840326 | 48840455 | 130 | 130 | 0 |
| WG_IAD68410 | ***PRKDC*** | chr8 | 48841647 | 48841743 | 97 | 97 | 0 |
| WG_IAD68410 | ***PRKDC*** | chr8 | 48842408 | 48842577 | 170 | 170 | 0 |
| WG_IAD68410 | ***PRKDC*** | chr8 | 48843227 | 48843352 | 126 | 126 | 0 |
| WG_IAD68410 | ***PRKDC*** | chr8 | 48845575 | 48845737 | 163 | 163 | 0 |
| WG_IAD68410 | ***PRKDC*** | chr8 | 48846520 | 48846655 | 136 | 136 | 0 |
| WG_IAD68410 | ***PRKDC*** | chr8 | 48847564 | 48847623 | 60 | 60 | 0 |
| WG_IAD68410 | ***PRKDC*** | chr8 | 48848287 | 48848465 | 179 | 179 | 0 |
| WG_IAD68410 | ***PRKDC*** | chr8 | 48848908 | 48849082 | 175 | 175 | 0 |
| WG_IAD68410 | ***PRKDC*** | chr8 | 48852106 | 48852262 | 157 | 157 | 0 |
| WG_IAD68410 | ***PRKDC*** | chr8 | 48855764 | 48855931 | 168 | 168 | 0 |
| WG_IAD68410 | ***PRKDC*** | chr8 | 48856408 | 48856448 | 41 | 41 | 0 |
| WG_IAD68410 | ***PRKDC*** | chr8 | 48856529 | 48856594 | 66 | 66 | 0 |
| WG_IAD68410 | ***PRKDC*** | chr8 | 48866175 | 48866284 | 110 | 110 | 0 |
| WG_IAD68410 | ***PRKDC*** | chr8 | 48866362 | 48866484 | 123 | 123 | 0 |
| WG_IAD68410 | ***PRKDC*** | chr8 | 48866893 | 48867011 | 119 | 119 | 0 |
| WG_IAD68410 | ***PRKDC*** | chr8 | 48868429 | 48868513 | 85 | 85 | 0 |
| WG_IAD68410 | ***PRKDC*** | chr8 | 48869726 | 48869828 | 103 | 103 | 0 |
| WG_IAD68410 | ***PRKDC*** | chr8 | 48869910 | 48869996 | 87 | 87 | 0 |
| WG_IAD68410 | ***PRKDC*** | chr8 | 48872528 | 48872691 | 164 | 164 | 0 |
| WG_IAD68410 | ***SNX31*** | chr8 | 101586088 | 101586193 | 106 | 106 | 0 |
| WG_IAD68410 | ***SNX31*** | chr8 | 101589242 | 101589308 | 67 | 67 | 0 |
| WG_IAD68410 | ***SNX31*** | chr8 | 101596334 | 101596421 | 88 | 88 | 0 |
| WG_IAD68410 | ***SNX31*** | chr8 | 101601089 | 101601212 | 124 | 124 | 0 |
| WG_IAD68410 | ***SNX31*** | chr8 | 101608862 | 101609075 | 214 | 214 | 0 |
| WG_IAD68410 | ***SNX31*** | chr8 | 101612572 | 101612674 | 103 | 103 | 0 |
| WG_IAD68410 | ***SNX31*** | chr8 | 101620717 | 101620796 | 80 | 80 | 0 |
| WG_IAD68410 | ***SNX31*** | chr8 | 101624223 | 101624320 | 98 | 98 | 0 |
| WG_IAD68410 | ***SNX31*** | chr8 | 101625218 | 101625318 | 101 | 101 | 0 |
| WG_IAD68410 | ***SNX31*** | chr8 | 101629843 | 101629963 | 121 | 121 | 0 |
| WG_IAD68410 | ***SNX31*** | chr8 | 101642550 | 101642624 | 75 | 75 | 0 |
| WG_IAD68410 | ***SNX31*** | chr8 | 101648120 | 101648244 | 125 | 125 | 0 |
| WG_IAD68410 | ***SNX31*** | chr8 | 101661497 | 101661581 | 85 | 85 | 0 |
| WG_IAD68410 | ***SNX31*** | chr8 | 101661672 | 101661747 | 76 | 76 | 0 |
| WG_IAD68410 | ***FGFR1_16*** | chr8 | 38271670 | 38271807 | 138 | 138 | 0 |
| WG_IAD68410 | ***FGFR1_15*** | chr8 | 38272077 | 38272147 | 71 | 71 | 0 |
| WG_IAD68410 | ***FGFR1_14*** | chr8 | 38272297 | 38272419 | 123 | 123 | 0 |
| WG_IAD68410 | ***FGFR1_13*** | chr8 | 38273388 | 38273578 | 191 | 191 | 0 |
| WG_IAD68410 | ***FGFR1_12*** | chr8 | 38274824 | 38274934 | 111 | 111 | 0 |
| WG_IAD68410 | ***FGFR1_11*** | chr8 | 38275388 | 38275509 | 122 | 122 | 0 |
| WG_IAD68410 | ***FGFR1_10*** | chr8 | 38275746 | 38275891 | 146 | 146 | 0 |
| WG_IAD68410 | ***FGFR1_9*** | chr8 | 38277051 | 38277253 | 203 | 203 | 0 |
| WG_IAD68410 | ***FGFR1_7*** | chr8 | 38282027 | 38282217 | 191 | 191 | 0 |
| WG_IAD68410 | ***FGFR1_4*** | chr8 | 38285864 | 38285953 | 90 | 90 | 0 |
| WG_IAD68410 | ***TACC1_5*** | chr8 | 38684686 | 38684893 | 208 | 208 | 0 |
| WG_IAD68410 | ***TACC1_10*** | chr8 | 38699805 | 38699965 | 161 | 161 | 0 |
| WG_IAD68410 | ***TACC1_11*** | chr8 | 38700807 | 38700913 | 107 | 107 | 0 |
| WG_IAD68410 | ***PTCH1*** | chr9 | 98209189 | 98209738 | 550 | 550 | 0 |
| WG_IAD68410 | ***PTCH1*** | chr9 | 98211346 | 98211610 | 265 | 265 | 0 |
| WG_IAD68410 | ***PTCH1*** | chr9 | 98212118 | 98212227 | 110 | 110 | 0 |
| WG_IAD68410 | ***PTCH1*** | chr9 | 98215755 | 98215907 | 153 | 153 | 0 |
| WG_IAD68410 | ***PTCH1*** | chr9 | 98218553 | 98218700 | 148 | 148 | 0 |
| WG_IAD68410 | ***PTCH1*** | chr9 | 98220290 | 98220580 | 291 | 291 | 0 |
| WG_IAD68410 | ***PTCH1*** | chr9 | 98221877 | 98222070 | 194 | 194 | 0 |
| WG_IAD68410 | ***PTCH1*** | chr9 | 98224133 | 98224285 | 153 | 153 | 0 |
| WG_IAD68410 | ***PTCH1*** | chr9 | 98229393 | 98229712 | 320 | 320 | 0 |
| WG_IAD68410 | ***PTCH1*** | chr9 | 98231028 | 98231440 | 413 | 413 | 0 |
| WG_IAD68410 | ***PTCH1*** | chr9 | 98232090 | 98232218 | 129 | 129 | 0 |
| WG_IAD68410 | ***PTCH1*** | chr9 | 98238311 | 98238446 | 136 | 136 | 0 |
| WG_IAD68410 | ***PTCH1*** | chr9 | 98239036 | 98239144 | 109 | 109 | 0 |
| WG_IAD68410 | ***PTCH1*** | chr9 | 98239824 | 98239989 | 166 | 166 | 0 |
| WG_IAD68410 | ***PTCH1*** | chr9 | 98240332 | 98240473 | 142 | 142 | 0 |
| WG_IAD68410 | ***PTCH1*** | chr9 | 98241277 | 98241434 | 158 | 158 | 0 |
| WG_IAD68410 | ***PTCH1*** | chr9 | 98242246 | 98242377 | 132 | 132 | 0 |
| WG_IAD68410 | ***PTCH1*** | chr9 | 98242667 | 98242875 | 209 | 209 | 0 |
| WG_IAD68410 | ***PTCH1*** | chr9 | 98244226 | 98244327 | 102 | 102 | 0 |
| WG_IAD68410 | ***PTCH1*** | chr9 | 98244411 | 98244490 | 80 | 80 | 0 |
| WG_IAD68410 | ***PTCH1*** | chr9 | 98247962 | 98248161 | 200 | 200 | 0 |
| WG_IAD68410 | ***PTCH1*** | chr9 | 98268684 | 98268886 | 203 | 203 | 0 |
| WG_IAD68410 | ***PTCH1*** | chr9 | 98270438 | 98270648 | 202 | 211 | 9 |
| WG_IAD68410 | ***PTCH1*** | chr9 | 98278746 | 98278758 | 13 | 13 | 0 |
| WG_IAD68410 | ***PTCH1*** | chr9 | 98278900 | 98279107 | 208 | 208 | 0 |
| WG_IAD68410 | ***TSC1*** | chr9 | 135771617 | 135772146 | 530 | 530 | 0 |
| WG_IAD68410 | ***TSC1*** | chr9 | 135772566 | 135772737 | 172 | 172 | 0 |
| WG_IAD68410 | ***TSC1*** | chr9 | 135772805 | 135773002 | 198 | 198 | 0 |
| WG_IAD68410 | ***TSC1*** | chr9 | 135776097 | 135776229 | 133 | 133 | 0 |
| WG_IAD68410 | ***TSC1*** | chr9 | 135776971 | 135777091 | 121 | 121 | 0 |
| WG_IAD68410 | ***TSC1*** | chr9 | 135777987 | 135778179 | 193 | 193 | 0 |
| WG_IAD68410 | ***TSC1*** | chr9 | 135779033 | 135779209 | 177 | 177 | 0 |
| WG_IAD68410 | ***TSC1*** | chr9 | 135779793 | 135779846 | 54 | 54 | 0 |
| WG_IAD68410 | ***TSC1*** | chr9 | 135780963 | 135781531 | 569 | 569 | 0 |
| WG_IAD68410 | ***TSC1*** | chr9 | 135782113 | 135782227 | 115 | 115 | 0 |
| WG_IAD68410 | ***TSC1*** | chr9 | 135782683 | 135782762 | 80 | 80 | 0 |
| WG_IAD68410 | ***TSC1*** | chr9 | 135785953 | 135786084 | 132 | 132 | 0 |
| WG_IAD68410 | ***TSC1*** | chr9 | 135786384 | 135786505 | 122 | 122 | 0 |
| WG_IAD68410 | ***TSC1*** | chr9 | 135786835 | 135786960 | 126 | 126 | 0 |
| WG_IAD68410 | ***TSC1*** | chr9 | 135787664 | 135787849 | 186 | 186 | 0 |
| WG_IAD68410 | ***TSC1*** | chr9 | 135796745 | 135796828 | 84 | 84 | 0 |
| WG_IAD68410 | ***TSC1*** | chr9 | 135797201 | 135797365 | 165 | 165 | 0 |
| WG_IAD68410 | ***TSC1*** | chr9 | 135798730 | 135798884 | 155 | 155 | 0 |
| WG_IAD68410 | ***TSC1*** | chr9 | 135800969 | 135801131 | 163 | 163 | 0 |
| WG_IAD68410 | ***TSC1*** | chr9 | 135802583 | 135802696 | 114 | 114 | 0 |
| WG_IAD68410 | ***TSC1*** | chr9 | 135804149 | 135804264 | 116 | 116 | 0 |
| WG_IAD68410 | ***JAK2_14*** | chr9 | 5073698 | 5073785 | 88 | 88 | 0 |
| WG_IAD68410 | ***CDKN2A_2*** | chr9 | 21970901 | 21971207 | 307 | 307 | 0 |
| WG_IAD68410 | ***GNAQ_5*** | chr9 | 80409379 | 80409508 | 130 | 130 | 0 |
| WG_IAD68410 | ***GNAQ_4*** | chr9 | 80412436 | 80412564 | 129 | 129 | 0 |
| WG_IAD68410 | ***PPP6C_8*** | chr9 | 127911960 | 127912200 | 241 | 241 | 0 |
| WG_IAD68410 | ***PPP6C_7*** | chr9 | 127915812 | 127916021 | 210 | 210 | 0 |
| WG_IAD68410 | ***PPP6C_6*** | chr9 | 127916185 | 127916264 | 80 | 80 | 0 |
| WG_IAD68410 | ***PPP6C_5*** | chr9 | 127920520 | 127920661 | 142 | 142 | 0 |
| WG_IAD68410 | ***PPP6C_4*** | chr9 | 127923120 | 127923185 | 66 | 66 | 0 |
| WG_IAD68410 | ***ABL1_4*** | chr9 | 133738150 | 133738422 | 273 | 273 | 0 |
| WG_IAD68410 | ***ABL1_5*** | chr9 | 133747516 | 133747600 | 85 | 85 | 0 |
| WG_IAD68410 | ***ABL1_6*** | chr9 | 133748247 | 133748424 | 178 | 178 | 0 |
| WG_IAD68410 | ***ABL1_7*** | chr9 | 133750255 | 133750439 | 185 | 185 | 0 |
| WG_IAD68410 | ***NOTCH1_34*** | chr9 | 139390570 | 139392010 | 1441 | 1441 | 0 |
| WG_IAD68410 | ***NOTCH1_27*** | chr9 | 139397634 | 139397782 | 149 | 149 | 0 |
| WG_IAD68410 | ***NOTCH1_26*** | chr9 | 139399125 | 139399556 | 432 | 432 | 0 |
| WG_IAD68410 | ***GATA3*** | chr10 | 8097614 | 8097864 | 251 | 251 | 0 |
| WG_IAD68410 | ***GATA3*** | chr10 | 8100263 | 8100809 | 547 | 547 | 0 |
| WG_IAD68410 | ***GATA3*** | chr10 | 8105951 | 8106106 | 156 | 156 | 0 |
| WG_IAD68410 | ***GATA3*** | chr10 | 8111431 | 8111566 | 136 | 136 | 0 |
| WG_IAD68410 | ***GATA3*** | chr10 | 8115697 | 8115991 | 295 | 295 | 0 |
| WG_IAD68410 | ***RET_10*** | chr10 | 43609004 | 43609123 | 120 | 120 | 0 |
| WG_IAD68410 | ***RET_11*** | chr10 | 43609928 | 43610184 | 257 | 257 | 0 |
| WG_IAD68410 | ***RET_13*** | chr10 | 43613821 | 43613928 | 108 | 108 | 0 |
| WG_IAD68410 | ***RET_15*** | chr10 | 43615529 | 43615651 | 123 | 123 | 0 |
| WG_IAD68410 | ***RET_16*** | chr10 | 43617394 | 43617464 | 71 | 71 | 0 |
| WG_IAD68410 | ***PTEN_1*** | chr10 | 89624208 | 89624305 | 98 | 98 | 0 |
| WG_IAD68410 | ***PTEN_3*** | chr10 | 89685270 | 89685314 | 45 | 45 | 0 |
| WG_IAD68410 | ***PTEN_5*** | chr10 | 89692770 | 89693008 | 239 | 239 | 0 |
| WG_IAD68410 | ***PTEN_6*** | chr10 | 89711875 | 89712016 | 142 | 142 | 0 |
| WG_IAD68410 | ***PTEN_7*** | chr10 | 89717610 | 89717776 | 167 | 167 | 0 |
| WG_IAD68410 | ***PTEN_8*** | chr10 | 89720651 | 89720875 | 222 | 225 | 3 |
| WG_IAD68410 | ***PTEN_9*** | chr10 | 89725044 | 89725224 | 153 | 181 | 28 |
| WG_IAD68410 | ***FGFR2_15*** | chr10 | 123246868 | 123246938 | 71 | 71 | 0 |
| WG_IAD68410 | ***FGFR2_14*** | chr10 | 123247505 | 123247627 | 123 | 123 | 0 |
| WG_IAD68410 | ***FGFR2_13*** | chr10 | 123256046 | 123256236 | 191 | 191 | 0 |
| WG_IAD68410 | ***FGFR2_12*** | chr10 | 123258009 | 123258119 | 111 | 111 | 0 |
| WG_IAD68410 | ***FGFR2_11*** | chr10 | 123260340 | 123260461 | 122 | 122 | 0 |
| WG_IAD68410 | ***FGFR2_10*** | chr10 | 123263304 | 123263455 | 152 | 152 | 0 |
| WG_IAD68410 | ***FGFR2_9*** | chr10 | 123274631 | 123274833 | 203 | 203 | 0 |
| WG_IAD68410 | ***FGFR2_8*** | chr10 | 123276833 | 123276977 | 145 | 145 | 0 |
| WG_IAD68410 | ***FGFR2_7*** | chr10 | 123279493 | 123279683 | 191 | 191 | 0 |
| WG_IAD68410 | ***MEN1*** | chr11 | 64571801 | 64572293 | 493 | 493 | 0 |
| WG_IAD68410 | ***MEN1*** | chr11 | 64572501 | 64572675 | 175 | 175 | 0 |
| WG_IAD68410 | ***MEN1*** | chr11 | 64573102 | 64573247 | 146 | 146 | 0 |
| WG_IAD68410 | ***MEN1*** | chr11 | 64573699 | 64573845 | 147 | 147 | 0 |
| WG_IAD68410 | ***MEN1*** | chr11 | 64574478 | 64574575 | 98 | 98 | 0 |
| WG_IAD68410 | ***MEN1*** | chr11 | 64574646 | 64574696 | 51 | 51 | 0 |
| WG_IAD68410 | ***MEN1*** | chr11 | 64575019 | 64575157 | 139 | 139 | 0 |
| WG_IAD68410 | ***MEN1*** | chr11 | 64575358 | 64575576 | 219 | 219 | 0 |
| WG_IAD68410 | ***MEN1*** | chr11 | 64577117 | 64577586 | 470 | 470 | 0 |
| WG_IAD68410 | ***SDHD*** | chr11 | 111957627 | 111957688 | 62 | 62 | 0 |
| WG_IAD68410 | ***SDHD*** | chr11 | 111958576 | 111958702 | 127 | 127 | 0 |
| WG_IAD68410 | ***SDHD*** | chr11 | 111959586 | 111959740 | 155 | 155 | 0 |
| WG_IAD68410 | ***SDHD*** | chr11 | 111963799 | 111963926 | 0 | 128 | 128 |
| WG_IAD68410 | ***SDHD*** | chr11 | 111965524 | 111965699 | 85 | 176 | 91 |
| WG_IAD68410 | ***WT1*** | chr11 | 32410599 | 32410730 | 132 | 132 | 0 |
| WG_IAD68410 | ***WT1*** | chr11 | 32413513 | 32413615 | 103 | 103 | 0 |
| WG_IAD68410 | ***WT1*** | chr11 | 32414207 | 32414306 | 100 | 100 | 0 |
| WG_IAD68410 | ***WT1*** | chr11 | 32417798 | 32417958 | 161 | 161 | 0 |
| WG_IAD68410 | ***WT1*** | chr11 | 32421489 | 32421595 | 107 | 107 | 0 |
| WG_IAD68410 | ***WT1*** | chr11 | 32438031 | 32438091 | 61 | 61 | 0 |
| WG_IAD68410 | ***WT1*** | chr11 | 32439118 | 32439205 | 88 | 88 | 0 |
| WG_IAD68410 | ***WT1*** | chr11 | 32449497 | 32449609 | 113 | 113 | 0 |
| WG_IAD68410 | ***WT1*** | chr11 | 32450038 | 32450170 | 133 | 133 | 0 |
| WG_IAD68410 | ***WT1*** | chr11 | 32452071 | 32452090 | 20 | 20 | 0 |
| WG_IAD68410 | ***WT1*** | chr11 | 32456241 | 32456896 | 590 | 656 | 66 |
| WG_IAD68410 | ***HRAS_4*** | chr11 | 533453 | 533612 | 160 | 160 | 0 |
| WG_IAD68410 | ***HRAS_3*** | chr11 | 533766 | 533944 | 179 | 179 | 0 |
| WG_IAD68410 | ***HRAS_2*** | chr11 | 534212 | 534375 | 164 | 164 | 0 |
| WG_IAD68410 | ***ATM_8*** | chr11 | 108117691 | 108117854 | 164 | 164 | 0 |
| WG_IAD68410 | ***ATM_9*** | chr11 | 108119660 | 108119829 | 170 | 170 | 0 |
| WG_IAD68410 | ***ATM_12*** | chr11 | 108123544 | 108123639 | 96 | 96 | 0 |
| WG_IAD68410 | ***ATM_17*** | chr11 | 108137898 | 108138069 | 172 | 172 | 0 |
| WG_IAD68410 | ***ATM_26*** | chr11 | 108154954 | 108155200 | 221 | 247 | 26 |
| WG_IAD68410 | ***ATM_34*** | chr11 | 108170441 | 108170612 | 172 | 172 | 0 |
| WG_IAD68410 | ***ATM_35*** | chr11 | 108172375 | 108172516 | 142 | 142 | 0 |
| WG_IAD68410 | ***ATM_36*** | chr11 | 108173580 | 108173756 | 177 | 177 | 0 |
| WG_IAD68410 | ***ATM_39*** | chr11 | 108180887 | 108181042 | 156 | 156 | 0 |
| WG_IAD68410 | ***ATM_50*** | chr11 | 108200941 | 108201148 | 208 | 208 | 0 |
| WG_IAD68410 | ***ATM_54*** | chr11 | 108204613 | 108204695 | 83 | 83 | 0 |
| WG_IAD68410 | ***ATM_55*** | chr11 | 108205696 | 108205836 | 141 | 141 | 0 |
| WG_IAD68410 | ***ATM_56*** | chr11 | 108206572 | 108206688 | 117 | 117 | 0 |
| WG_IAD68410 | ***ATM_59*** | chr11 | 108218006 | 108218092 | 87 | 87 | 0 |
| WG_IAD68410 | ***ATM_61*** | chr11 | 108225538 | 108225601 | 64 | 64 | 0 |
| WG_IAD68410 | ***ATM_63*** | chr11 | 108236052 | 108236232 | 181 | 181 | 0 |
| WG_IAD68410 | ***CDK4*** | chr12 | 58142303 | 58142405 | 103 | 103 | 0 |
| WG_IAD68410 | ***CDK4*** | chr12 | 58142960 | 58143105 | 146 | 146 | 0 |
| WG_IAD68410 | ***CDK4*** | chr12 | 58143232 | 58143292 | 61 | 61 | 0 |
| WG_IAD68410 | ***CDK4*** | chr12 | 58144434 | 58144553 | 120 | 120 | 0 |
| WG_IAD68410 | ***CDK4*** | chr12 | 58144701 | 58144878 | 178 | 178 | 0 |
| WG_IAD68410 | ***CDK4*** | chr12 | 58144985 | 58145130 | 146 | 146 | 0 |
| WG_IAD68410 | ***CDK4*** | chr12 | 58145278 | 58145505 | 228 | 228 | 0 |
| WG_IAD68410 | ***TBX3*** | chr12 | 115109641 | 115110112 | 472 | 472 | 0 |
| WG_IAD68410 | ***TBX3*** | chr12 | 115111965 | 115112645 | 681 | 681 | 0 |
| WG_IAD68410 | ***TBX3*** | chr12 | 115114113 | 115114280 | 168 | 168 | 0 |
| WG_IAD68410 | ***TBX3*** | chr12 | 115115380 | 115115466 | 87 | 87 | 0 |
| WG_IAD68410 | ***TBX3*** | chr12 | 115117305 | 115117461 | 157 | 157 | 0 |
| WG_IAD68410 | ***TBX3*** | chr12 | 115117713 | 115117782 | 70 | 70 | 0 |
| WG_IAD68410 | ***TBX3*** | chr12 | 115118679 | 115118956 | 278 | 278 | 0 |
| WG_IAD68410 | ***TBX3*** | chr12 | 115120612 | 115121010 | 399 | 399 | 0 |
| WG_IAD68410 | ***KRAS_4*** | chr12 | 25378548 | 25378707 | 160 | 160 | 0 |
| WG_IAD68410 | ***KRAS_3*** | chr12 | 25380168 | 25380346 | 179 | 179 | 0 |
| WG_IAD68410 | ***KRAS_2*** | chr12 | 25398208 | 25398329 | 122 | 122 | 0 |
| WG_IAD68410 | ***ARID2_15*** | chr12 | 46243819 | 46246679 | 2861 | 2861 | 0 |
| WG_IAD68410 | ***PTPN11_3*** | chr12 | 112888122 | 112888316 | 195 | 195 | 0 |
| WG_IAD68410 | ***PTPN11_13*** | chr12 | 112926828 | 112926979 | 152 | 152 | 0 |
| WG_IAD68410 | ***HNF1A_3*** | chr12 | 121431323 | 121431509 | 187 | 187 | 0 |
| WG_IAD68410 | ***HNF1A_4*** | chr12 | 121431967 | 121432208 | 242 | 242 | 0 |
| WG_IAD68410 | ***FLT1*** | chr13 | 28877299 | 28877510 | 212 | 212 | 0 |
| WG_IAD68410 | ***FLT1*** | chr13 | 28880810 | 28880914 | 105 | 105 | 0 |
| WG_IAD68410 | ***FLT1*** | chr13 | 28882975 | 28883069 | 95 | 95 | 0 |
| WG_IAD68410 | ***FLT1*** | chr13 | 28885722 | 28885874 | 153 | 153 | 0 |
| WG_IAD68410 | ***FLT1*** | chr13 | 28886125 | 28886240 | 116 | 116 | 0 |
| WG_IAD68410 | ***FLT1*** | chr13 | 28891630 | 28891739 | 110 | 110 | 0 |
| WG_IAD68410 | ***FLT1*** | chr13 | 28893555 | 28893676 | 122 | 122 | 0 |
| WG_IAD68410 | ***FLT1*** | chr13 | 28895595 | 28895727 | 133 | 133 | 0 |
| WG_IAD68410 | ***FLT1*** | chr13 | 28896394 | 28896501 | 108 | 108 | 0 |
| WG_IAD68410 | ***FLT1*** | chr13 | 28896922 | 28897088 | 167 | 167 | 0 |
| WG_IAD68410 | ***FLT1*** | chr13 | 28901594 | 28901692 | 99 | 99 | 0 |
| WG_IAD68410 | ***FLT1*** | chr13 | 28903747 | 28903870 | 124 | 124 | 0 |
| WG_IAD68410 | ***FLT1*** | chr13 | 28908157 | 28908271 | 115 | 115 | 0 |
| WG_IAD68410 | ***FLT1*** | chr13 | 28913300 | 28913442 | 143 | 143 | 0 |
| WG_IAD68410 | ***FLT1*** | chr13 | 28919577 | 28919693 | 117 | 117 | 0 |
| WG_IAD68410 | ***FLT1*** | chr13 | 28931686 | 28931827 | 142 | 142 | 0 |
| WG_IAD68410 | ***FLT1*** | chr13 | 28942710 | 28942805 | 96 | 96 | 0 |
| WG_IAD68410 | ***FLT1*** | chr13 | 28959017 | 28959173 | 157 | 157 | 0 |
| WG_IAD68410 | ***FLT1*** | chr13 | 28963833 | 28964246 | 414 | 414 | 0 |
| WG_IAD68410 | ***FLT1*** | chr13 | 28971092 | 28971210 | 119 | 119 | 0 |
| WG_IAD68410 | ***FLT1*** | chr13 | 28973176 | 28973260 | 85 | 85 | 0 |
| WG_IAD68410 | ***FLT1*** | chr13 | 28979912 | 28980036 | 125 | 125 | 0 |
| WG_IAD68410 | ***FLT1*** | chr13 | 29001291 | 29001460 | 170 | 170 | 0 |
| WG_IAD68410 | ***FLT1*** | chr13 | 29001884 | 29002063 | 180 | 180 | 0 |
| WG_IAD68410 | ***FLT1*** | chr13 | 29004182 | 29004309 | 128 | 128 | 0 |
| WG_IAD68410 | ***FLT1*** | chr13 | 29005268 | 29005452 | 185 | 185 | 0 |
| WG_IAD68410 | ***FLT1*** | chr13 | 29007951 | 29008097 | 147 | 147 | 0 |
| WG_IAD68410 | ***FLT1*** | chr13 | 29008190 | 29008362 | 173 | 173 | 0 |
| WG_IAD68410 | ***FLT1*** | chr13 | 29012353 | 29012487 | 135 | 135 | 0 |
| WG_IAD68410 | ***FLT1*** | chr13 | 29041035 | 29041271 | 237 | 237 | 0 |
| WG_IAD68410 | ***FLT1*** | chr13 | 29041653 | 29041759 | 107 | 107 | 0 |
| WG_IAD68410 | ***FLT1*** | chr13 | 29068912 | 29068985 | 74 | 74 | 0 |
| WG_IAD68410 | ***FLT3_20*** | chr13 | 28592604 | 28592726 | 123 | 123 | 0 |
| WG_IAD68410 | ***FLT3_16*** | chr13 | 28602315 | 28602425 | 111 | 111 | 0 |
| WG_IAD68410 | ***FLT3_14*** | chr13 | 28608219 | 28608351 | 133 | 133 | 0 |
| WG_IAD68410 | ***FLT3_11*** | chr13 | 28610072 | 28610180 | 109 | 109 | 0 |
| WG_IAD68410 | ***RB1_4*** | chr13 | 48919216 | 48919335 | 120 | 120 | 0 |
| WG_IAD68410 | ***RB1_6*** | chr13 | 48923092 | 48923159 | 68 | 68 | 0 |
| WG_IAD68410 | ***RB1_10*** | chr13 | 48941630 | 48941739 | 110 | 110 | 0 |
| WG_IAD68410 | ***RB1_11*** | chr13 | 48942663 | 48942740 | 78 | 78 | 0 |
| WG_IAD68410 | ***RB1_14*** | chr13 | 48953730 | 48953786 | 57 | 57 | 0 |
| WG_IAD68410 | ***RB1_17*** | chr13 | 48955383 | 48955579 | 197 | 197 | 0 |
| WG_IAD68410 | ***RB1_18*** | chr13 | 49027129 | 49027247 | 119 | 119 | 0 |
| WG_IAD68410 | ***RB1_20*** | chr13 | 49033824 | 49033969 | 146 | 146 | 0 |
| WG_IAD68410 | ***RB1_21*** | chr13 | 49037867 | 49037971 | 105 | 105 | 0 |
| WG_IAD68410 | ***RB1_22*** | chr13 | 49039134 | 49039247 | 104 | 114 | 10 |
| WG_IAD68410 | ***FANCM*** | chr14 | 45605230 | 45605747 | 518 | 518 | 0 |
| WG_IAD68410 | ***FANCM*** | chr14 | 45606267 | 45606449 | 183 | 183 | 0 |
| WG_IAD68410 | ***FANCM*** | chr14 | 45609830 | 45609917 | 88 | 88 | 0 |
| WG_IAD68410 | ***FANCM*** | chr14 | 45618035 | 45618203 | 169 | 169 | 0 |
| WG_IAD68410 | ***FANCM*** | chr14 | 45620595 | 45620736 | 142 | 142 | 0 |
| WG_IAD68410 | ***FANCM*** | chr14 | 45623118 | 45623260 | 143 | 143 | 0 |
| WG_IAD68410 | ***FANCM*** | chr14 | 45623895 | 45624030 | 136 | 136 | 0 |
| WG_IAD68410 | ***FANCM*** | chr14 | 45624571 | 45624667 | 97 | 97 | 0 |
| WG_IAD68410 | ***FANCM*** | chr14 | 45628294 | 45628488 | 195 | 195 | 0 |
| WG_IAD68410 | ***FANCM*** | chr14 | 45633557 | 45633773 | 217 | 217 | 0 |
| WG_IAD68410 | ***FANCM*** | chr14 | 45636148 | 45636371 | 212 | 224 | 12 |
| WG_IAD68410 | ***FANCM*** | chr14 | 45639787 | 45639954 | 168 | 168 | 0 |
| WG_IAD68410 | ***FANCM*** | chr14 | 45642253 | 45642418 | 166 | 166 | 0 |
| WG_IAD68410 | ***FANCM*** | chr14 | 45644269 | 45646184 | 1885 | 1916 | 31 |
| WG_IAD68410 | ***FANCM*** | chr14 | 45650628 | 45650732 | 105 | 105 | 0 |
| WG_IAD68410 | ***FANCM*** | chr14 | 45650835 | 45650913 | 79 | 79 | 0 |
| WG_IAD68410 | ***FANCM*** | chr14 | 45652972 | 45653110 | 139 | 139 | 0 |
| WG_IAD68410 | ***FANCM*** | chr14 | 45654415 | 45654581 | 167 | 167 | 0 |
| WG_IAD68410 | ***FANCM*** | chr14 | 45656979 | 45657095 | 117 | 117 | 0 |
| WG_IAD68410 | ***FANCM*** | chr14 | 45658000 | 45658570 | 571 | 571 | 0 |
| WG_IAD68410 | ***FANCM*** | chr14 | 45665370 | 45665755 | 386 | 386 | 0 |
| WG_IAD68410 | ***FANCM*** | chr14 | 45667842 | 45668143 | 302 | 302 | 0 |
| WG_IAD68410 | ***FANCM*** | chr14 | 45669068 | 45669216 | 149 | 149 | 0 |
| WG_IAD68410 | ***FOXA1*** | chr14 | 38060565 | 38061921 | 1357 | 1357 | 0 |
| WG_IAD68410 | ***FOXA1*** | chr14 | 38064101 | 38064182 | 82 | 82 | 0 |
| WG_IAD68410 | ***AKT_5*** | chr14 | 105241413 | 105241544 | 132 | 132 | 0 |
| WG_IAD68410 | ***AKT_2*** | chr14 | 105246425 | 105246553 | 129 | 129 | 0 |
| WG_IAD68410 | ***BLM*** | chr15 | 91290618 | 91290725 | 108 | 108 | 0 |
| WG_IAD68410 | ***BLM*** | chr15 | 91292592 | 91293302 | 711 | 711 | 0 |
| WG_IAD68410 | ***BLM*** | chr15 | 91295012 | 91295181 | 170 | 170 | 0 |
| WG_IAD68410 | ***BLM*** | chr15 | 91298036 | 91298173 | 138 | 138 | 0 |
| WG_IAD68410 | ***BLM*** | chr15 | 91303372 | 91303514 | 143 | 143 | 0 |
| WG_IAD68410 | ***BLM*** | chr15 | 91303819 | 91304490 | 672 | 672 | 0 |
| WG_IAD68410 | ***BLM*** | chr15 | 91306191 | 91306392 | 202 | 202 | 0 |
| WG_IAD68410 | ***BLM*** | chr15 | 91308521 | 91308649 | 129 | 129 | 0 |
| WG_IAD68410 | ***BLM*** | chr15 | 91310135 | 91310258 | 124 | 124 | 0 |
| WG_IAD68410 | ***BLM*** | chr15 | 91312358 | 91312466 | 109 | 109 | 0 |
| WG_IAD68410 | ***BLM*** | chr15 | 91312663 | 91312821 | 159 | 159 | 0 |
| WG_IAD68410 | ***BLM*** | chr15 | 91326047 | 91326163 | 117 | 117 | 0 |
| WG_IAD68410 | ***BLM*** | chr15 | 91328146 | 91328316 | 171 | 171 | 0 |
| WG_IAD68410 | ***BLM*** | chr15 | 91333874 | 91334079 | 206 | 206 | 0 |
| WG_IAD68410 | ***BLM*** | chr15 | 91337392 | 91337592 | 201 | 201 | 0 |
| WG_IAD68410 | ***BLM*** | chr15 | 91341415 | 91341572 | 158 | 158 | 0 |
| WG_IAD68410 | ***BLM*** | chr15 | 91346746 | 91346955 | 210 | 210 | 0 |
| WG_IAD68410 | ***BLM*** | chr15 | 91347392 | 91347594 | 203 | 203 | 0 |
| WG_IAD68410 | ***BLM*** | chr15 | 91352362 | 91352494 | 133 | 133 | 0 |
| WG_IAD68410 | ***BLM*** | chr15 | 91354430 | 91354641 | 212 | 212 | 0 |
| WG_IAD68410 | ***BLM*** | chr15 | 91358327 | 91358514 | 188 | 188 | 0 |
| WG_IAD68410 | ***IGF1R*** | chr15 | 99192806 | 99192909 | 96 | 104 | 8 |
| WG_IAD68410 | ***IGF1R*** | chr15 | 99250786 | 99251341 | 556 | 556 | 0 |
| WG_IAD68410 | ***IGF1R*** | chr15 | 99434549 | 99434871 | 323 | 323 | 0 |
| WG_IAD68410 | ***IGF1R*** | chr15 | 99439981 | 99440139 | 159 | 159 | 0 |
| WG_IAD68410 | ***IGF1R*** | chr15 | 99442701 | 99442855 | 155 | 155 | 0 |
| WG_IAD68410 | ***IGF1R*** | chr15 | 99451909 | 99452133 | 225 | 225 | 0 |
| WG_IAD68410 | ***IGF1R*** | chr15 | 99454539 | 99454675 | 137 | 137 | 0 |
| WG_IAD68410 | ***IGF1R*** | chr15 | 99456268 | 99456516 | 249 | 249 | 0 |
| WG_IAD68410 | ***IGF1R*** | chr15 | 99459188 | 99459365 | 178 | 178 | 0 |
| WG_IAD68410 | ***IGF1R*** | chr15 | 99459896 | 99460110 | 215 | 215 | 0 |
| WG_IAD68410 | ***IGF1R*** | chr15 | 99465372 | 99465665 | 294 | 294 | 0 |
| WG_IAD68410 | ***IGF1R*** | chr15 | 99467100 | 99467246 | 147 | 147 | 0 |
| WG_IAD68410 | ***IGF1R*** | chr15 | 99467749 | 99467918 | 170 | 170 | 0 |
| WG_IAD68410 | ***IGF1R*** | chr15 | 99472782 | 99472894 | 113 | 113 | 0 |
| WG_IAD68410 | ***IGF1R*** | chr15 | 99473459 | 99473539 | 81 | 81 | 0 |
| WG_IAD68410 | ***IGF1R*** | chr15 | 99478048 | 99478287 | 240 | 240 | 0 |
| WG_IAD68410 | ***IGF1R*** | chr15 | 99478540 | 99478660 | 121 | 121 | 0 |
| WG_IAD68410 | ***IGF1R*** | chr15 | 99482425 | 99482594 | 170 | 170 | 0 |
| WG_IAD68410 | ***IGF1R*** | chr15 | 99486147 | 99486286 | 140 | 140 | 0 |
| WG_IAD68410 | ***IGF1R*** | chr15 | 99491798 | 99491942 | 145 | 145 | 0 |
| WG_IAD68410 | ***IGF1R*** | chr15 | 99500285 | 99500676 | 392 | 392 | 0 |
| WG_IAD68410 | ***SMAD3*** | chr15 | 67358488 | 67358703 | 216 | 216 | 0 |
| WG_IAD68410 | ***SMAD3*** | chr15 | 67430360 | 67430443 | 84 | 84 | 0 |
| WG_IAD68410 | ***SMAD3*** | chr15 | 67457228 | 67457431 | 204 | 204 | 0 |
| WG_IAD68410 | ***SMAD3*** | chr15 | 67457586 | 67457727 | 142 | 142 | 0 |
| WG_IAD68410 | ***SMAD3*** | chr15 | 67459112 | 67459196 | 85 | 85 | 0 |
| WG_IAD68410 | ***SMAD3*** | chr15 | 67462887 | 67462947 | 61 | 61 | 0 |
| WG_IAD68410 | ***SMAD3*** | chr15 | 67473574 | 67473796 | 223 | 223 | 0 |
| WG_IAD68410 | ***SMAD3*** | chr15 | 67477060 | 67477207 | 148 | 148 | 0 |
| WG_IAD68410 | ***SMAD3*** | chr15 | 67479698 | 67479852 | 155 | 155 | 0 |
| WG_IAD68410 | ***SMAD3*** | chr15 | 67482746 | 67482879 | 134 | 134 | 0 |
| WG_IAD68410 | ***MEK1_2*** | chr15 | 66727365 | 66727575 | 211 | 211 | 0 |
| WG_IAD68410 | ***MEK1_3*** | chr15 | 66729084 | 66729230 | 147 | 147 | 0 |
| WG_IAD68410 | ***MEK1_4*** | chr15 | 66735618 | 66735695 | 78 | 78 | 0 |
| WG_IAD68410 | ***MEK1_5*** | chr15 | 66736994 | 66737045 | 52 | 52 | 0 |
| WG_IAD68410 | ***MEK1_6*** | chr15 | 66774093 | 66774217 | 125 | 125 | 0 |
| WG_IAD68410 | ***MEK1_7*** | chr15 | 66777328 | 66777529 | 202 | 202 | 0 |
| WG_IAD68410 | ***MEK1_8*** | chr15 | 66779566 | 66779630 | 65 | 65 | 0 |
| WG_IAD68410 | ***MEK1_9*** | chr15 | 66781553 | 66781614 | 62 | 62 | 0 |
| WG_IAD68410 | ***MEK1_10*** | chr15 | 66782056 | 66782101 | 46 | 46 | 0 |
| WG_IAD68410 | ***MEK1_11*** | chr15 | 66782840 | 66782959 | 120 | 120 | 0 |
| WG_IAD68410 | ***IDH2_4*** | chr15 | 90631819 | 90631979 | 161 | 161 | 0 |
| WG_IAD68410 | ***TSC2*** | chr16 | 2098612 | 2098759 | 148 | 148 | 0 |
| WG_IAD68410 | ***TSC2*** | chr16 | 2100396 | 2100492 | 97 | 97 | 0 |
| WG_IAD68410 | ***TSC2*** | chr16 | 2103338 | 2103458 | 121 | 121 | 0 |
| WG_IAD68410 | ***TSC2*** | chr16 | 2104292 | 2104446 | 155 | 155 | 0 |
| WG_IAD68410 | ***TSC2*** | chr16 | 2105398 | 2105525 | 128 | 128 | 0 |
| WG_IAD68410 | ***TSC2*** | chr16 | 2106192 | 2106250 | 59 | 59 | 0 |
| WG_IAD68410 | ***TSC2*** | chr16 | 2106640 | 2106775 | 136 | 136 | 0 |
| WG_IAD68410 | ***TSC2*** | chr16 | 2107101 | 2107184 | 84 | 84 | 0 |
| WG_IAD68410 | ***TSC2*** | chr16 | 2108743 | 2108879 | 137 | 137 | 0 |
| WG_IAD68410 | ***TSC2*** | chr16 | 2110666 | 2110819 | 154 | 154 | 0 |
| WG_IAD68410 | ***TSC2*** | chr16 | 2111867 | 2112014 | 148 | 148 | 0 |
| WG_IAD68410 | ***TSC2*** | chr16 | 2112493 | 2112606 | 114 | 114 | 0 |
| WG_IAD68410 | ***TSC2*** | chr16 | 2112968 | 2113059 | 92 | 92 | 0 |
| WG_IAD68410 | ***TSC2*** | chr16 | 2114268 | 2114433 | 166 | 166 | 0 |
| WG_IAD68410 | ***TSC2*** | chr16 | 2115515 | 2115641 | 127 | 127 | 0 |
| WG_IAD68410 | ***TSC2*** | chr16 | 2120452 | 2120584 | 133 | 133 | 0 |
| WG_IAD68410 | ***TSC2*** | chr16 | 2121506 | 2121622 | 117 | 117 | 0 |
| WG_IAD68410 | ***TSC2*** | chr16 | 2121780 | 2121940 | 161 | 161 | 0 |
| WG_IAD68410 | ***TSC2*** | chr16 | 2122237 | 2122369 | 133 | 133 | 0 |
| WG_IAD68410 | ***TSC2*** | chr16 | 2122845 | 2122989 | 145 | 145 | 0 |
| WG_IAD68410 | ***TSC2*** | chr16 | 2124196 | 2124395 | 200 | 200 | 0 |
| WG_IAD68410 | ***TSC2*** | chr16 | 2125795 | 2125898 | 104 | 104 | 0 |
| WG_IAD68410 | ***TSC2*** | chr16 | 2126064 | 2126176 | 113 | 113 | 0 |
| WG_IAD68410 | ***TSC2*** | chr16 | 2126487 | 2126591 | 105 | 105 | 0 |
| WG_IAD68410 | ***TSC2*** | chr16 | 2127594 | 2127732 | 139 | 139 | 0 |
| WG_IAD68410 | ***TSC2*** | chr16 | 2129028 | 2129202 | 175 | 175 | 0 |
| WG_IAD68410 | ***TSC2*** | chr16 | 2129272 | 2129434 | 163 | 163 | 0 |
| WG_IAD68410 | ***TSC2*** | chr16 | 2129553 | 2129675 | 123 | 123 | 0 |
| WG_IAD68410 | ***TSC2*** | chr16 | 2130161 | 2130383 | 223 | 223 | 0 |
| WG_IAD68410 | ***TSC2*** | chr16 | 2131591 | 2131804 | 214 | 214 | 0 |
| WG_IAD68410 | ***TSC2*** | chr16 | 2132432 | 2132510 | 79 | 79 | 0 |
| WG_IAD68410 | ***TSC2*** | chr16 | 2133691 | 2133822 | 132 | 132 | 0 |
| WG_IAD68410 | ***TSC2*** | chr16 | 2134224 | 2134721 | 498 | 498 | 0 |
| WG_IAD68410 | ***TSC2*** | chr16 | 2134947 | 2135032 | 86 | 86 | 0 |
| WG_IAD68410 | ***TSC2*** | chr16 | 2135226 | 2135328 | 103 | 103 | 0 |
| WG_IAD68410 | ***TSC2*** | chr16 | 2136189 | 2136385 | 197 | 197 | 0 |
| WG_IAD68410 | ***TSC2*** | chr16 | 2136728 | 2136877 | 150 | 150 | 0 |
| WG_IAD68410 | ***TSC2*** | chr16 | 2137859 | 2137947 | 89 | 89 | 0 |
| WG_IAD68410 | ***TSC2*** | chr16 | 2138044 | 2138145 | 102 | 102 | 0 |
| WG_IAD68410 | ***TSC2*** | chr16 | 2138223 | 2138331 | 109 | 109 | 0 |
| WG_IAD68410 | ***TSC2*** | chr16 | 2138442 | 2138616 | 175 | 175 | 0 |
| WG_IAD68410 | ***CDH1_3*** | chr16 | 68835573 | 68835796 | 224 | 224 | 0 |
| WG_IAD68410 | ***CDH1_8*** | chr16 | 68846038 | 68846166 | 129 | 129 | 0 |
| WG_IAD68410 | ***CDH1_9*** | chr16 | 68847216 | 68847398 | 183 | 183 | 0 |
| WG_IAD68410 | ***NF1*** | chr17 | 29422323 | 29422392 | 70 | 70 | 0 |
| WG_IAD68410 | ***NF1*** | chr17 | 29482996 | 29483149 | 154 | 154 | 0 |
| WG_IAD68410 | ***NF1*** | chr17 | 29486023 | 29486116 | 94 | 94 | 0 |
| WG_IAD68410 | ***NF1*** | chr17 | 29490199 | 29490399 | 201 | 201 | 0 |
| WG_IAD68410 | ***NF1*** | chr17 | 29496904 | 29497020 | 117 | 117 | 0 |
| WG_IAD68410 | ***NF1*** | chr17 | 29508435 | 29508512 | 78 | 78 | 0 |
| WG_IAD68410 | ***NF1*** | chr17 | 29508723 | 29508808 | 86 | 86 | 0 |
| WG_IAD68410 | ***NF1*** | chr17 | 29509521 | 29509688 | 168 | 168 | 0 |
| WG_IAD68410 | ***NF1*** | chr17 | 29527435 | 29527618 | 184 | 184 | 0 |
| WG_IAD68410 | ***NF1*** | chr17 | 29528050 | 29528182 | 133 | 133 | 0 |
| WG_IAD68410 | ***NF1*** | chr17 | 29528424 | 29528508 | 85 | 85 | 0 |
| WG_IAD68410 | ***NF1*** | chr17 | 29533253 | 29533394 | 142 | 142 | 0 |
| WG_IAD68410 | ***NF1*** | chr17 | 29541464 | 29541608 | 145 | 145 | 0 |
| WG_IAD68410 | ***NF1*** | chr17 | 29546018 | 29546141 | 124 | 124 | 0 |
| WG_IAD68410 | ***NF1*** | chr17 | 29548863 | 29549013 | 151 | 151 | 0 |
| WG_IAD68410 | ***NF1*** | chr17 | 29550457 | 29550590 | 134 | 134 | 0 |
| WG_IAD68410 | ***NF1*** | chr17 | 29552108 | 29552273 | 166 | 166 | 0 |
| WG_IAD68410 | ***NF1*** | chr17 | 29553448 | 29553707 | 260 | 260 | 0 |
| WG_IAD68410 | ***NF1*** | chr17 | 29554231 | 29554314 | 84 | 84 | 0 |
| WG_IAD68410 | ***NF1*** | chr17 | 29554536 | 29554629 | 94 | 94 | 0 |
| WG_IAD68410 | ***NF1*** | chr17 | 29556038 | 29556488 | 451 | 451 | 0 |
| WG_IAD68410 | ***NF1*** | chr17 | 29556848 | 29556997 | 150 | 150 | 0 |
| WG_IAD68410 | ***NF1*** | chr17 | 29557273 | 29557405 | 133 | 133 | 0 |
| WG_IAD68410 | ***NF1*** | chr17 | 29557855 | 29557948 | 94 | 94 | 0 |
| WG_IAD68410 | ***NF1*** | chr17 | 29559086 | 29559212 | 107 | 127 | 20 |
| WG_IAD68410 | ***NF1*** | chr17 | 29559713 | 29559904 | 192 | 192 | 0 |
| WG_IAD68410 | ***NF1*** | chr17 | 29560015 | 29560236 | 222 | 222 | 0 |
| WG_IAD68410 | ***NF1*** | chr17 | 29562624 | 29562795 | 172 | 172 | 0 |
| WG_IAD68410 | ***NF1*** | chr17 | 29562931 | 29563044 | 114 | 114 | 0 |
| WG_IAD68410 | ***NF1*** | chr17 | 29575997 | 29576142 | 146 | 146 | 0 |
| WG_IAD68410 | ***NF1*** | chr17 | 29579951 | 29580023 | 73 | 73 | 0 |
| WG_IAD68410 | ***NF1*** | chr17 | 29585357 | 29585525 | 169 | 169 | 0 |
| WG_IAD68410 | ***NF1*** | chr17 | 29586045 | 29586152 | 108 | 108 | 0 |
| WG_IAD68410 | ***NF1*** | chr17 | 29587382 | 29587538 | 157 | 157 | 0 |
| WG_IAD68410 | ***NF1*** | chr17 | 29588724 | 29588880 | 157 | 157 | 0 |
| WG_IAD68410 | ***NF1*** | chr17 | 29592242 | 29592362 | 121 | 121 | 0 |
| WG_IAD68410 | ***NF1*** | chr17 | 29652833 | 29653275 | 443 | 443 | 0 |
| WG_IAD68410 | ***NF1*** | chr17 | 29654512 | 29654862 | 351 | 351 | 0 |
| WG_IAD68410 | ***NF1*** | chr17 | 29657309 | 29657521 | 213 | 213 | 0 |
| WG_IAD68410 | ***NF1*** | chr17 | 29661851 | 29662054 | 204 | 204 | 0 |
| WG_IAD68410 | ***NF1*** | chr17 | 29663346 | 29663496 | 151 | 151 | 0 |
| WG_IAD68410 | ***NF1*** | chr17 | 29663648 | 29663937 | 290 | 290 | 0 |
| WG_IAD68410 | ***NF1*** | chr17 | 29664381 | 29664605 | 225 | 225 | 0 |
| WG_IAD68410 | ***NF1*** | chr17 | 29664832 | 29664903 | 72 | 72 | 0 |
| WG_IAD68410 | ***NF1*** | chr17 | 29665038 | 29665162 | 125 | 125 | 0 |
| WG_IAD68410 | ***NF1*** | chr17 | 29665717 | 29665828 | 112 | 112 | 0 |
| WG_IAD68410 | ***NF1*** | chr17 | 29667518 | 29667668 | 151 | 151 | 0 |
| WG_IAD68410 | ***NF1*** | chr17 | 29670022 | 29670158 | 137 | 137 | 0 |
| WG_IAD68410 | ***NF1*** | chr17 | 29676133 | 29676274 | 142 | 142 | 0 |
| WG_IAD68410 | ***NF1*** | chr17 | 29677196 | 29677341 | 146 | 146 | 0 |
| WG_IAD68410 | ***NF1*** | chr17 | 29679270 | 29679437 | 168 | 168 | 0 |
| WG_IAD68410 | ***NF1*** | chr17 | 29683473 | 29683605 | 133 | 133 | 0 |
| WG_IAD68410 | ***NF1*** | chr17 | 29683973 | 29684113 | 141 | 141 | 0 |
| WG_IAD68410 | ***NF1*** | chr17 | 29684282 | 29684392 | 111 | 111 | 0 |
| WG_IAD68410 | ***NF1*** | chr17 | 29685493 | 29685645 | 153 | 153 | 0 |
| WG_IAD68410 | ***NF1*** | chr17 | 29685982 | 29686038 | 57 | 57 | 0 |
| WG_IAD68410 | ***NF1*** | chr17 | 29687500 | 29687726 | 227 | 227 | 0 |
| WG_IAD68410 | ***NF1*** | chr17 | 29701026 | 29701178 | 153 | 153 | 0 |
| WG_IAD68410 | ***SPOP*** | chr17 | 47677735 | 47677889 | 155 | 155 | 0 |
| WG_IAD68410 | ***SPOP*** | chr17 | 47679222 | 47679374 | 153 | 153 | 0 |
| WG_IAD68410 | ***SPOP*** | chr17 | 47684607 | 47684739 | 133 | 133 | 0 |
| WG_IAD68410 | ***SPOP*** | chr17 | 47685231 | 47685296 | 66 | 66 | 0 |
| WG_IAD68410 | ***SPOP*** | chr17 | 47688637 | 47688824 | 188 | 188 | 0 |
| WG_IAD68410 | ***SPOP*** | chr17 | 47696338 | 47696475 | 138 | 138 | 0 |
| WG_IAD68410 | ***SPOP*** | chr17 | 47696591 | 47696752 | 162 | 162 | 0 |
| WG_IAD68410 | ***SPOP*** | chr17 | 47699303 | 47699434 | 132 | 132 | 0 |
| WG_IAD68410 | ***SPOP*** | chr17 | 47700090 | 47700177 | 88 | 88 | 0 |
| WG_IAD68410 | ***TP53_10*** | chr17 | 7573927 | 7574033 | 107 | 107 | 0 |
| WG_IAD68410 | ***TP53_9*** | chr17 | 7576853 | 7576926 | 74 | 74 | 0 |
| WG_IAD68410 | ***TP53_8*** | chr17 | 7577019 | 7577155 | 137 | 137 | 0 |
| WG_IAD68410 | ***TP53_7*** | chr17 | 7577499 | 7577608 | 110 | 110 | 0 |
| WG_IAD68410 | ***TP53_6*** | chr17 | 7578177 | 7578289 | 113 | 113 | 0 |
| WG_IAD68410 | ***TP53_5*** | chr17 | 7578371 | 7578554 | 184 | 184 | 0 |
| WG_IAD68410 | ***TP53_4*** | chr17 | 7579312 | 7579590 | 279 | 279 | 0 |
| WG_IAD68410 | ***TP53_2*** | chr17 | 7579839 | 7579940 | 102 | 102 | 0 |
| WG_IAD68410 | ***MEK4_3*** | chr17 | 11958206 | 11958308 | 103 | 103 | 0 |
| WG_IAD68410 | ***MEK4_4*** | chr17 | 11984673 | 11984847 | 175 | 175 | 0 |
| WG_IAD68410 | ***MEK4_5*** | chr17 | 11998892 | 11999011 | 120 | 120 | 0 |
| WG_IAD68410 | ***MEK4_6*** | chr17 | 12011107 | 12011226 | 120 | 120 | 0 |
| WG_IAD68410 | ***MEK4_7*** | chr17 | 12013692 | 12013743 | 52 | 52 | 0 |
| WG_IAD68410 | ***MEK4_8*** | chr17 | 12016550 | 12016677 | 128 | 128 | 0 |
| WG_IAD68410 | ***MEK4_9*** | chr17 | 12028611 | 12028688 | 78 | 78 | 0 |
| WG_IAD68410 | ***MEK4_10*** | chr17 | 12032456 | 12032604 | 149 | 149 | 0 |
| WG_IAD68410 | ***HER2_17*** | chr17 | 37879572 | 37879710 | 139 | 139 | 0 |
| WG_IAD68410 | ***HER2_18*** | chr17 | 37879791 | 37879913 | 107 | 123 | 16 |
| WG_IAD68410 | ***HER2_19*** | chr17 | 37880165 | 37880263 | 99 | 99 | 0 |
| WG_IAD68410 | ***HER2_20*** | chr17 | 37880979 | 37881164 | 186 | 186 | 0 |
| WG_IAD68410 | ***HER2_21*** | chr17 | 37881302 | 37881457 | 156 | 156 | 0 |
| WG_IAD68410 | ***HER2_22*** | chr17 | 37881580 | 37881655 | 76 | 76 | 0 |
| WG_IAD68410 | ***HER2_23*** | chr17 | 37881960 | 37882106 | 147 | 147 | 0 |
| WG_IAD68410 | ***SMAD2*** | chr18 | 45368193 | 45368326 | 134 | 134 | 0 |
| WG_IAD68410 | ***SMAD2*** | chr18 | 45371706 | 45371860 | 155 | 155 | 0 |
| WG_IAD68410 | ***SMAD2*** | chr18 | 45372029 | 45372176 | 148 | 148 | 0 |
| WG_IAD68410 | ***SMAD2*** | chr18 | 45374841 | 45375063 | 223 | 223 | 0 |
| WG_IAD68410 | ***SMAD2*** | chr18 | 45377640 | 45377703 | 64 | 64 | 0 |
| WG_IAD68410 | ***SMAD2*** | chr18 | 45391425 | 45391509 | 85 | 85 | 0 |
| WG_IAD68410 | ***SMAD2*** | chr18 | 45394689 | 45394833 | 145 | 145 | 0 |
| WG_IAD68410 | ***SMAD2*** | chr18 | 45395609 | 45395812 | 204 | 204 | 0 |
| WG_IAD68410 | ***SMAD2*** | chr18 | 45396841 | 45396940 | 100 | 100 | 0 |
| WG_IAD68410 | ***SMAD2*** | chr18 | 45422887 | 45423132 | 246 | 246 | 0 |
| WG_IAD68410 | ***SMAD4*** | chr18 | 48573412 | 48573670 | 259 | 259 | 0 |
| WG_IAD68410 | ***SMAD4*** | chr18 | 48575051 | 48575235 | 185 | 185 | 0 |
| WG_IAD68410 | ***SMAD4*** | chr18 | 48575660 | 48575699 | 40 | 40 | 0 |
| WG_IAD68410 | ***SMAD4*** | chr18 | 48581146 | 48581368 | 199 | 223 | 24 |
| WG_IAD68410 | ***SMAD4*** | chr18 | 48584490 | 48584619 | 130 | 130 | 0 |
| WG_IAD68410 | ***SMAD4*** | chr18 | 48584705 | 48584831 | 127 | 127 | 0 |
| WG_IAD68410 | ***SMAD4*** | chr18 | 48586231 | 48586291 | 61 | 61 | 0 |
| WG_IAD68410 | ***SMAD4*** | chr18 | 48591788 | 48591981 | 194 | 194 | 0 |
| WG_IAD68410 | ***SMAD4*** | chr18 | 48593384 | 48593562 | 179 | 179 | 0 |
| WG_IAD68410 | ***SMAD4*** | chr18 | 48603003 | 48603151 | 149 | 149 | 0 |
| WG_IAD68410 | ***SMAD4*** | chr18 | 48604621 | 48604842 | 222 | 222 | 0 |
| WG_IAD68410 | ***BAX*** | chr19 | 49458181 | 49458224 | 44 | 44 | 0 |
| WG_IAD68410 | ***BAX*** | chr19 | 49458800 | 49458861 | 62 | 62 | 0 |
| WG_IAD68410 | ***BAX*** | chr19 | 49458939 | 49459095 | 157 | 157 | 0 |
| WG_IAD68410 | ***BAX*** | chr19 | 49459450 | 49459595 | 146 | 146 | 0 |
| WG_IAD68410 | ***BAX*** | chr19 | 49464062 | 49464359 | 298 | 298 | 0 |
| WG_IAD68410 | ***BAX*** | chr19 | 49464784 | 49464898 | 115 | 115 | 0 |
| WG_IAD68410 | ***LKB1_1*** | chr19 | 1206880 | 1207202 | 323 | 323 | 0 |
| WG_IAD68410 | ***LKB1_4*** | chr19 | 1220372 | 1220504 | 133 | 133 | 0 |
| WG_IAD68410 | ***LKB1_5*** | chr19 | 1220580 | 1220716 | 137 | 137 | 0 |
| WG_IAD68410 | ***LKB1_6*** | chr19 | 1221212 | 1221339 | 128 | 128 | 0 |
| WG_IAD68410 | ***LKB1_8*** | chr19 | 1222984 | 1223171 | 188 | 188 | 0 |
| WG_IAD68410 | ***GNA11_4*** | chr19 | 3114942 | 3115070 | 129 | 129 | 0 |
| WG_IAD68410 | ***GNA11_5*** | chr19 | 3118922 | 3119051 | 130 | 130 | 0 |
| WG_IAD68410 | ***JAK3_15*** | chr19 | 17945661 | 17945812 | 152 | 152 | 0 |
| WG_IAD68410 | ***JAK3_12*** | chr19 | 17947938 | 17948022 | 85 | 85 | 0 |
| WG_IAD68410 | ***JAK3_3*** | chr19 | 17954189 | 17954300 | 112 | 112 | 0 |
| WG_IAD68410 | ***SRC_14*** | chr20 | 36031574 | 36031813 | 240 | 240 | 0 |
| WG_IAD68410 | ***GNAS_8*** | chr20 | 57484405 | 57484478 | 74 | 74 | 0 |
| WG_IAD68410 | ***GNAS_9*** | chr20 | 57484576 | 57484634 | 59 | 59 | 0 |
| WG_IAD68410 | ***RUNX1*** | chr21 | 36164427 | 36164912 | 486 | 486 | 0 |
| WG_IAD68410 | ***RUNX1*** | chr21 | 36171593 | 36171764 | 172 | 172 | 0 |
| WG_IAD68410 | ***RUNX1*** | chr21 | 36193960 | 36193998 | 39 | 39 | 0 |
| WG_IAD68410 | ***RUNX1*** | chr21 | 36206702 | 36206903 | 202 | 202 | 0 |
| WG_IAD68410 | ***RUNX1*** | chr21 | 36231766 | 36231880 | 115 | 115 | 0 |
| WG_IAD68410 | ***RUNX1*** | chr21 | 36252849 | 36253015 | 167 | 167 | 0 |
| WG_IAD68410 | ***RUNX1*** | chr21 | 36259135 | 36259414 | 280 | 280 | 0 |
| WG_IAD68410 | ***RUNX1*** | chr21 | 36265217 | 36265265 | 49 | 49 | 0 |
| WG_IAD68410 | ***RUNX1*** | chr21 | 36421134 | 36421201 | 68 | 68 | 0 |
| WG_IAD68410 | ***SMARCB1_2*** | chr22 | 24133943 | 24134081 | 139 | 139 | 0 |
| WG_IAD68410 | ***SMARCB1_4*** | chr22 | 24143131 | 24143268 | 138 | 138 | 0 |
| WG_IAD68410 | ***SMARCB1_5*** | chr22 | 24145482 | 24145609 | 128 | 128 | 0 |
| WG_IAD68410 | ***SMARCB1_9*** | chr22 | 24176328 | 24176387 | 60 | 60 | 0 |
| WG_IAD68410 | ***KDM5C*** | chrX | 53221921 | 53222024 | 104 | 104 | 0 |
| WG_IAD68410 | ***KDM5C*** | chrX | 53222144 | 53222519 | 376 | 376 | 0 |
| WG_IAD68410 | ***KDM5C*** | chrX | 53222614 | 53222823 | 210 | 210 | 0 |
| WG_IAD68410 | ***KDM5C*** | chrX | 53222950 | 53223038 | 89 | 89 | 0 |
| WG_IAD68410 | ***KDM5C*** | chrX | 53223316 | 53223925 | 610 | 610 | 0 |
| WG_IAD68410 | ***KDM5C*** | chrX | 53224108 | 53224255 | 148 | 148 | 0 |
| WG_IAD68410 | ***KDM5C*** | chrX | 53224408 | 53224597 | 190 | 190 | 0 |
| WG_IAD68410 | ***KDM5C*** | chrX | 53225093 | 53225241 | 149 | 149 | 0 |
| WG_IAD68410 | ***KDM5C*** | chrX | 53225863 | 53226231 | 369 | 369 | 0 |
| WG_IAD68410 | ***KDM5C*** | chrX | 53226948 | 53227063 | 116 | 116 | 0 |
| WG_IAD68410 | ***KDM5C*** | chrX | 53227667 | 53227824 | 158 | 158 | 0 |
| WG_IAD68410 | ***KDM5C*** | chrX | 53227941 | 53228075 | 135 | 135 | 0 |
| WG_IAD68410 | ***KDM5C*** | chrX | 53228154 | 53228345 | 192 | 192 | 0 |
| WG_IAD68410 | ***KDM5C*** | chrX | 53230727 | 53230931 | 205 | 205 | 0 |
| WG_IAD68410 | ***KDM5C*** | chrX | 53231031 | 53231160 | 130 | 130 | 0 |
| WG_IAD68410 | ***KDM5C*** | chrX | 53239591 | 53239763 | 173 | 173 | 0 |
| WG_IAD68410 | ***KDM5C*** | chrX | 53239853 | 53240044 | 192 | 192 | 0 |
| WG_IAD68410 | ***KDM5C*** | chrX | 53240674 | 53240842 | 169 | 169 | 0 |
| WG_IAD68410 | ***KDM5C*** | chrX | 53240964 | 53241093 | 130 | 130 | 0 |
| WG_IAD68410 | ***KDM5C*** | chrX | 53243866 | 53244034 | 169 | 169 | 0 |
| WG_IAD68410 | ***KDM5C*** | chrX | 53244972 | 53245163 | 192 | 192 | 0 |
| WG_IAD68410 | ***KDM5C*** | chrX | 53245251 | 53245384 | 134 | 134 | 0 |
| WG_IAD68410 | ***KDM5C*** | chrX | 53246320 | 53246464 | 145 | 145 | 0 |
| WG_IAD68410 | ***KDM5C*** | chrX | 53246973 | 53247153 | 181 | 181 | 0 |
| WG_IAD68410 | ***KDM5C*** | chrX | 53247453 | 53247585 | 133 | 133 | 0 |
| WG_IAD68410 | ***KDM5C*** | chrX | 53250016 | 53250103 | 88 | 88 | 0 |
| WG_IAD68410 | ***KDM5C*** | chrX | 53253917 | 53254076 | 160 | 160 | 0 |
| WG_IAD68410 | ***MED12*** | chrX | 70338600 | 70338708 | 109 | 109 | 0 |
| WG_IAD68410 | ***MED12*** | chrX | 70339218 | 70339332 | 115 | 115 | 0 |
| WG_IAD68410 | ***MED12*** | chrX | 70339531 | 70339732 | 202 | 202 | 0 |
| WG_IAD68410 | ***MED12*** | chrX | 70339859 | 70340025 | 167 | 167 | 0 |
| WG_IAD68410 | ***MED12*** | chrX | 70340816 | 70341007 | 192 | 192 | 0 |
| WG_IAD68410 | ***MED12*** | chrX | 70341172 | 70341292 | 121 | 121 | 0 |
| WG_IAD68410 | ***MED12*** | chrX | 70341407 | 70341671 | 265 | 265 | 0 |
| WG_IAD68410 | ***MED12*** | chrX | 70342045 | 70342201 | 157 | 157 | 0 |
| WG_IAD68410 | ***MED12*** | chrX | 70342353 | 70342462 | 110 | 110 | 0 |
| WG_IAD68410 | ***MED12*** | chrX | 70342583 | 70342729 | 147 | 147 | 0 |
| WG_IAD68410 | ***MED12*** | chrX | 70342940 | 70343081 | 142 | 142 | 0 |
| WG_IAD68410 | ***MED12*** | chrX | 70343439 | 70343575 | 137 | 137 | 0 |
| WG_IAD68410 | ***MED12*** | chrX | 70344004 | 70344243 | 240 | 240 | 0 |
| WG_IAD68410 | ***MED12*** | chrX | 70344609 | 70344699 | 91 | 91 | 0 |
| WG_IAD68410 | ***MED12*** | chrX | 70344821 | 70345001 | 181 | 181 | 0 |
| WG_IAD68410 | ***MED12*** | chrX | 70345196 | 70345350 | 155 | 155 | 0 |
| WG_IAD68410 | ***MED12*** | chrX | 70345508 | 70345568 | 61 | 61 | 0 |
| WG_IAD68410 | ***MED12*** | chrX | 70345881 | 70346009 | 129 | 129 | 0 |
| WG_IAD68410 | ***MED12*** | chrX | 70346186 | 70346339 | 154 | 154 | 0 |
| WG_IAD68410 | ***MED12*** | chrX | 70346814 | 70346987 | 174 | 174 | 0 |
| WG_IAD68410 | ***MED12*** | chrX | 70347181 | 70347322 | 142 | 142 | 0 |
| WG_IAD68410 | ***MED12*** | chrX | 70347738 | 70347975 | 238 | 238 | 0 |
| WG_IAD68410 | ***MED12*** | chrX | 70348141 | 70348295 | 155 | 155 | 0 |
| WG_IAD68410 | ***MED12*** | chrX | 70348443 | 70348573 | 131 | 131 | 0 |
| WG_IAD68410 | ***MED12*** | chrX | 70348959 | 70349070 | 112 | 112 | 0 |
| WG_IAD68410 | ***MED12*** | chrX | 70349161 | 70349284 | 124 | 124 | 0 |
| WG_IAD68410 | ***MED12*** | chrX | 70349525 | 70349710 | 186 | 186 | 0 |
| WG_IAD68410 | ***MED12*** | chrX | 70349880 | 70350069 | 190 | 190 | 0 |
| WG_IAD68410 | ***MED12*** | chrX | 70351395 | 70351476 | 82 | 82 | 0 |
| WG_IAD68410 | ***MED12*** | chrX | 70351918 | 70352061 | 144 | 144 | 0 |
| WG_IAD68410 | ***MED12*** | chrX | 70352222 | 70352393 | 172 | 172 | 0 |
| WG_IAD68410 | ***MED12*** | chrX | 70352690 | 70352811 | 122 | 122 | 0 |
| WG_IAD68410 | ***MED12*** | chrX | 70352968 | 70353067 | 100 | 100 | 0 |
| WG_IAD68410 | ***MED12*** | chrX | 70354202 | 70354321 | 120 | 120 | 0 |
| WG_IAD68410 | ***MED12*** | chrX | 70354558 | 70354703 | 146 | 146 | 0 |
| WG_IAD68410 | ***MED12*** | chrX | 70354937 | 70355108 | 172 | 172 | 0 |
| WG_IAD68410 | ***MED12*** | chrX | 70356126 | 70356510 | 385 | 385 | 0 |
| WG_IAD68410 | ***MED12*** | chrX | 70356724 | 70356884 | 161 | 161 | 0 |
| WG_IAD68410 | ***MED12*** | chrX | 70357032 | 70357238 | 207 | 207 | 0 |
| WG_IAD68410 | ***MED12*** | chrX | 70357403 | 70357490 | 88 | 88 | 0 |
| WG_IAD68410 | ***MED12*** | chrX | 70357571 | 70357798 | 228 | 228 | 0 |
| WG_IAD68410 | ***MED12*** | chrX | 70360480 | 70360712 | 233 | 233 | 0 |
| WG_IAD68410 | ***MED12*** | chrX | 70361075 | 70361225 | 151 | 151 | 0 |
| WG_IAD68410 | ***MED12*** | chrX | 70361728 | 70361819 | 92 | 92 | 0 |
| WG_IAD68410 | ***MED12*** | chrX | 70362020 | 70362073 | 54 | 54 | 0 |
| WG_IAD68410 | ***ATRX*** | chrX | 76763824 | 76764112 | 289 | 289 | 0 |
| WG_IAD68410 | ***ATRX*** | chrX | 76776261 | 76776399 | 139 | 139 | 0 |
| WG_IAD68410 | ***ATRX*** | chrX | 76776876 | 76776981 | 106 | 106 | 0 |
| WG_IAD68410 | ***ATRX*** | chrX | 76777736 | 76777871 | 136 | 136 | 0 |
| WG_IAD68410 | ***ATRX*** | chrX | 76778725 | 76778884 | 160 | 160 | 0 |
| WG_IAD68410 | ***ATRX*** | chrX | 76812917 | 76813121 | 205 | 205 | 0 |
| WG_IAD68410 | ***ATRX*** | chrX | 76814135 | 76814322 | 188 | 188 | 0 |
| WG_IAD68410 | ***ATRX*** | chrX | 76829710 | 76829828 | 119 | 119 | 0 |
| WG_IAD68410 | ***ATRX*** | chrX | 76845299 | 76845415 | 117 | 117 | 0 |
| WG_IAD68410 | ***ATRX*** | chrX | 76849161 | 76849324 | 164 | 164 | 0 |
| WG_IAD68410 | ***ATRX*** | chrX | 76854875 | 76855054 | 180 | 180 | 0 |
| WG_IAD68410 | ***ATRX*** | chrX | 76855196 | 76855294 | 99 | 99 | 0 |
| WG_IAD68410 | ***ATRX*** | chrX | 76855898 | 76856038 | 141 | 141 | 0 |
| WG_IAD68410 | ***ATRX*** | chrX | 76872076 | 76872203 | 128 | 128 | 0 |
| WG_IAD68410 | ***ATRX*** | chrX | 76874269 | 76874454 | 186 | 186 | 0 |
| WG_IAD68410 | ***ATRX*** | chrX | 76875858 | 76876005 | 148 | 148 | 0 |
| WG_IAD68410 | ***ATRX*** | chrX | 76888690 | 76888877 | 188 | 188 | 0 |
| WG_IAD68410 | ***ATRX*** | chrX | 76889049 | 76889205 | 157 | 157 | 0 |
| WG_IAD68410 | ***ATRX*** | chrX | 76890080 | 76890199 | 120 | 120 | 0 |
| WG_IAD68410 | ***ATRX*** | chrX | 76891401 | 76891552 | 152 | 152 | 0 |
| WG_IAD68410 | ***ATRX*** | chrX | 76907599 | 76907848 | 250 | 250 | 0 |
| WG_IAD68410 | ***ATRX*** | chrX | 76909583 | 76909695 | 113 | 113 | 0 |
| WG_IAD68410 | ***ATRX*** | chrX | 76912045 | 76912148 | 104 | 104 | 0 |
| WG_IAD68410 | ***ATRX*** | chrX | 76918866 | 76919052 | 187 | 187 | 0 |
| WG_IAD68410 | ***ATRX*** | chrX | 76920129 | 76920272 | 144 | 144 | 0 |
| WG_IAD68410 | ***ATRX*** | chrX | 76931716 | 76931798 | 83 | 83 | 0 |
| WG_IAD68410 | ***ATRX*** | chrX | 76937007 | 76940090 | 3084 | 3084 | 0 |
| WG_IAD68410 | ***ATRX*** | chrX | 76940426 | 76940503 | 78 | 78 | 0 |
| WG_IAD68410 | ***ATRX*** | chrX | 76944306 | 76944425 | 120 | 120 | 0 |
| WG_IAD68410 | ***ATRX*** | chrX | 76949308 | 76949431 | 124 | 124 | 0 |
| WG_IAD68410 | ***ATRX*** | chrX | 76952060 | 76952197 | 138 | 138 | 0 |
| WG_IAD68410 | ***ATRX*** | chrX | 76953066 | 76953128 | 63 | 63 | 0 |
| WG_IAD68410 | ***ATRX*** | chrX | 76954057 | 76954122 | 66 | 66 | 0 |
| WG_IAD68410 | ***ATRX*** | chrX | 76972603 | 76972725 | 123 | 123 | 0 |
| WG_IAD68410 | ***ATRX*** | chrX | 77041463 | 77041492 | 30 | 30 | 0 |

**REFERENCES**

1. Klimstra DS, Kloppel G, La Rosa S, Rindi G. Classification of neuroendocrine neoplasms of the digestive system. In: WHO Classification of Tumours of the Digestive System. *5th ed Lyon: International Agency for Research on Cancer.* 2019.

2. WHO Classification of Tumours Editorial Board. Thoracic Tumours. *5th ed Lyon (France): International Agency for Research on Cancer.* 2021.

3. Eisenhauer EA, Therasse P, Bogaerts J, et al. New response evaluation criteria in solid tumours: revised RECIST guideline (version 1.1). *Eur J Cancer.* 2009;45(2):228-247.

4. World Medical A. World Medical Association Declaration of Helsinki: ethical principles for medical research involving human subjects. *JAMA.* 2013;310(20):2191-2194.

5. Vernieri C, Nichetti F, Lalli L, et al. Impact of Baseline and On-Treatment Glycemia on Everolimus-Exemestane Efficacy in Patients with Hormone Receptor-Positive Advanced Breast Cancer (EVERMET). *Clin Cancer Res.* 2021;27(12):3443-3455.

6. Matthews DR, Hosker JP, Rudenski AS, Naylor BA, Treacher DF, Turner RC. Homeostasis model assessment: insulin resistance and beta-cell function from fasting plasma glucose and insulin concentrations in man. *Diabetologia.* 1985;28(7):412-419.
