# Supplementary material for: Safety and antitumor activity of metformin plus lanreotide in patients with advanced gastro-intestinal or lung neuroendocrine tumors: the phase Ib trial MetNET2
Source: J Hematol Oncol. 2023 Dec 14;16:119. doi: 10.1186/s13045-023-01510-9 (PMC10722662; doi:10.1186/s13045-023-01510-9)
Supplement: Supplementary file 2 — Additional file 2. Table S1: Demographic, clinic-pathological and metabolic characteristics of enrolled patients. [file 13045_2023_1510_MOESM2_ESM.docx]

**ADDITIONAL FILE 2**

**Table S1.** Demographic, clinic-pathological and metabolic characteristics of enrolled patients

|  | Non-diabetic | Diabetic | Overall | *P-* Value * |
| --- | --- | --- | --- | --- |
|  | (N=14) | (N=6) | (N=20) |  |
| **Gender** |  |  |  | 0.6424 |
| Female | 5 (35.7%) | 3 (50.0%) | 8 (40.0%) |  |
| Male | 9 (64.3%) | 3 (50.0%) | 12 (60.0%) |  |
| **Age** |  |  |  | 0.2704 |
| Mean (SD) | 64.4 (12.6) | 65.7 (5.05) | 64.8 (10.8) |  |
| Median [IQR] | 66.5 [52.0, 74.8] | 67.0 [63.8, 68.8] | 67.0 [55.8, 73.3] |  |
| **ECOG Performance statu**s |  |  |  | 0.5211 |
| 0 | 13 (92.9%) | 5 (83.3%) | 18 (90.0%) |  |
| 1 | 1 (7.1%) | 1 (16.7%) | 2 (10.0%) |  |
| **Primary tumor** |  |  |  | 1.0000 |
| Lung | 1 (7.1%) | 0 (0%) | 1 (5.0%) |  |
| Midgut | 10 (71.4%) | 6 (100%) | 16 (80.0%) |  |
| Rectal | 1 (7.1%) | 0 (0%) | 1 (5.0%) |  |
| Thyme | 1 (7.1%) | 0 (0%) | 1 (5.0%) |  |
| Vater papilla | 1 (7.1%) | 0 (0%) | 1 (5.0%) |  |
| **Tumor grading** |  |  |  | 0.6027 |
| G1 | 6 (42.9%) | 1 (16.7%) | 7 (35.0%) |  |
| G2 | 8 (57.1%) | 4 (66.7%) | 12 (60.0%) |  |
| Missing | 0 (0%) | 1 (16.7%) | 1 (5.0%) |  |
| **Number of metastatic sites** |  |  |  | 1.0000 |
| 1 | 4 (28.6%) | 1 (16.7%) | 5 (25.0%) |  |
| >1 | 10 (71.4%) | 5 (83.3%) | 15 (75.0%) |  |
| **Metastatic sites** |  |  |  | 0.5211 |
| Extra-liver | 11 (78.6%) | 6 (100.0%) | 17 (85.0%) |  |
| Liver only | 3 (21.4%) | 0 (0.0%) | 3 (15.0%) |  |
| **Liver burden ≥25%** |  |  |  | 0.5378 |
| Yes | 2 (14.3%) | 2 (33.3%) | 4 (20.0%) |  |
| No | 10 (71.4%) | 3 (50.0%) | 13 (65.0%) |  |
| Missing* (no liver disease) | 2 (14.3%) | 1 (16.7%) | 3 (15.0%) |  |
| **Primary tumor resection** |  |  |  | 1.0000 |
| No | 2 (14.3%) | 1 (16.7%) | 3 (15.0%) |  |
| Yes | 12 (85.7%) | 5 (83.3%) | 17 (85.0%) |  |
| **Liver-directed treatments** |  |  |  | 0.2604 |
| Liver resection | 5 (35.7%) | 0 (0%) | 5 (25.0%) |  |
| None | 9 (64.3%) | 6 (100%) | 15 (75.0%) |  |
| **Prior line of therapy** |  |  |  | 0.4389 |
| 0 | 4 (28.6%) | 1 (16.7%) | 5 (25.0%) |  |
| 1  *SSA*  *Chemotherapy* | 8 (57.1%) | 2 (33.3%) | 10 (50.0%)  9 (45.0%)  1 (5.0%) |  |
| 2  *PRRT plus SSA*  *SSA*  *Chemotherapy* | 2 (14.3%) | 3 (50.0%) | 5 (25.0%)  3 (15.0%)  1 (5.0%)  1 (5.0%) |  |
| **Functioning patients** |  |  |  | 1.0000 |
| Functioning | 3 (21.4%) | 1 (16.7%) | 4 (20.0%) |  |
| No functioning | 11 (78.5%) | 5 (83.3%) | 16 (80.0%) |  |
| **Baseline glucose (mg/dL)** |  |  |  | 0.2414 |
| Median [IQR] | 112 [91.0, 117] | 118 [115, 123] | 116 [107, 119] |  |
| Missing | 3 (21.4%) | 1 (16.7%) | 4 (20.0%) |  |
| **Baseline insulin (mU/mL)** |  |  |  | 0.5596 |
| Median [IQR] | 5.70 [4.10, 7.35] | 3.65 [2.53, 5.12] | 4.70 [3.80, 6.64] |  |
| Missing | 3 (21.4%) | 2 (33.3%) | 5 (25.0%) |  |
| **HbA1c** |  |  |  | 0.5687 |
| Median [IQR] | 38.0 [36.8, 40.5] | 41.0 [40.0, 44.0] | 40.0 [37.0, 42.0] |  |
| Missing | 2 (14.3%) | 1 (16.7%) | 3 (15.0%) |  |
| **OGTT results at baseline** |  |  |  |  |
| Normoglycaemic status | 4 (20%) | 0 (0%) |  |  |
| IFG | 8 (40%) | 0 (0%) |  |  |
| IGT | 2 (10%) | 0 (0%) |  |  |
| T2-diabetes | 0 (0%) | 6 (30%) |  |  |
| **Baseline HOMA-IR index** |  |  |  | 0.4497 |
| Median [IQR] | 24.2 [21.0, 30.4] | 19.0 [13.1, 28.0] | 24.2 [19.6, 30.4] |  |
| Missing | 3 (21.4%) | 2 (33.3%) | 5 (25.0%) |  |
| **Baseline Total Cholesterolemia** |  |  |  | 0.4530 |
| Median [IQR] | 185.0[175.5, 201.5] | 160.5[153.2, 167.8] | 176.0 [164.0, 199.0] |  |
| Missing | 3 (21.4%) | 0 (0.0%) | 3 (15.0%) |  |
| **Baseline Triglyceridemia** |  |  |  | 0.4530 |
| Median [IQR] | 103.0 [79.5, 151.0] | 119.5 [83.5, 178.0] | 115.0 [77.0, 159.0] |  |
| Missing | 3 (21.4%) | 0 (0.0%) | 3 (15.0%) |  |
| **BMI** |  |  |  | 0.4497 |
| Median [Q1, Q3] | 24.6 [20.9, 26.6] | 21.4 [19.5, 26.6] | 24.1 [19.7, 26.8] |  |
| Missing | 4 (28.6%) | 1 (16.7%) | 5 (25.0%) |  |
