# Supplementary material for: Safety and antitumor activity of metformin plus lanreotide in patients with advanced gastro-intestinal or lung neuroendocrine tumors: the phase Ib trial MetNET2
Source: J Hematol Oncol. 2023 Dec 14;16:119. doi: 10.1186/s13045-023-01510-9 (PMC10722662; doi:10.1186/s13045-023-01510-9)
Supplement: Supplementary file 3 — Additional file 3. Fig. S1: Spaghetti plots of dose intensity for metformin administration (A) and for dose intensity of Lanreotide ATG (B). [file 13045_2023_1510_MOESM3_ESM.docx]

**ADDITIONAL FILE 3**

**Figure S1.** Spaghetti plots of dose intensity for metformin administration (**A**) and for dose intensity of Lanreotide ATG (**B**). Lanreotide ATG and metformin were administered for a median of 18.9 months (IQR range 13.5-22.8 months) and 15.9 months (IQR range 5.8-21 months), respectively.

**A B**

**
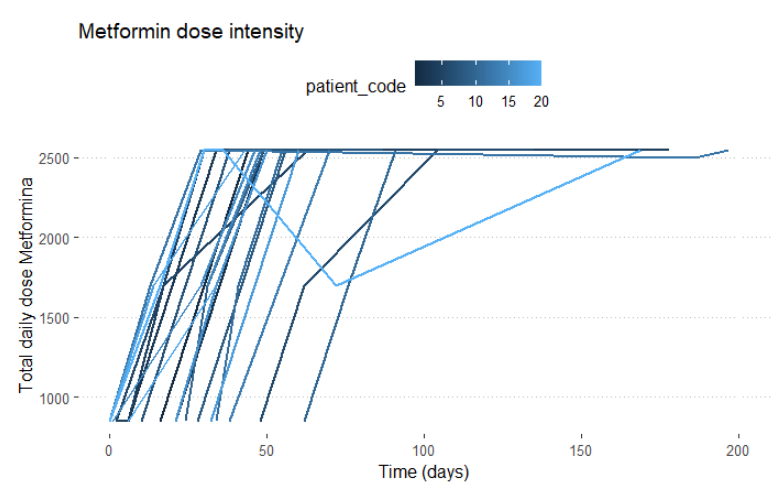

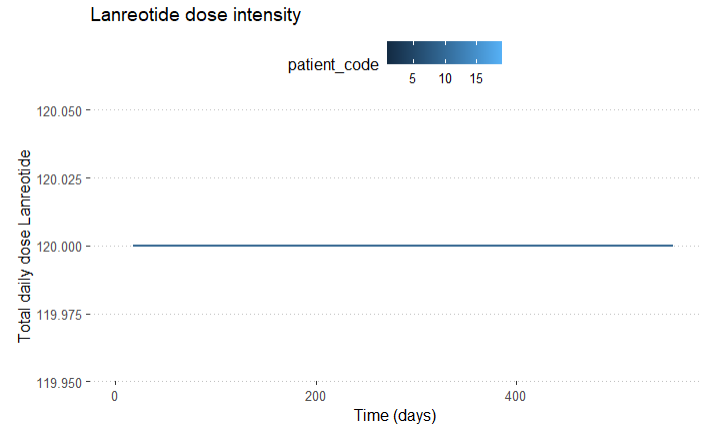
**
