# Supplementary material for: Safety and antitumor activity of metformin plus lanreotide in patients with advanced gastro-intestinal or lung neuroendocrine tumors: the phase Ib trial MetNET2
Source: J Hematol Oncol. 2023 Dec 14;16:119. doi: 10.1186/s13045-023-01510-9 (PMC10722662; doi:10.1186/s13045-023-01510-9)
Supplement: Supplementary file 4 — Additional file 4. Table S2: Metformin and Lanreotide ATG drug exposure and relative dose intensity according to diabetic status. [file 13045_2023_1510_MOESM4_ESM.docx]

**ADDITIONAL FILE 4**

**Table S2. Metformin and Lanreotide ATG drug exposure and relative dose intensity according to diabetic status.** The median number of Lanreotide ATG cycles administered during the study was 20.5 (IQR range, 14.7-24.7 administrations). Average metformin relative dose intensity (RDI), as measured as the percentage of the maximum planned metformin dose (2550 mg/day), was 86.4% (median 94.9%; IQR 82.6% - 98.0%). Overall, 19 (95%) patients reached the maximum metformin dose of 2550 mg/day, while only one patient (5%) reached 1700 mg as the maximum metformin daily dose. No differences were found, in terms of treatment exposure, RDI, metformin dose reductions, or median number of lanreotide ATG cycles, between diabetic and non-diabetic patients.

|  |  | Overall | **Non-Diabetics** | **Diabetics** | *P-* Value * |
| --- | --- | --- | --- | --- | --- |
| **Metformin** | Treatment exposure | Average: 0.8711  Median [IQR]: 0.9082 [0.8495, 0.9447] | Average: 0.897  Median [IQR]: 0.9120 [0.8853, 0.9382] | Average: 0.8323  Median [IQR]: 0.8458 [0.7544, 0.9469] | 0.4478 |
|  | RDI | Average: 86.4%  Median [IQR]: 94.9% [82.6%, 98.0%] | Average: 86.35%  Median [IQR]: 94.94% [83.41%, 97.49%] | Average: 86.35%  Median [IQR]: 90.61% [77.49%, 97.95%] | 0.4568 |
|  | Nr. of patients with reduction | 7 (35%) | 5 (35.71%) | 2 (33.33%) | 1.0000 |
| **Lanreotide** | Treatment exposure | Average: 0.7112  Median [IQR]: 0.8766 [0.1521, 0.9487] | Average: 0.7328  Median [IQR]: 0.9327 [0.0-0.9499] | Average: 0.6723  Median [IQR]: 0.8409 [0.6084-0.9122] | 0.4434 |
|  | Median number of administrations | 20.5 (IQR range, 14.7-24.7) | 23.0 [17.9-24.7] | 16.3 [11.4-21.0] | 0.5373 |
|  | Nr. of patients with reduction | 0 | 0 | 0 | 1.0000 |

* The associations were tested using Wilcoxon-Mann-Whitney test for continuous data and Fisher’s exact test for categorical data. Unknown and missing values were excluded from the statistical tests. RDI: Relative dose intensity.
