# Supplementary material for: Safety and antitumor activity of metformin plus lanreotide in patients with advanced gastro-intestinal or lung neuroendocrine tumors: the phase Ib trial MetNET2
Source: J Hematol Oncol. 2023 Dec 14;16:119. doi: 10.1186/s13045-023-01510-9 (PMC10722662; doi:10.1186/s13045-023-01510-9)
Supplement: Supplementary file 5 — Additional file 5. Table S3: Differences in the incidence of trAEs between non-diabetic patients (N=14) and diabetic patients (N=6). [file 13045_2023_1510_MOESM5_ESM.docx]

**ADDITIONAL FILE 5**

**Table S3.** **Differences in the incidence of trAEs between non-diabetic patients (N=14) and diabetic patients (N=6).**

|  | **Non-diabetics (N=14)** | **Diabetics**  **(N=6)** | ***P*** | **Non-diabetics** | **Diabetics** | ***P*** | **Non-diabetics** | **Diabetics** | ***P*** | **Non-diabetics** | **Diabetics** | ***P*** |
| --- | --- | --- | --- | --- | --- | --- | --- | --- | --- | --- | --- | --- |
| **Adverse event^a^** | **Any grade** | **Any grade** |  | **G1** | **G1** |  | **G2** | **G2** |  | **G3** | **G3** |  |
| Abdominal pain | 2 (14.3%) | 3 (50.0%) | 0.13 | 1 (7.1%) | 1 (16.7%) | 0.52 | 0 (0.0%) | 2 (33.3%) | 0.07 | 1 (7.1%) | 0 (0.0%) | 1.00 |
| Acute renal failure | 0 (0.0%) | 1 (16.7%) | 0.30 | 0 (0.0%) | 0 (0.0%) | 1.00 | 0 (0.0%) | 0 (0.0%) | 1.00 | 0 (0.0%) | 1 (16.7%) | 1.00 |
| Anorexia | 3 (21.4%) | 2 (33.3%) | 1.00 | 2 (14.3%) | 2 (33.3%) | 0.60 | 1(7.1%) | 0 (0.0%) | 1.00 | 0 (0.0%) | 0 (0.0%) | 1.00 |
| Asthenia | 6 (42.9%) | 2 (33.3%) | 1.00 | 2 (14.3%) | 2 (33.3%) | 0.60 | 4 (28.6%) | 0 (0.0%) | 0.26 | 0 (0.0%) | 0 (0.0%) | 1.00 |
| Diarrhea | 9 (64.3%) | 6 (100.0%) | 0.26 | 3 (21.4%) | 2 (33.3%) | 1.00 | 5 (35.7%) | 4 (66.7%) | 0.33 | 1  (7.1%) | 0 (0.0%) | 1.00 |
| Emesis | 3 (21.4%) | 1 (16.7%) | 1.00 | 2 (14.3%) | 1 (16.7%) | 1.00 | 1(7.1%) | 0 (0.0%) | 1.00 | 0 (0.0%) | 0 (0.0%) | 1.00 |
| Hypercholesterolemia | 6 (42.9%) | 2 (33.3%) | 1.00 | 6 (42.9%) | 2 (33.3%) | 1.00 | 0 (0.0%) | 0 (0.0%) | 1.00 | 0 (0.0%) | 0 (0.0%) | 1.00 |
| Creatinine increase | 4 (28.6%) | 0 (0.0%) | 0.26 | 4 (28.6%) | 0 (0.0%) | 0.26 | 0 (0.0%) | 0 (0.0%) | 1.00 | 0 (0.0%) | 0 (0.0%) | 1.00 |
| Hyperglycemia | 8 (57.1%) | 3  (50.0%) | 1.00 | 8 (57.1%) | 3(50.0%) | 1.00 | 0 (0.0%) | 0 (0.0%) | 1.00 | 0 (0.0%) | 0 (0.0%) | 1.00 |
| Hyperkaliemia | 1(7.1%) | 0 (0.0%) | 1.00 | 1(7.1%) | 0 (0.0%) | 1.00 | 0 (0.0%) | 0 (0.0%) | 1.00 | 0 (0.0%) | 0 (0.0%) | 1.00 |
| Hypertriglyceridemia | 2 (14.3%) | 2 (33.3%) | 0.54 | 2 (14.3%) | 1 (16.7%) | 1.00 | 0 (0.0%) | 1 (16.7%) | 1.00 | 0 (0.0%) | 0 (0.0%) | 1.00 |
| Hyperuricemia | 2 (14.3%) | 1 (16.7%) | 1.00 | 2 (14.3%) | 1 (16.7%) | 1.00 | 0 (0.0%) | 0 (0.0%) | 1.00 | 0 (0.0%) | 0 (0.0%) | 1.00 |
| Hypokalemia | 1(7.1%) | 0 (0.0%) | 1.00 | 0 (0.0%) | 0 (0.0%) | 1.00 | 1(7.1%) | 0 (0.0%) | 1.00 | 0 (0.0%) | 0 (0.0%) | 1.00 |
| Hypomagnesemia | 6 (42.9%) | 1 (16.7%) | 0.35 | 5 (35.7%) | 1 (16.7%) | 0.61 | 1(7.1%) | 0 (0.0%) | 1.00 | 0 (0.0%) | 0 (0.0%) | 1.00 |
| Hypophosphatemia | 1(7.1%) | 0 (0.0%) | 1.00 | 1(7.1%) | 0 (0.0%) | 1.00 | 0 (0.0%) | 0 (0.0%) | 1.00 | 0 (0.0%) | 0 (0.0%) | 1.00 |
| Intestinal bloating | 1(7.1%) | 1 (16.7%) | 0.52 | 1(7.1%) | 1 (16.7%) | 0.52 | 0 (0.0%) | 0 (0.0%) | 1.00 | 0 (0.0%) | 0 (0.0%) | 1.00 |
| Hypocalcemia | 1(7.1%) | 0 (0.0%) | 1.00 | 1(7.1%) | 0 (0.0%) | 1.00 | 0 (0.0%) | 0 (0.0%) | 1.00 | 0 (0.0%) | 0 (0.0%) | 1.00 |
| Nausea | 3 (21.4%) | 1 (16.7%) | 1.00 | 2 (14.3%) | 1 (16.7%) | 1.00 | 1(7.1%) | 0 (0.0%) | 1.00 | 0 (0.0%) | 0 (0.0%) | 1.00 |
| Steatorrhea | 1(7.1%) | 1 (16.7%) | 0.52 | 0 (0.0%) | 0 (0.0%) | 1.00 | 1(7.1%) | 1 (16.7%) | 0.52 | 0 (0.0%) | 0 (0.0%) | 1.00 |

NOTE: ^a^ Patients with ≥ 2 TE-AEs reported under the same preferred term were only counted once using the highest Common Terminology Criteria for Adverse Events grade.
