# Supplementary material for: Safety and antitumor activity of metformin plus lanreotide in patients with advanced gastro-intestinal or lung neuroendocrine tumors: the phase Ib trial MetNET2
Source: J Hematol Oncol. 2023 Dec 14;16:119. doi: 10.1186/s13045-023-01510-9 (PMC10722662; doi:10.1186/s13045-023-01510-9)
Supplement: Supplementary file 6 — Additional file 6. Table S4: Treatment-Emergent Adverse Events (TE-AEs). [file 13045_2023_1510_MOESM6_ESM.docx]

**ADDITIONAL FILE 6**

**Table S4.** **Treatment-Emergent Adverse Events (TE-AEs).**

| **Preferred AE Term^a^** | **Any grade** | **Grade 1** | **Grade 2** | **Grade 3** | **Grade 4** |
| --- | --- | --- | --- | --- | --- |
| Diarrhea | 15 (75.0%) | 5 (25.0%) | 9 (45.0%) | 1 (5.0%) | 0 (0.0%) |
| Hyperglycemia | 11 (55.0%) | 11 (55.0%) | 0 (0.0%) | 0 (0.0%) | 0 (0.0%) |
| Asthenia | 8 (40.0%) | 4 (20.0%) | 4 (20.0%) | 0 (0.0%) | 0 (0.0%) |
| Hypercholesterolemia | 8 (40.0%) | 8 (40.0%) | 0 (0.0%) | 0 (0.0%) | 0 (0.0%) |
| Hypomagnesemia | 7 (35.0%) | 6 (30.0%) | 1 (5.0%) | 0 (0.0%) | 0 (0.0%) |
| Abdominal pain | 5 (25.0%) | 2 (10.0%) | 2 (10.0%) | 1 (5.0%) | 0 (0.0%) |
| Anorexia | 5 (25.0%) | 4 (20.0%) | 1 (5.0%) | 0 (0.0%) | 0 (0.0%) |
| Creatinine increase | 4 (20.0%) | 4 (20.0%) | 0 (0.0%) | 0 (0.0%) | 0 (0.0%) |
| Arthralgias | 4 (20.0%) | 2 (10.0%) | 1 (5.0%) | 1 (5.0%) | 0 (0.0%) |
| Emesis | 4 (20.0%) | 3 (15.0%) | 1 (5.0%) | 0 (0.0%) | 0 (0.0%) |
| Hypertriglyceridemia | 4 (20.0%) | 3 (15.0%) | 1 (5.0%) | 0 (0.0%) | 0 (0.0%) |
| Nausea | 4 (20.0%) | 3 (15.0%) | 1 (5.0%) | 0 (0.0%) | 0 (0.0%) |
| Hyperuricemia | 3 (15.0%) | 3 (15.0%) | 0 (0.0%) | 0 (0.0%) | 0 (0.0%) |
| ALT/AST increase | 3 (15.0%) | 3 (15.0%) | 0 (0.0%) | 0 (0.0%) | 0 (0.0%) |
| Flushing | 2 (10.0%) | 1 (5.0%) | 1 (5.0%) | 0 (0.0%) | 0 (0.0%) |
| GGT elevation | 2 (10.0%) | 1 (5.0%) | 1 (5.0%) | 0 (0.0%) | 0 (0.0%) |
| Edema | 2 (10.0%) | 2 (10.0%) | 0 (0.0%) | 0 (0.0%) | 0 (0.0%) |
| Confusion | 2 (10.0%) | 2 (10.0%) | 0 (0.0%) | 0 (0.0%) | 0 (0.0%) |
| Hyponatremia | 2 (10.0%) | 2 (10.0%) | 0 (0.0%) | 0 (0.0%) | 0 (0.0%) |
| Insomnia | 2 (10.0%) | 2 (10.0%) | 0 (0.0%) | 0 (0.0%) | 0 (0.0%) |
| Intestinal bloating | 2 (10.0%) | 2 (10.0%) | 0 (0.0%) | 0 (0.0%) | 0 (0.0%) |
| Pruritus | 2 (10.0%) | 2 (10.0%) | 0 (0.0%) | 0 (0.0%) | 0 (0.0%) |
| Steatorrhea | 2 (10.0%) | 0 (0.0%) | 2 (10.0%) | 0 (0.0%) | 0 (0.0%) |
| Acute renal failure | 1 (5.0%) | 0 (0.0%) | 0 (0.0%) | 1 (5.0%) | 0 (0.0%) |
| ALP elevation | 1 (5.0%) | 1 (5.0%) | 0 (0.0%) | 0 (0.0%) | 0 (0.0%) |
| Anemia | 1 (5.0%) | 0 (0.0%) | 1 (5.0%) | 0 (0.0%) | 0 (0.0%) |
| Arterial hypertension | 1 (5.0%) | 1 (5.0%) | 0 (0.0%) | 0 (0.0%) | 0 (0.0%) |
| Cephalgia | 1 (5.0%) | 1 (5.0%) | 0 (0.0%) | 0 (0.0%) | 0 (0.0%) |
| Cerebral hemorrhage | 1 (5.0%) | 0 (0.0%) | 0 (0.0%) | 0 (0.0%) | 1 (5.0%) |
| Constipation | 1 (5.0%) | 1 (5.0%) | 0 (0.0%) | 0 (0.0%) | 0 (0.0%) |
| Cough | 1 (5.0%) | 1 (5.0%) | 0 (0.0%) | 0 (0.0%) | 0 (0.0%) |
| Dental abscess | 1 (5.0%) | 1 (5.0%) | 0 (0.0%) | 0 (0.0%) | 0 (0.0%) |
| Dysgeusia | 1 (5.0%) | 1 (5.0%) | 0 (0.0%) | 0 (0.0%) | 0 (0.0%) |
| Diverticulitis | 1 (5.0%) | 1 (5.0%) | 0 (0.0%) | 0 (0.0%) | 0 (0.0%) |
| Dysuria | 1 (5.0%) | 0 (0.0%) | 0 (0.0%) | 1 (5.0%) | 0 (0.0%) |
| Extrasystole | 1 (5.0%) | 0 (0.0%) | 0 (0.0%) | 1 (5.0%) | 0 (0.0%) |
| Fever | 1 (5.0%) | 1 (5.0%) | 0 (0.0%) | 0 (0.0%) | 0 (0.0%) |
| Hematuria | 1 (5.0%) | 1 (5.0%) | 0 (0.0%) | 0 (0.0%) | 0 (0.0%) |
| Herpes labialis | 1 (5.0%) | 1 (5.0%) | 0 (0.0%) | 0 (0.0%) | 0 (0.0%) |
| Hyperbilirubinemia | 1 (5.0%) | 1 (5.0%) | 0 (0.0%) | 0 (0.0%) | 0 (0.0%) |
| Hypercalcemia | 1 (5.0%) | 1 (5.0%) | 0 (0.0%) | 0 (0.0%) | 0 (0.0%) |
| Hyperkalemia | 1 (5.0%) | 1 (5.0%) | 0 (0.0%) | 0 (0.0%) | 0 (0.0%) |
| Hyperphosphatemia | 1 (5.0%) | 0 (0.0%) | 1 (5.0%) | 0 (0.0%) | 0 (0.0%) |
| Hypokalemia | 1 (5.0%) | 0 (0.0%) | 1 (5.0%) | 0 (0.0%) | 0 (0.0%) |
| Hypophosphatemia | 1 (5.0%) | 1 (5.0%) | 0 (0.0%) | 0 (0.0%) | 0 (0.0%) |
| Decreased appetite | 1 (5.0%) | 1 (5.0%) | 0 (0.0%) | 0 (0.0%) | 0 (0.0%) |
| Hypocalcemia | 1 (5.0%) | 1 (5.0%) | 0 (0.0%) | 0 (0.0%) | 0 (0.0%) |
| Muscles cramps | 1 (5.0%) | 1 (5.0%) | 0 (0.0%) | 0 (0.0%) | 0 (0.0%) |
| Nasal bleeding | 1 (5.0%) | 1 (5.0%) | 0 (0.0%) | 0 (0.0%) | 0 (0.0%) |
| Nocturia | 1 (5.0%) | 1 (5.0%) | 0 (0.0%) | 0 (0.0%) | 0 (0.0%) |
| Palpitations | 1 (5.0%) | 1 (5.0%) | 0 (0.0%) | 0 (0.0%) | 0 (0.0%) |
| Platelet count increase | 1 (5.0%) | 1 (5.0%) | 0 (0.0%) | 0 (0.0%) | 0 (0.0%) |
| Pneumonia | 1 (5.0%) | 0 (0.0%) | 0 (0.0%) | 1 (5.0%) | 0 (0.0%) |
| Renal colic | 1 (5.0%) | 0 (0.0%) | 1 (5.0%) | 0 (0.0%) | 0 (0.0%) |
| Thoracic folliculitis | 1 (5.0%) | 1 (5.0%) | 0 (0.0%) | 0 (0.0%) | 0 (0.0%) |
| Urinary tract infection | 1 (5.0%) | 0 (0.0%) | 1 (5.0%) | 0 (0.0%) | 0 (0.0%) |
| Vertigo | 1 (5.0%) | 1 (5.0%) | 0 (0.0%) | 0 (0.0%) | 0 (0.0%) |
| Xerostomia | 1 (5.0%) | 1 (5.0%) | 0 (0.0%) | 0 (0.0%) | 0 (0.0%) |

NOTE: Any-grade treatment-related adverse events (TR-AEs) occurred in 100% of patients.

^a^ Patients with ≥ 2 treatment-emergent AEs (TE-AEs) reported with the same preferred term were only counted once using the highest Common Terminology Criteria for Adverse Events grade.

^b^ Patients may have experienced multiple AEs per Common Terminology Criteria for Adverse Events grade. Grade 3 TE-AEs occurred in 5 (25%) patients and included extrasystolia (n=1), arthralgias (n=1); dysuria (n=1); pneumonia (n=1), acute renal failure (n=1); diarrhea (n=1); abdominal pain (n=1). One patient (5%) reported G≥4 AE (cerebral hemorrhage) not related to study treatment
