# Supplementary material for: Safety and antitumor activity of metformin plus lanreotide in patients with advanced gastro-intestinal or lung neuroendocrine tumors: the phase Ib trial MetNET2
Source: J Hematol Oncol. 2023 Dec 14;16:119. doi: 10.1186/s13045-023-01510-9 (PMC10722662; doi:10.1186/s13045-023-01510-9)
Supplement: Supplementary file 7 — Additional file 7. Table S5: Antitumor activity of the experimental treatment. [file 13045_2023_1510_MOESM7_ESM.docx]

**ADDITIONAL FILE 7**

**Table S5. Antitumor activity of the experimental treatment.**

| **Parameter** | **RECIST Version 1.1**  n. patients (%) | **Rate on percentage** |
| --- | --- | --- |
| ORR | 2 (10%) |  |
| 95% CI^a^ | 1- 32% |  |
| Best Overall Response |  |  |
| CR | 0 (0.0%) |  |
| PR | 2 (10%) |  |
| SD | 15 (75%) |  |
| PD | 3 (15%) |  |
| DCR | 17 (85%) |  |
| 95% CI^a^ | 62-96% |  |
| PFS, months^b^ |  |  |
| Median |  | 24 |
| 95% CI |  | 16-NE |
| Patients with events |  | 13 (65%) |
| PD |  | 12 (92.3%) |
| Death |  | 1 (7.7%) |
| PFS rate, % |  |  |
| 12 months |  | 75% |
| 95% CI^c^ |  | 58-97% |
| 24 months |  | 49% |
| 95% CI |  | 31-77% |
| 36 months |  | 37% |
| 95% CI |  | 20-67% |
| TTP, months^b^ |  |  |
| Median |  | 26 |
| 95% CI^c^ |  | 17-NE |
| Probability of no progression |  |  |
| 12 months |  | 80% |
| 95% CI^c^ |  | 64-100% |
| 24 months |  | 52% |
| 95% CI^c^ |  | 34-81% |
| 36 months |  | 39% |
| 95% CI^c^ |  | 22-71% |
| OS, months^b^ |  | Not reached |
| Median |  | First quartile: 42 |
| 95% CI^c^ |  | 26-NE |
| Death |  | 4 (20.0%) |
| OS rate, % |  |  |
| 12 months |  | 94% |
| 95% CI^c^ |  | 84-100% |
| 24 months |  | 89% |
| 95% CI^c^ |  | 76-100% |
| 36 months |  | 79% |
| 95% CI^c^ |  | 60-100% |

Legends: CR, complete response; DCR, disease control rate; NE, not estimable; ORR, objective response rate; OS, overall survival; PD, progressive disease; PFS, progression-free survival; PR, partial response; SD, stable disease; TTP, time to progression.

^a^ Calculated using exact method of binomial distribution (Clopper-Pearson method).

^b^ Kaplan-Meier method was used for estimating, PFS, TTP, and OS.

^c^ Based on Greenwood formula using log-log transformation.
