# Supplementary material for: Safety and antitumor activity of metformin plus lanreotide in patients with advanced gastro-intestinal or lung neuroendocrine tumors: the phase Ib trial MetNET2
Source: J Hematol Oncol. 2023 Dec 14;16:119. doi: 10.1186/s13045-023-01510-9 (PMC10722662; doi:10.1186/s13045-023-01510-9)
Supplement: Supplementary file 9 — Additional file 9. Fig. S3: Waterfall plot showing the relative modification of the estimated tumor volume (as per RECIST 1.1 criteria), achieved as the best tumor response. [file 13045_2023_1510_MOESM9_ESM.docx]

**ADDITIONAL FILE 9**

**Figure S3. Waterfall plot showing the relative modification of the estimated tumor volume (as per RECIST 1.1 criteria), achieved as the best tumor response.**

**
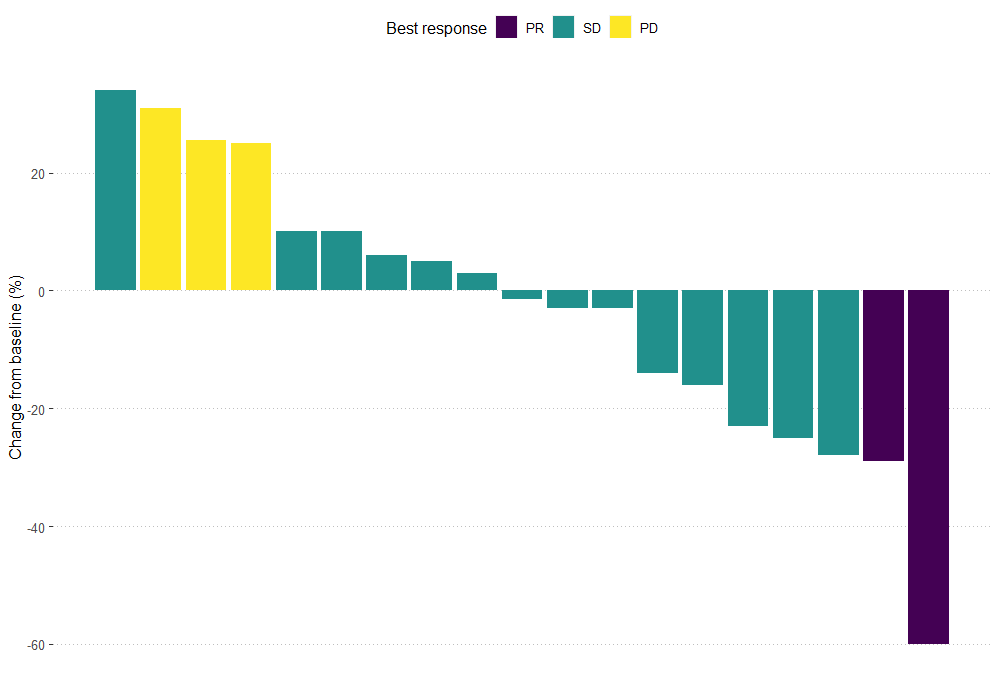
**

Duration of Lanreotide ATG plus Metformin treatment (months)

Legend: PR: partial response; SD: stable disease; PD: progressive disease.
