# Supplementary material for: Safety and antitumor activity of metformin plus lanreotide in patients with advanced gastro-intestinal or lung neuroendocrine tumors: the phase Ib trial MetNET2
Source: J Hematol Oncol. 2023 Dec 14;16:119. doi: 10.1186/s13045-023-01510-9 (PMC10722662; doi:10.1186/s13045-023-01510-9)
Supplement: Supplementary file 10 — Additional file 10. Figure S4: Kaplan-Meier curves for PFS among diabetic and non-diabetic patients (A). Kaplan Meier curves for PFS in normoglycemic, pre-diabetic and diabetic patients (B). [file 13045_2023_1510_MOESM10_ESM.docx]

**ADDITIONAL FILE 10**

**Figure S4.** Kaplan-Meier curves for PFS among diabetic and non-diabetic patients (**A**). Kaplan Meier curves for PFS in normoglycemic, pre-diabetic and diabetic patients (**B**).

**A B**

**
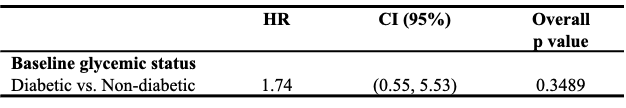

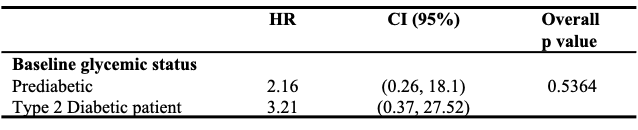

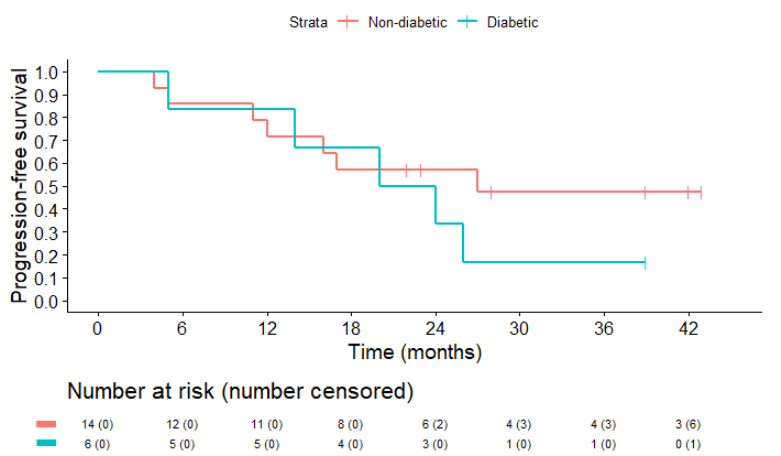

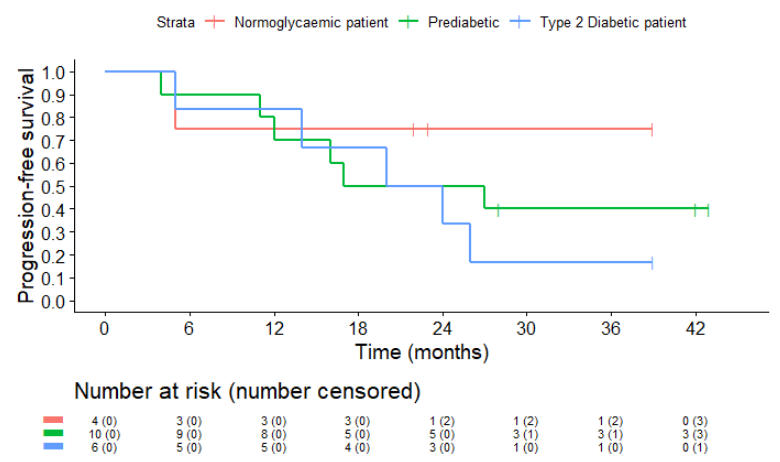
**

Legend: HR: Hazard Ratio; CI: Confidence Interval
