# Supplementary material for: Safety and antitumor activity of metformin plus lanreotide in patients with advanced gastro-intestinal or lung neuroendocrine tumors: the phase Ib trial MetNET2
Source: J Hematol Oncol. 2023 Dec 14;16:119. doi: 10.1186/s13045-023-01510-9 (PMC10722662; doi:10.1186/s13045-023-01510-9)
Supplement: Supplementary file 11 — Additional file 11. Table S6: Univariate Cox models for PFS. [file 13045_2023_1510_MOESM11_ESM.docx]

**ADDITIONAL FILE 11.**

**Table S6. Univariate Cox models for PFS.**

| **Variable** | **HR (95%CI)** | **P value** |
| --- | --- | --- |
| Male vs. Female | 0.36 (0.11, 1.14) | 0.083 |
| **Age category** |  |  |
| ≥65 vs. <65 | 1.64 (0.48, 5.54) | 0.429 |
| **Tumor grading** |  |  |
| G2 vs. G1 | 2.26 (0.59, 8.60) | 0.231 |
| **Ki-67** |  |  |
| >10 vs. <10 | 1.25 (0.26, 6.11) | 0.780 |
| **Functioning tumor** |  |  |
| No vs. Yes | 0.1 (0.02, 0.44) | 0.003 |
| **Number of metastatic sites** |  |  |
| ≥2 vs. 1 | 1.97 (0.43, 9.12) | 0.384 |
| **Liver metastasis** |  |  |
| Yes vs. No | 2.29 (0.29, 17.89) | 0.431 |
| **Liver involvement** |  |  |
| Liver only vs. Not liver only | 1.46 (0.32, 6.75) | 0.627 |
| **Liver burden** |  |  |
| ≥ 25 vs < 25% | 1.09 (0.29, 4.17) | 0.8971 |
| **ECOG PS** |  |  |
| 1 vs. 0 | 0* (0, >1000) | 0.796 |
| **Primary tumor resection** |  |  |
| No vs. Yes | 1.38 (0.3, 6.38) | 0.676 |
| **Liver treatment** |  |  |
| No vs. Yes | 1.81 (0.4, 8.29) | 0.443 |
| **Prior treatment lines** |  |  |
| 1 vs. 0 | 1.5 (0.3, 7.45) | 0.476 |
| ≥2 vs. 0 | 2.7 (0.48, 14.84) |  |
| **DM status** |  | 0.348 |
| DM vs non-DM | 1.74 (0.55, 5.53) |  |
| **Baseline plasma triglyceride concentration** |  |  |
| >150 vs. <150 mg/dL | 0.29 (0.06, 1.34) | 0.1140 |

Legends: CI: confidence interval; DM: diabetes mellitus; ECOG PS: Eastern Cooperative Oncology Group Performance Status; G: grade; HR: hazard ratio.

*The wide confidence interval is due to the low number of patients with ECOG PS=1 (2/20 patients).
