# Supplementary material for: Safety and antitumor activity of metformin plus lanreotide in patients with advanced gastro-intestinal or lung neuroendocrine tumors: the phase Ib trial MetNET2
Source: J Hematol Oncol. 2023 Dec 14;16:119. doi: 10.1186/s13045-023-01510-9 (PMC10722662; doi:10.1186/s13045-023-01510-9)
Supplement: Supplementary file 12 — Additional file 12. Figure S5. Oncoprint of tumor genomic alterations in patients enrolled in Met-NET2 trial. Mutations are classified as missense mutations (green), truncating mutations (dark grey), or no alterations (light grey). [file 13045_2023_1510_MOESM12_ESM.docx]

**ADDITIONAL FILE 12**

**Figure S5.** Oncoprint of tumor genomic alterations in patients enrolled in Met-NET2 trial. Mutations are classified as missense mutations (green), truncating mutations (dark grey), or no alterations (light grey).

**
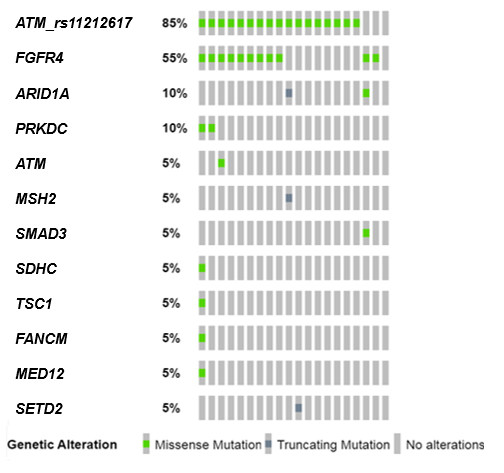
**
