# Supplementary material for: Safety and antitumor activity of metformin plus lanreotide in patients with advanced gastro-intestinal or lung neuroendocrine tumors: the phase Ib trial MetNET2
Source: J Hematol Oncol. 2023 Dec 14;16:119. doi: 10.1186/s13045-023-01510-9 (PMC10722662; doi:10.1186/s13045-023-01510-9)
Supplement: Supplementary file 13 — Additional file 13. Table S7. Distribution of genomic biomarkers according to diabetic status. [file 13045_2023_1510_MOESM13_ESM.docx]

**ADDITIONAL FILE 13**

**Table S7. Distribution of genomic biomarkers according to diabetic status.**

| **Variable** | Non-diabetic | Diabetic | Overall | *P-* Value * |
| --- | --- | --- | --- | --- |
| **Tumor genomic alteration** |  |  |  | 0.5211 |
| wild-type tumor genes | 1 (7.1%) | 1 (16.7%) | 2 (10.0%) |  |
| ≥ 1 gene mutation | 13 (92.9%) | 5 (83.3%) | 18 (90.0%) |  |
| ***FGFR4* polymorphism *rs351855*** |  |  |  | 0.6424 |
| Gly/Gly^388^ FGFR4 wild type | 7 (50.0%) | 2 (33.3%) | 9 (45.0%) |  |
| Gly/Arg^388^ FGFR4 | 7 (50.0%) | 4 (66.7%) | 11 (55.0%) |  |
| **Allelic frequencies of *rs351855*** |  |  |  | 0.7042 |
| Allele A | 7 (25.0%) | 4 (33.3%) | 11 (27.5%) |  |
| Allele G | 21 (75%) | 8 (66.7%) | 29 (72.5%) |  |
| **DNA repair mutated genes•** |  |  |  | 1.0000 |
| wild-type tumour genes | 10 (71.4%) | 4 (66.7%) | 14 (70.0%) |  |
| ≥ 1 gene mutation | 4 (28.6%) | 2 (33.3%) | 6 (30.0%) |  |
| ***ATM* polymorphism *rs11212617*** |  |  |  | 0.1765 |
| Genotypes C/C | 2 (14.3%) | 1 (16.7%) | 3 (15%) |  |
| Genotypes A/C | 8 (57.1%) | 1 (16.7%) | 9 (45%) |  |
| Genotypes A/A | 4 (28.6%) | 4 (66.6%) | 8 (40%) |  |
| **Allelic frequencies of *rs11212617*** |  |  |  | 0.4774 |
| Allele A | 16 (57.1%) | 9 (75.0%) | 25 (62.5%) |  |
| Allele C | 12 (42.9%) | 3 (25.0%) | 15 (37.5%) |  |

* The associations were tested using Wilcoxon-Mann-Whitney test for continuous data and Fisher’s exact test for categorical data. Unknown and missing values were excluded from the statistical tests.

• We enclosed patient having at least one or more mutated DNA repair genes in tumro cells among *ARID1A*, *ATM*, *SETD2*, *PRKDC*.
