# Supplementary material for: Safety and antitumor activity of metformin plus lanreotide in patients with advanced gastro-intestinal or lung neuroendocrine tumors: the phase Ib trial MetNET2
Source: J Hematol Oncol. 2023 Dec 14;16:119. doi: 10.1186/s13045-023-01510-9 (PMC10722662; doi:10.1186/s13045-023-01510-9)
Supplement: Supplementary file 14 — Additional file 14. Figure S6: Figure S6. Kaplan-Meier curves for PFS according to the presence of alterations in genes involved in DNA repair (ARID1A, ATM, SETD2, PRKDC) (A), FGFR4 gene polymorphism rs351855 (B), ATM allelic variants: A/A vs. A/C vs. C/C (C), or A/A vs. A/C or C/C (D). [file 13045_2023_1510_MOESM14_ESM.docx]

**ADDITIONAL FILE 14**

**Figure S6.** Kaplan-Meier curves for PFS according to the presence of alterations in genes involved in DNA repair (*ARID1A*, *ATM*, *SETD2*, *PRKDC*) (**A**), *FGFR4* gene polymorphism rs351855 (**B**), *ATM* allelic variants: A/A vs. A/C vs. C/C (**C**), or A/A vs. A/C or C/C (**D**).

**A B**


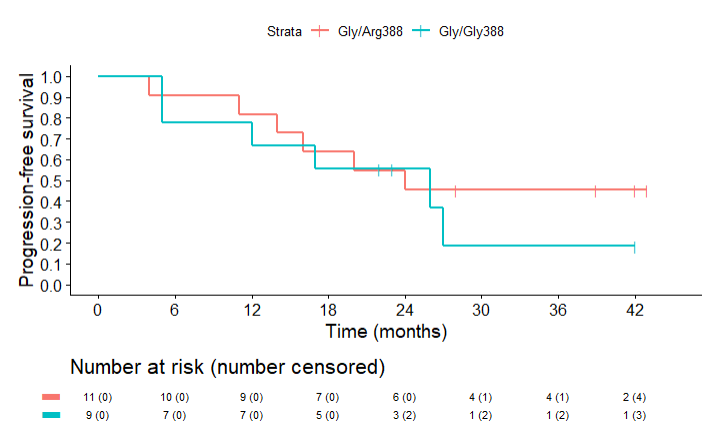

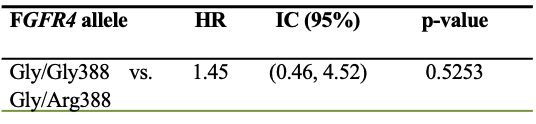

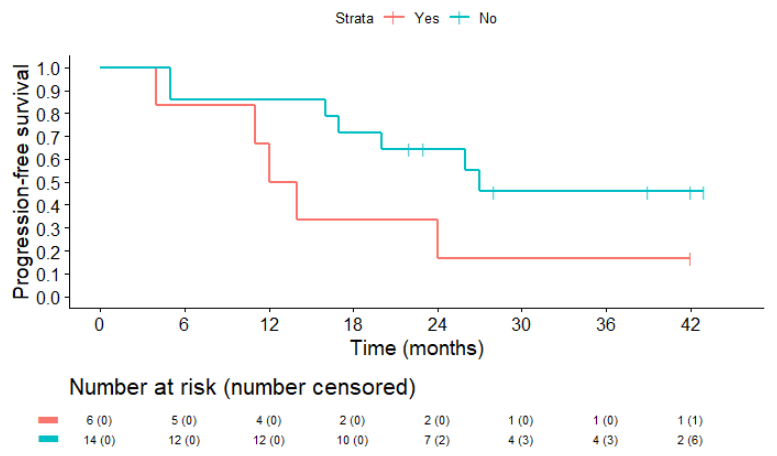

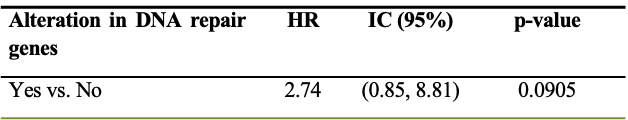


**
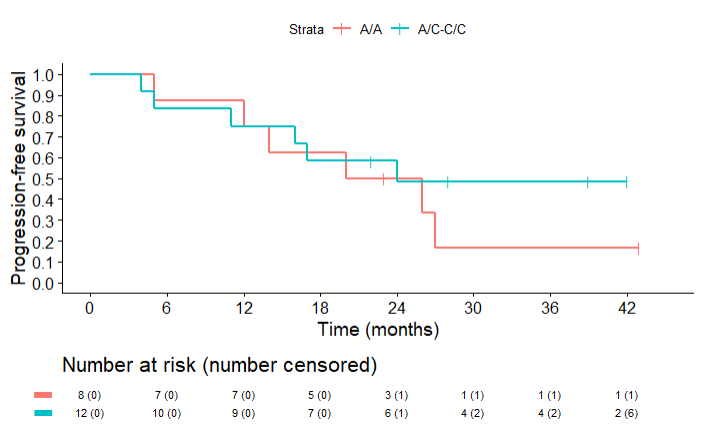

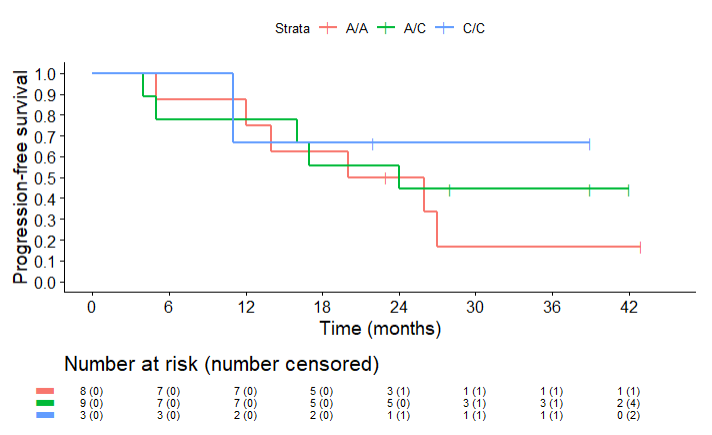
C D**

**
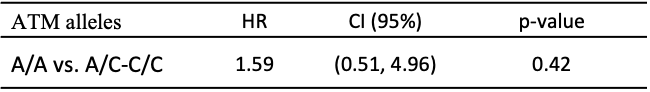

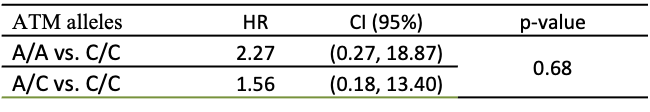
**

Legend: HR: Hazard Ratio; CI: Confidence Interval
