# Supplementary material for: Safety and antitumor activity of metformin plus lanreotide in patients with advanced gastro-intestinal or lung neuroendocrine tumors: the phase Ib trial MetNET2
Source: J Hematol Oncol. 2023 Dec 14;16:119. doi: 10.1186/s13045-023-01510-9 (PMC10722662; doi:10.1186/s13045-023-01510-9)
Supplement: Supplementary file 15 — Additional file 15. Table S8: Longitudinal kinetics of the indicated metabolic blood parameters and body mass index (BMI). [file 13045_2023_1510_MOESM15_ESM.docx]

**ADDITIONAL FILE 15**

**Table S8.** **Longitudinal kinetics of the indicated metabolic blood parameters and body mass index (BMI).**

| **Variable** | **Baseline** N=20^*^ | **6 months**  N=20^*^ | **9 months**  N=20^*^ | **12 months** N=20^*^ | **18 months** N=20^*^ | **24 months** N=20^*^ | ***P* value**** |
| --- | --- | --- | --- | --- | --- | --- | --- |
| **Glucose (mg/dL)** | 109.00 (13.93) | 111.22 (14.77) | 112.47 (12.24) | 116.07 (15.75) | 119.99 (22.68) | 125.12 (22.8) | 0.202 |
| Missing | 0 | 0 | 3 | 5 | 8 | 12 |  |
| **Insulin (pmol/L)** | 7.75 (4.79) | 5.32 (4.02) | 6.31 (4.18) | 6.13 (3.38) | 5.88 (4.08) | 14.51 (19.90) | 0.615 |
| Missing | 0 | 0 | 3 | 5 | 9 | 13 |  |
| **HbA1c (mmol/mol)** | 39.35 (4.03) | 38.83 (4.62) | 39.60 (5.64) | 41.20 (4.72) | 40.20 (4.02) | 44.50 (7.01) | 0.213 |
| Missing | 0 | 0 | 5 | 5 | 10 | 14 |  |
| **Homa-IR index (%)** | 2.09 (1.35) | 1.48 (1.17) | 1.78 (1.31) | 1.80 (1.13) | 1.91 (1.79) | 4.71 (6.15) | 0.613 |
| Missing | 0 | 0 | 3 | 5 | 9 | 13 |  |
| **Cholesterol (mmol/L)** | 185.95 (32.16) | 175.68 (27.74) | 170.47 (26.72) | 177.87 (28.08) | 178.67 (31.91) | 170.57 (24.27) | 0.726 |
| Missing | 0 | 0 | 3 | 5 | 8 | 13 |  |
| **Triglycerides (mmol/L)** | 112.32 (49.11) | 111.55 (47.96) | 110.88 (57.00) | 109.60 (52.81) | 111.42 (59.29) | 129.83 (122.21) | 0.993 |
| Missing | 1 | 0 | 3 | 5 | 8 | 14 |  |
| **BMI (kg/m^2^)** | 23.35 (3.59) | 22.83 (3.78) | 22.43 (3.78) | 22.33 (4.12) | 22.53 (2.91) | 22.74 (2.28) | 0.984 |
| Missing | 0 | 0 | 3 | 5 | 9 | 13 |  |

*Mean (SD)

******The associations were tested using repeated measures, one-way analysis of variance (ANOVA) test. Unknown and missing values were excluded from the statistical tests.

Legends: N: number of patients; BMI: body max index; HOMA-IR index: Homeostatic Model Assessment of Insulin Resistance.
