# Supplementary material for: Safety and antitumor activity of metformin plus lanreotide in patients with advanced gastro-intestinal or lung neuroendocrine tumors: the phase Ib trial MetNET2
Source: J Hematol Oncol. 2023 Dec 14;16:119. doi: 10.1186/s13045-023-01510-9 (PMC10722662; doi:10.1186/s13045-023-01510-9)
Supplement: Supplementary file 16 — Additional file 16. Table S9: Association between early reduction in metabolic parameters and the risk of disease progression (Hazard Ratio). [file 13045_2023_1510_MOESM16_ESM.docx]

**ADDITIONAL FILE 16**

**Table S9.** **Association between early reduction in metabolic parameters and the risk of disease progression (Hazard Ratio).**

| **Variable** | **Cutpoint** | **HR (95%CI)** | *P-* Value * |
| --- | --- | --- | --- |
| HOMA-IR Index | 1.05 | 5.91 (0.76, 46.07) | 0.09 |
| Insulin | 0.55 | 2.67 (0.85, 8.38) | 0.09 |
| Cholesterol | 23.50 | 5.12 (0.65, 40.42) | 0.12 |
| Hb1Ac | 4.00 | 0.27 (0.05, 1.42) | 0.12 |
| Triglycerides | -28.50 | 0.23 (0.03, 1.86) | 0.17 |
| Glycemia | -2.00 | 1.87 (0.59, 5.96) | 0.29 |
| BMI | 0.68 | 0.55 (0.18, 1.72) | 0.30 |

*The associations were tested using univariable Cox proportional hazard models and reported as Hazarad Ratios and Wald P test values. Unknown and missing values were excluded from the statistical tests.

Legends: HR: Hazard Ratio; HOMA-IR index: Homeostatic Model Assessment of Insulin Resistance; BMI: body max index;
